# Heterochiasmy and the establishment of *gsdf* as a novel sex determining gene in Atlantic halibut

**DOI:** 10.1371/journal.pgen.1010011

**Published:** 2022-02-08

**Authors:** Rolf Brudvik Edvardsen, Ola Wallerman, Tomasz Furmanek, Lene Kleppe, Patric Jern, Andreas Wallberg, Erik Kjærner-Semb, Stig Mæhle, Sara Karolina Olausson, Elisabeth Sundström, Torstein Harboe, Ragnfrid Mangor-Jensen, Margareth Møgster, Prescilla Perrichon, Birgitta Norberg, Carl-Johan Rubin

**Affiliations:** 1 Institute of Marine Research, Bergen, Norway; 2 Uppsala University, Uppsala, Sweden; 3 Institute of Marine Research, Storebø, Norway; University of Georgia, UNITED STATES

## Abstract

Atlantic Halibut (*Hippoglossus hippoglossus*) has a X/Y genetic sex determination system, but the sex determining factor is not known. We produced a high-quality genome assembly from a male and identified parts of chromosome 13 as the Y chromosome due to sequence divergence between sexes and segregation of sex genotypes in pedigrees. Linkage analysis revealed that all chromosomes exhibit heterochiasmy, i.e. male-only and female-only meiotic recombination regions (MRR/FRR). We show that FRR/MRR intervals differ in nucleotide diversity and repeat class content and that this is true also for other Pleuronectidae species. We further show that remnants of a Gypsy-like transposable element insertion on chr13 promotes early male specific expression of *gonadal somatic cell derived factor* (*gsdf*). Less than 4.5 MYA, this male-determining element evolved on an autosomal FRR segment featuring pre-existing male meiotic recombination barriers, thereby creating a Y chromosome. Our findings indicate that heterochiasmy may facilitate the evolution of genetic sex determination systems relying on linkage of sexually antagonistic loci to a sex-determining factor.

## Author summary

Even closely related fish species can have different sex chromosomes, but this turn-over of sex determination systems is poorly understood. Here, we used large-scale genome sequencing to determine the DNA sequence of the Atlantic halibut chromosomes and compared sequencing data from males and females to identify the sex chromosomes. We show that males have much higher gene activity of the gene *gonadal somatic cell derived factor* (*gsdf*), which is located on the sex chromosomes and has a role in testicular development. The genome contains many mobile DNA sequences, transposable elements (TEs), one placed in front of *gsdf*, enhancing its activity. This made *gsdf* the sex determining factor, thereby creating a new Y-chromosome. We further describe how all Atlantic halibut chromosomes behave similar to sex chromosomes in that most regions only recombine in one sex. This phenomenon may contribute to the rapid turn-over of genetic sex determination systems in fish. Our results highlight the molecular events creating a new Y-chromosome and show that the new Atlantic halibut Y was formed less than 4.5 million years ago. Future studies in Atlantic halibut and closely related species can shed light on mechanisms contributing to sex chromosome evolution in fish.

## Introduction

In contrast to the highly conserved mammalian and avian sex determination systems, fish display much more plasticity and turnover of sex chromosomes and sex determination systems, with many already described systems for genetic control of sex being young in comparison to their mammalian and avian counterparts [1]. Furthermore, teleosts display a rich diversity of sex-determining mechanisms, both environmental and genetic [2], with some species being sequential hermaphrodites spending part of their lives as males and part as females [3]. As for genetic control of sex, both XX/XY and ZZ/ZW systems have been described in teleosts, sometimes with closely related species having differing systems, such as for sticklebacks [4], tilapias [5], swordtail fish [6] and mosquitofish [7]. Several master sex determining (MSD) genes have been described in teleosts, most of which belong to one of three protein families (DMRT, SOX, and TGF-ß and its signaling pathway) [8], and these have originated either from sub- or neo-functionalization of duplicated genes, or by allelic diversification [1]. Exceptions to these protein families are found for salmonids, where *sdy* is the MSD gene [9] and recently the *breast cancer anti-estrogen resistance protein 1* (*BCAR1*) and *bone morphogenetic protein receptor type-1B* (*bmpr1b*) have been reported as MSD genes in catfish (*Ictalurus punctatus*) [10] and Atlantic herring (*Clupea harengus*) [11], respectively. Thus, a wide range of systems have evolved for genetic control of sex determination in fish and additional complexity is added by environmental variables contributing to sex determination in some species.

A widely used model for sex chromosome evolution features progressively increasing divergence between X and Y, or Z and W, chromosomes as structural barriers to recombination, such as genomic structural variants (SVs), become acquired on Y or Z. Under this model, the sex-limited chromosome will accumulate deleterious mutations and lose genetic material not essential for the male or female phenotype in X/Y and Z/W systems, respectively. In contrast to heteromorphic sex chromosomes, several reptiles, amphibians and fishes show homomorphic sex chromosomes with little or no signs of degeneration or even multiple short sex-determining regions on different chromosomes [12] or recombining in only in the heterogametic sex [13]. In strong contrast to the model of physical recombination barriers, several recent studies have implicated that heterochiasmy, i.e. sex-preferential regions of meiotic recombination can play an important role in the evolution of sex chromosomes [13–16].

Righteye flounders (*Pleuronectidae*) have variable genetic sex determinations systems with both XX/XY and ZZ/ZW systems represented [17]. The Atlantic halibut (*Hippoglossus hippoglossus*) has an XX/XY, male heterogametic, sex determination system [18]. A linkage study previously identified the Atlantic halibut sex chromosome linkage groups and fine mapped the Y-chromosome to a larger marker interval on one linkage group [19] and another study revealed sex differences in recombination patterns, although using a sparse set of microsattelite markers analysed in the absence of a genome assembly [20]. The Pacific Halibut (*Hippoglossus stenolepis*), which diverged from the Atlantic halibut 1.7–4.5 million years ago [21], has a ZZ/ZW system [22]. The differing systems within the *Hippoglossus* genus indicates that at least one of Atlantic- and Pacific halibut must have evolved a novel sex determination system after their split which happened in the last 4.5 MY and that the Atlantic halibut sex-determining locus evolved recently.

In this project we used an Oxford Nanopore Technologies (ONT) MinION device to sequence long native DNA molecules from a single male Atlantic halibut and used the assembled contigs in conjunction with Chromatin Conformation Capture sequencing (HiC) data to produce a chromosome-scale scaffolded genome assembly. To identify and characterize the sex chromosome we mapped male and female DNA resequencing and whole embryo RNA-sequencing reads onto the assembly to screen for divergent genetic markers and differential expression between the sexes. Thus, our high-quality Atlantic halibut genome assembly offers great potential to study the emergence of a young genetic sex determination system and the evolution of sex chromosomes.

## Results

### Genome assembly and annotation

We used nanopore sequencing in combination with Illumina sequencing of 10X Genomics Chromium-, paired-end- and HiC libraries to generate a chromosome-scale haploid assembly of the Atlantic halibut. The final scaffolded assembly (IMR_hipHip.v1) had a scaffold N50 of 27 Mb (S1A and S1B Fig) and this assembly was evaluated using BUSCO using 4584 genes in *Actinopterygii* odb9 data set, annotating 95.4% of analyzed genes as “Complete” in the genome assembly. The assembly is organized into 24 larger scaffolds containing 97.6% of the assembled sequence (S2 Fig), corresponding to the chromosomes inferred from karyotyping [23] and linkage analysis [20]. A number of smaller contigs and scaffolds, together comprising only 2.4% of the assembled sequence was left as unplaced. We named the scaffolds by mapping microsatellite primer sequences from a published genetic linkage map for Atlantic halibut [20] to IMR_hipHip.v1. Genome to genome alignment of IMR_hipHip.v1 to a recently finished reference genome of the Pacific halibut (NCBI accession GCF_013339905.1) revealed a largely co-linear genome organization, with few large-scale disagreements (S1C Fig) as expected due to the recent split of the two species. To generate RNA-seq data for genome annotation and to obtain transcriptomic data describing the early stages of development, we used samples from different time points throughout embryonic development from newly fertilized to 54 days post fertilization (dpf). For the first four stages (1 hour post fertilization, 5 dpf, 8 dpf and 12 dpf), four pools of eight individuals were collected and subjected to RNA extraction and Illumina HiSeq mRNA-sequencing. For the 28 dpf, 43 dpf and 54 dpf, four individual fish were sampled and sequenced for each stage. Genome annotation based on the RNA-sequencing identified 18,314 genes, of which 18,092 (98.8%) significantly matched with proteins in public databases.

### Identification of the Atlantic halibut sex chromosome

**Fig 1:**
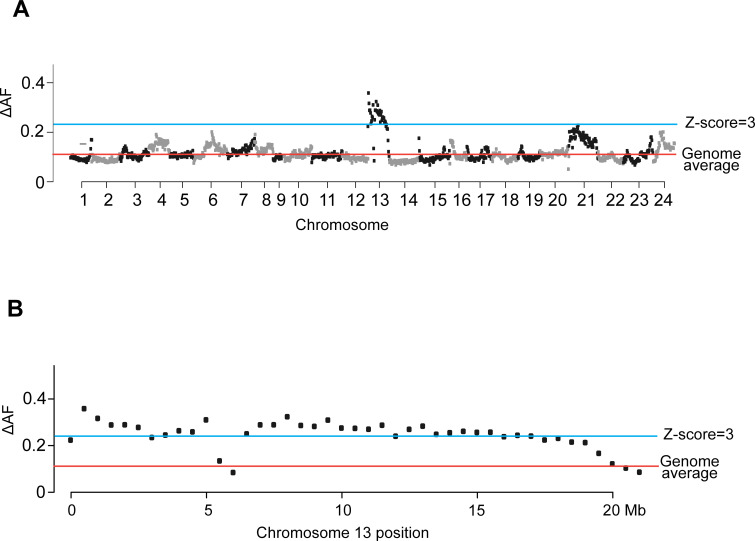
Genetic differentiation between phenotypic males and females highlights chr13 as the putative Y-chromosome. **A** Average male vs. female ΔAF for 500 kb windows along each assembled chromosome. The genome-average ΔAF is indicated as a horizontal line together with a line indicating Z-score = 3, i.e. the ΔAF-value corresponding to 3 SD above the genome average). **B** Average ΔAF for 500 kb windows along chr13 with genome average ΔAF and 3 SD above genome average ΔAF indicated by horizontal lines.

In order to identify and characterize the sex chromosomes we sampled and extracted and pooled DNA from ten full siblings of each phenotypic sex and sequenced these separately using Illumina HiSeq sequencing. The sequencing data was mapped against the IMR_hipHip.v1 assembly and the sequence differentiation between male and female pools was determined for windows of 500 kb along each chromosome using delta allele frequency (ΔAF) by comparing male- and female allele frequencies (ΔAF = abs(maleAF-femaleAF)). This screen revealed chromosome 13 as the most diverged between males and females (Fig 1A), in agreement with a previously published linkage study using RAD-sequencing [19]. The divergence between phenotypic males and females on chr13 is not uniform, but gradually declines along the length of the chromosome, with the end being no more divergent than autosomes (Fig 1B). We extracted SNPs that had a male vs. female allele frequency difference of >40% while being fixed for one allele in the female pool. Genome-wide 8,566 SNPs showed this pattern, 5,715 of which were located on chr13.

### Identification of the Atlantic halibut sex determining gene

**Fig 2:**
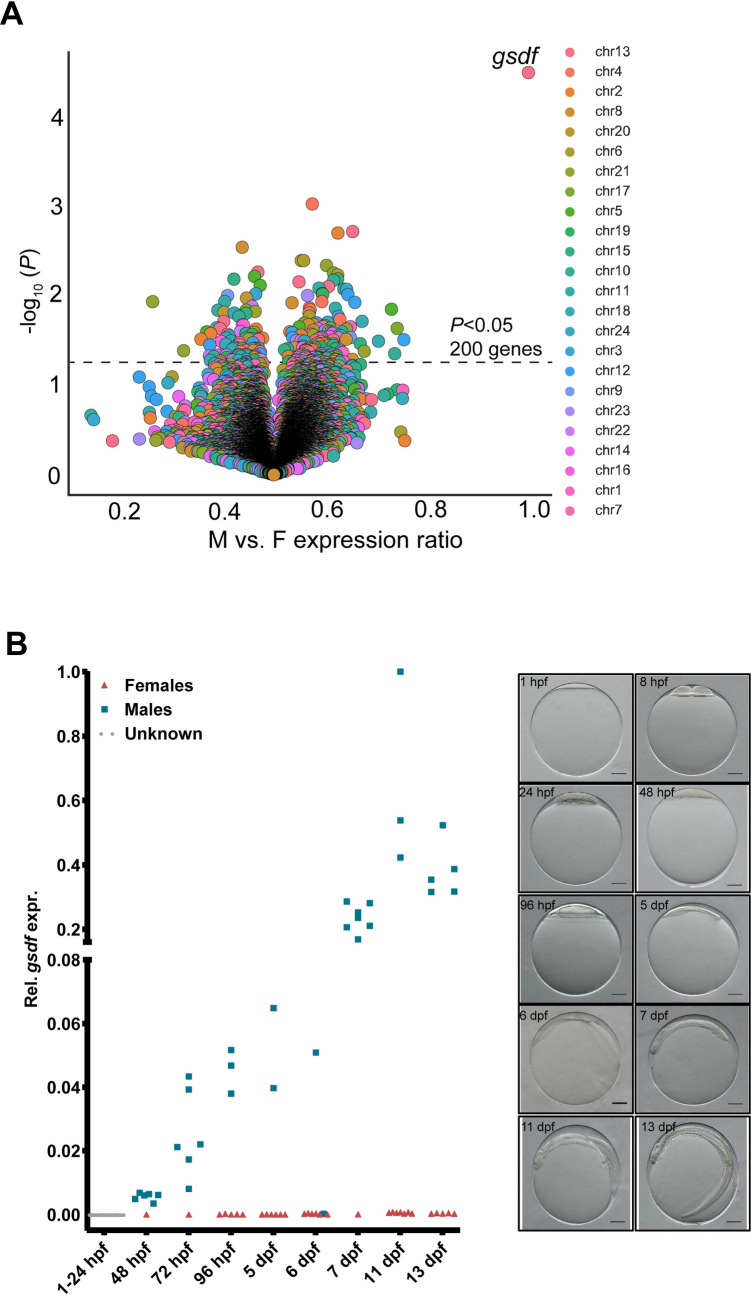
Identification of *gsdf* as the Atlantic halibut sex determining gene. **A** Volcano plot representation of differential expression (DE) between individuals stratified by chr13 genotyping-based sex assignment. Dot colors indicate the chromosome from which genes are expressed. The most highly differentially expressed gene, *gsdf*, is indicated in the upper right corner. **B** qPCR result showing the sex specific *gsdf* expression (relative to *gtf3c6*) across developmental stages. **C** Pictures of fish representing the developmental stages sampled for gene expression analyses; 1 hpf to 13 dpf.

RNA-seq data was generated from 12 whole individuals covering three developmental stages with the assumption that both genetic sexes would be represented. As we were unable to infer phenotypic sex for these samples, an unbiased screen could involve searching for the most variably expressed genes among the 12 individuals, but after conducting a Principal Component Analysis we concluded that the samples clustered strongly by development stage (S3 Fig). An alternative strategy could involve screening for variable gene expression between known phenotypic sexes during gonadal differentiation. As known phenotypic sex was unavailable, we determined the genetic sex of the RNA-seq samples by mapping the RNA-seq samples to the genome, then intersecting observed RNA-seq alleles with the 5,715 SNPs on chr13 differentiating males from females. Hierarchical clustering of RNA-seq alleles observed at intersected SNP positions (S4 Fig), shows clearly that 7 samples were genetically male (XY) and 5 were genetically female (XX). We then produced a Volcano plot showing the global genetic male vs. female expression ratio plotted against the statistical significance of a differential expression (DE) between sexes (Fig 2A). The statistically most significantly DE gene (two-sided t-test) was *gsdf*, previously shown to be the male-determining MSD gene in Luzon ricefish and sablefish [24,25]. Furthermore, among genes previously identified as MSD genes in other fish species [8], only *gsdf and bmpr1b* [11] reside on the herein defined chrY (chr13 FRR) in Atlantic halibut (*gsdf* at 8.5 Mb and *bmpr1b* at 12.1 Mb). *Bmpr1b* was however not differentially expressed between males and females. Among 12 RNA-seq individuals the 5 XX fish had almost no *gsdf* expression, while the 7 XY fish all had high expression, consistent with Gsdf being a male determining factor. To verify sex specific expression of *gsdf* during development and to obtain better temporal resolution of expression levels, we performed quantitative PCR (qPCR) on PCR sex-determined samples (Fig 2B and 2C). RNA was extracted from individual embryos from 11 development stages, ranging from 1 hour post fertilization (hpf) to 13 dpf. A PCR assay based on SNPs in the 3’UTR of *brx* (chr13:9125004–9125007) was used to determine genetic sex of samples from 48 hpf and onwards (S5 Fig). The qPCR results show that the expression of *gsdf* starts in males already at 48 hpf, and from 7 dpf onwards there is a clear increase in expression compared to the earlier stages. For one individual sampled at 6 dpf determined as a genetic male, we could not detect any expression, possibly due to environmental factors having the chance to override the genotype in the determination of phenotypic sex in the species as is the case with several flounder species [17,26], including Atlantic halibut [27]. *In silico* searches for conserved genes located up- and downstream of *gsdf* in other species confirmed the *gsdf* identity. (S6 Fig).

### Most of the Atlantic halibut genome is subject to male-only or female-only meiotic recombination

**Fig 3:**
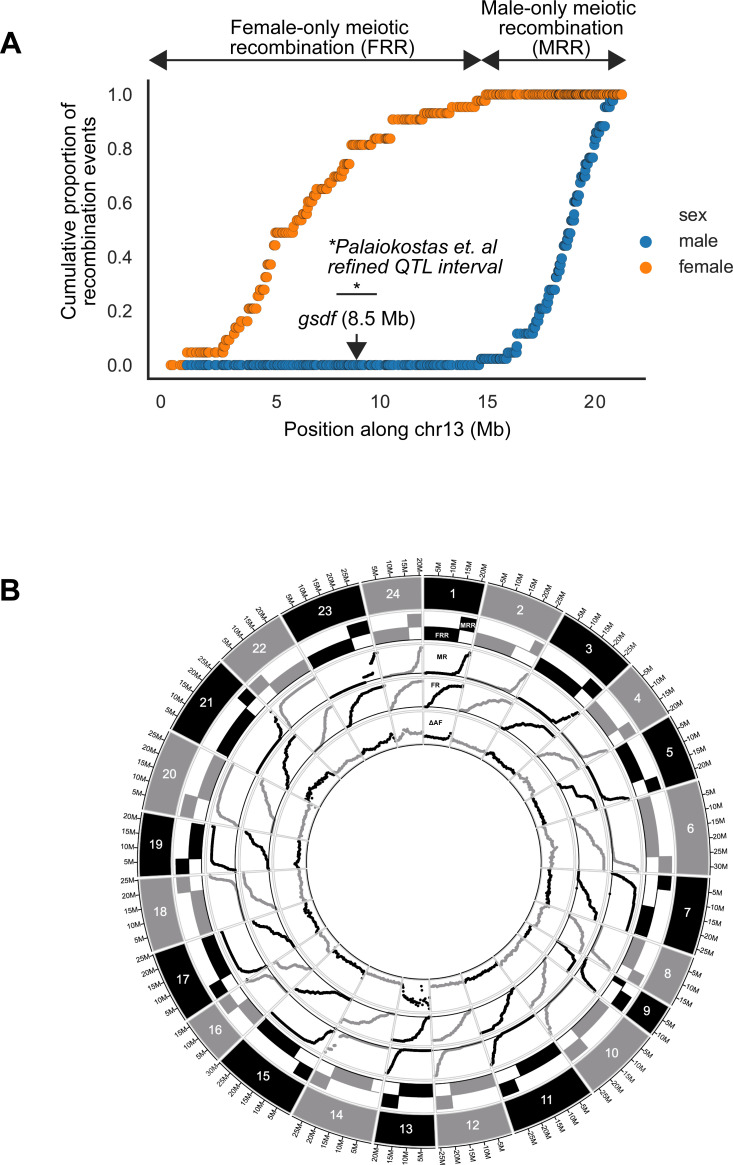
Cumulative proportion of all recombination events occurring during male and female meiosis in a two-pedigree intercross. **A** chr13 with *gsdf* location indicated. *Mapping interval of markers most highly associated with sex in a previous study [19]. **B** Male and female linkage maps differ strongly genome-wide. The outermost track visualizes individual chromosomes. Locations of defined Male-only and Female-only meiotic Recombination Regions (MRRs and FRRs) are shown as alternating black/grey rectangular tracks beneath each chromosome. All 24 chromosomes exhibit clear sex-restricted recombination intervals as shown in the tracks labeled MR and FR, showing the cumulative proportions (range 0–1) of all recombination events on each chromosome for males (MR) and females (FR). The innermost track, labeled ΔAF, shows the male vs. female delta allele frequency for 500kb windows along each chromosome (range 0–0.36).

In order to validate chr13 as the Atlantic halibut sex chromosome we downloaded the pedigree RAD-sequencing data previously used to assign linkage group 13 as X/Y [19], aligned the data to our assembly IMR_hipHip.v1, called SNPs and investigated genotypes by sex phenotype in the pedigrees. The pedigree analysis verified our divergence-based assignment of IMR_hipHip.v1 chr13 as the sex chromosome and 90/94 F1 individuals were in agreement with regard to the association between phenotypic sex and chr13 genotype. A similar observation was made for the embryonal qPCR analysis where one out of 34 genetic males (*brx*-assay) had no *gsdf* expression. The reason for the non-perfect association between genetic- and phenotypic sex could be erroneous annotation of the downloaded RAD-seq dataset or a logical consequence of non-perfect penetrance of young genetic sex determination systems. In our genome assembly-guided linkage analysis, we detected suppression of meiotic recombination along a large part of chr13 in male germ cells as would be expected in an X/Y system. Surprisingly, the majority of the sex chromosome pair exhibited strict sex specific meiotic recombination. We term such regions Male-only or Female-only Regions of meiotic Recombination (MRR/FRR) (Fig 3A). The male- and female genetic maps for chr13 both sum up to 49 cM but the recombining intervals are nonoverlapping. None of the 90 F1 offspring used for analysis carried a male meiotic recombination in the first ~15 Mb, while all detected female meiotic recombination events on chr13 occurred in this region. Thus, the first ~15 Mb of chr13 behaves like a X/Y-linked region, with high male vs. female divergence (Fig 1B) and nonexistent meiotic recombination in males (FRR). The later part of chr13 (~15 Mb until chromosome end) behaves like a pseudoautosomal region (PAR), but only recombines in males (X/Y recombination), rendering sequence diversity in this part of the chromosome shared between X and Y.

Even more surprising than the meiotic recombination desert observed for chr13 in females was the observation that all chromosomes clustered into more or less strict MRR/FRR regions (Figs 3B and S7A, S1 Table). We limited our analysis to the placed chromosome part of the genome (chromosomes 1–24), spanning 596 Mb out of the total 611 Mb, and defined FRRs and MRRs according to our classification outlined in S2 Table. Genome-wide, this annotation defined 407 Mb (68% of the sequence) as FRRs due to preferential recombination in females and 154 Mb as MRRs due to preferential recombination in males (26% of the sequence). We investigated what distinguishes an MRR from an FRR and concluded that for all chromosomes except chr9, MRRs are much smaller than FRRs while retaining approximately the same number of recombination events (S7B Fig). Only ~6% of the sequence fell outside of this classification because both sexes recombined at similar frequencies or because meiotic recombination was not observed in either sex.

### Nucleotide diversity and repeat content are correlated with sex-restricted recombination

**Fig 4:**
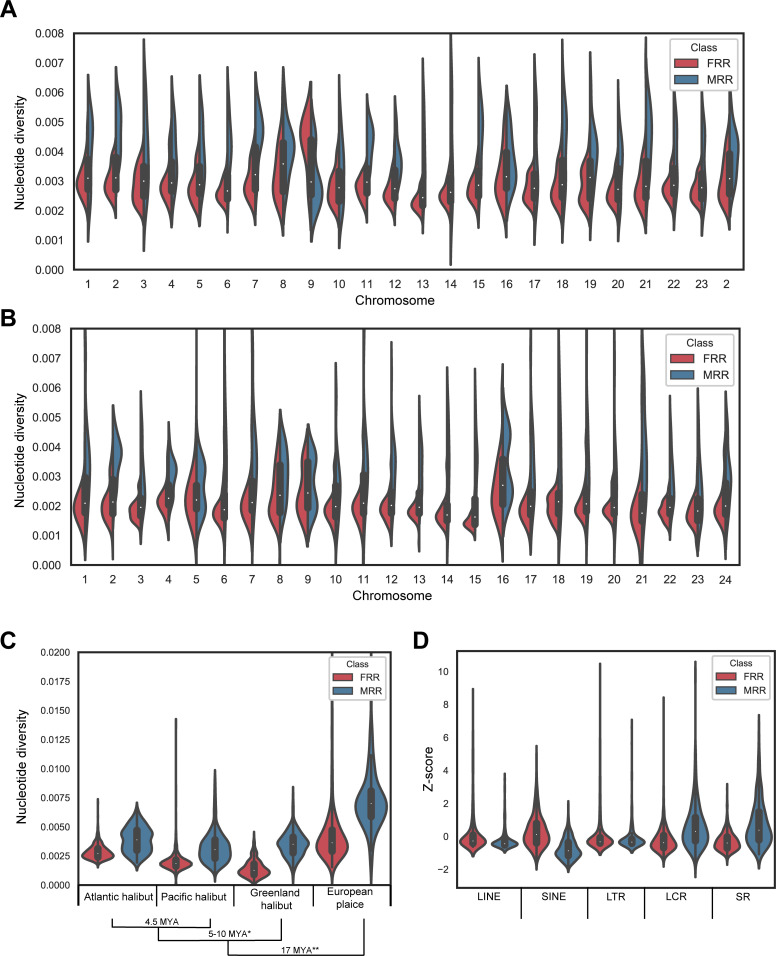
Nucleotide diversity (π) and repeat content is correlated with sex-restricted recombination throughout the genome. **A** π distribution in the Atlantic halibut genome, contrasting regions of male-only vs. female-only meiotic recombination, MRRs and FRRs, respectively. **B** Distribution of Pacific Halibut π estimates for regions mapped back to the Atlantic halibut genome, classified as either MRRs or FRRs. Chromosomal and MRR/FRR assignments were extracted from the Atlantic halibut genome assembly coordinates. **C** Genome-wide distributions of estimated π for Atlantic-, Pacific- and Greenland halibut and European plaice in regions corresponding to herein defined Atlantic halibut MRRs and FRRs. Numbers above MRR distributions indicate, within species, how much higher π is in MRR regions compared to FRR regions. * The time since divergence of Greenland halibut from *Hippoglossus* was estimated from [28]. ** Divergence estimate for European plaice was obtained from TimeTree [29]. **D** Genome-wide distributions of five repeat types in MRRs and FRRs. LINE and SINE represent Long- and short interspersed nucleotide elements, respectively. LTR indicates Long Terminal Repeats. LCR and SR indicate low complexity- and simple repeats, respectively. The Y-axis shows densities of different repeat types after Z-score transformations in relation to genome averages.

MRRs, with the exception of chr9, exhibit higher nucleotide diversity (π) than their FRR counterparts (Fig 4A). Recombination rate is correlated with π (S8 Fig) and chr13 is an outlier with the largest MRR over FRR π ratio among all chromosomes, presumably due to a bottleneck associated with the establishment of the new Y-chromosome. Furthermore, the pool-sequenced male and female individuals came from a single cross between one female and one male, meaning that less chrX and chrY genetic diversity was transmitted to female and male offspring, respectively in comparison to autosomes. Typically, MRRs occupy one end of a chromosome and FRRs start from the other end. Atlantic halibut has subtelomeric or acrocentric centromeres [23] and for all 18 chromosomes where we could infer centromere placement by mapping centromeric markers [20] to IMR_Hiphip.v1, the FRR coincided with the centromere (S3 Table). We next sought to determine whether meiotic recombination is sex-restricted also in species closely related to Atlantic halibut but could not find any publicly available pedigree datasets for other Righteye flounders. Since Atlantic halibut π was found to be higher for MRRs than FRRs, we decided to investigate whether π is correlated with MRR/FRR classification also in other Righteye flounder species. We could not find any publicly available genome sequencing data for Pacific halibut and instead used RNA-sequencing data to estimate π for this species. For Greenland halibut (*Reinhardtius hippoglossoides*) and European plaice (*Pleuronectes platessa*) we estimated π from publicly available RAD-seq- and whole genome shotgun sequencing data, respectively. The across-species comparison of π distribution in MRRs and FRRs revealed a striking agreement among the four species analyzed, with regions corresponding to Atlantic halibut MRRs having higher π (Fig 4A–4C), indicating that heterochiasmy has been conserved among righteye flounders.

We investigated whether MRRs and FRRs differed for repeat content and annotated repeats in IMR_Hiphip.v1 by means of RepeatMasker [30] using the *Actinopterygii* database. RepeatMasker raw output was parsed by repeat type and the density per type was investigated for each chromosome (S4 Table). Comparison of repeat type density in MRRs and FRRs revealed a markedly higher density of Short Interspersed Nucleotide Elements (SINEs) in FRRs than in MRRs while an opposite pattern was observed for simple- and low complexity repeats (Fig 4D). We also observed that the Long Interspersed Nucleotide Element (LINE) density increases towards the FRR ends of chromosomes, while simple repeats and low complexity repeats show the opposite pattern, being enriched towards the MRR end (S9 and S10 Figs).

### The insertion on chromosome Y is of transposon origin and contains a *gsdf* promoter

In an effort to identify candidate Structural Variants (SVs) between Y and X that may influence *gsdf* expression, we aligned all the ONT reads from the male individual used for genome assembly to the IMR_hipHip.v1 assembly and used the software Sniffles [31] to call SVs along the genome. This analysis revealed a large set of putative SVs (S5 Table), two of which coincided with the *gsdf* locus, both detected as heterozygous deletions in relation to the reference at chr13:8503901–8505128 (1,227 bp) and chr13:8509688–8509767 (79 bp). Manual inspection of these hits revealed that both ONT reads and linked read pseudo-haplotypes from the male assembly individual validate the assignments made by Sniffles. We used alignments of the male and female pool sequencing data and the phases of ONT long reads to conclude that the insertion allele at these two indels is present on chrY. The HiC data used for scaffolding the assembly was used to screen for large-scale SVs that may differ between X and Y. This analysis indicated that no large inversions are present on chr13 and that neither Y nor X are part of any large translocations with other chromosomes, as such events would appear as off-diagonal contacts in the contact map (S11 Fig).

The 1.2 kb insertion upstream of *gsdf* (INS1) is not found in the corresponding Pacific halibut locus, supporting that this region is derived on the Y chromosome in Atlantic halibut. INS1 (IMR_Hiphip.v1 chr13:8503902–8505127) is highly similar to 24 other loci in the genome assembly. Sequence alignments showed high sequence similarity starting with TG and ending with CA di-nucleotides in 19/24 loci. The remaining 5 loci showed strong, further extending similarities to each other, and we therefore extracted additional sequence flanking these five loci to perform a 7–8 kb alignment, which also terminated in conserved TG/CA di-nucleotides in four out of the five sequences. The TG/CA-ends are hallmarks of retrotransposon long terminal repeat (LTR) boundaries, and we could also identify 4–6 nucleotide sequence duplications immediately flanking the insertions, indicating target site duplications (TSDs) that confirm the transposable element (TE) insertions as retrotransposons. Although these TE hallmarks were obscured at the INS1 site upstream of gsdf, the sequence similarity and alignment to the related loci in the genome support its TE origin (S1 File). One of the five longer sequences (IMR_Hiphip.v1 chr14:3028070–3045206) was used as query to search the DFAM database [32]. This search revealed a 5254 nt long hit against the 5410 nt long *Danio rerio* Gypsy transposable element (Gypsy6-I_DR, Bit score 2342), supporting by sequence similarity association that INS1 was derived from a Gypsy retrotransposon that inserted in antisense orientation relative to *gsdf*. Strong similarities between the full-length Gypsy LTR sequences and INS1 revealed that INS1 is indeed an LTR remnant of an ancient Gypsy insertion. This association is further supported by RepeatMasker analysis of the five putative full-length hits partially matching INS1, as they all contained Gypsy sequence annotations.

**Fig 5:**
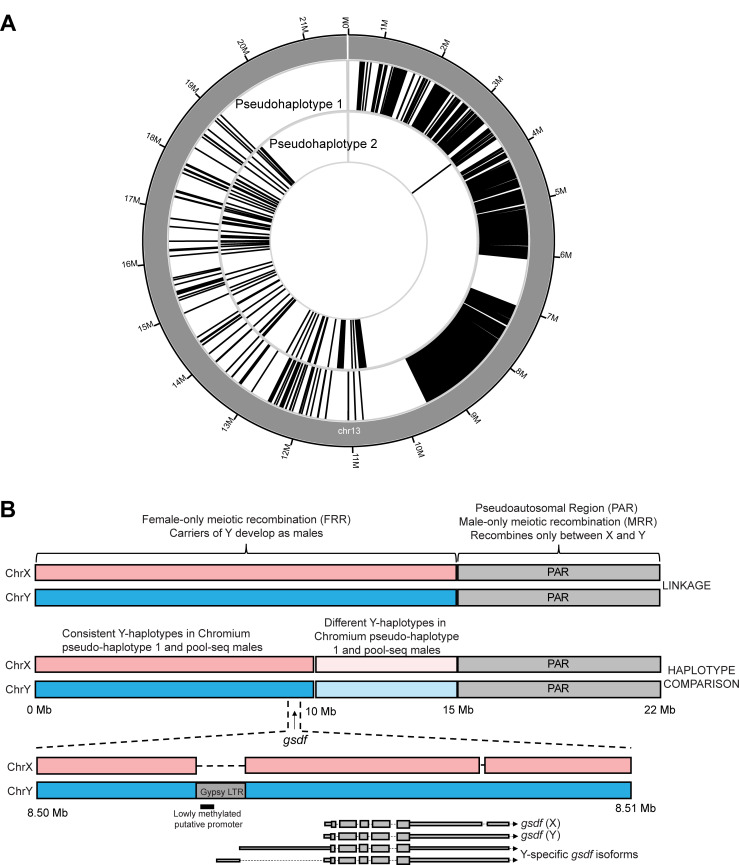
Chr. Y carries a transposon-derived LTR upstream of *gsdf*. **A** Agreement between reference individual Chromium pseudo-haplotype assemblies and chrY-markers from Pool-seq of males and females. Black lines show markers along chr13 where pseudo-haplotype 1 (outer track) and pseudo-haplotype 2 (inner track) are in agreement with the Y-allele defined from pool-seq. In the interval 0-10Mb pseudo-haplotype 1 shows a striking agreement with Y-markers defined from pool-seq data. **B** Schematic representation of the Atlantic Halibut sex chromosomes as inferred from linkage analysis alone and from comparison of Pool-seq defined Y-markers with Chromium pseudo-haplotypes of the assembly male. X and Y haplotypes differ for a 1.2 kb segment derived from a transposable element 2 kb upstream of the *gsdf* transcription start site. Males carry the insertion allele which contains a lowly methylated CpG-island inferred to act as a derived promoter of *gsdf* uniquely in males. The shorter *gsdf* isoforms were observed in Atlantic halibut adult testis in as well as adult ovary and testis of Pacific halibut. The two *gsdf* gene models labeled chr13Y-specific indicate isoforms detected only in males from three separate developmental stages. An IGV-plot of this region is presented in S12 Fig.

From the ONT data used for genome assembly we could assess DNA methylation at the *gsdf* locus. Interestingly, we observed low DNA methylation within INS1, in a region coinciding with a predicted CpG-island (Figs 5 and S12). Further inspection of the lowly methylated CpG-island revealed predicted Sp1 binding sites indicating that this locus may act as a promoter region for *gsdf*. It is clear from both ONT read alignment and the linked read pseudo-haplotype alignments that the chrY haplotype carries INS1. We developed a PCR assay to amplify into INS1 and observed that this assay gave clear bands of the expected size for 10 adult phenotypic males while no bands were observed for 10 phenotypic females (S13 Fig). Expression of embryonal *gsdf* (28–54 dpf) from the Y-allele (*gsdf*-Y) extends further upstream of the transcription start site than gene models both in adult Atlantic halibut testis and in male- and female reproductive tissues in adult Pacific halibut. The longer *gsdf-Y* isoforms contain 5’UTRs extending into the Gypsy LTR INS1 (Figs 5 and S12), which supports promoter activity of INS1.

### Analyses of Y decay and X/Y allelic imbalance

In addition to the ONT genome assembly, we also sequenced the same male using a Chromium Genome linked read library and used the resulting data to generate two separate pseudo-haplotype (PH1 and PH2) assemblies which successfully phased the first 9–10 Mb of chr13 into separate scaffolds for the X and the Y chromosome (Fig 5A). Loss of X/Y recombination is expected to lead to chrY decay, and we investigated whether we could find evidence of this by contrasting coding changes against Pacific halibut gene models for chrX (PH2) and chrY (PH1) haplotypes along the first 9 Mb of chr13. Numbers of non-synonymous substitutions for the interval corresponding to Atlantic halibut X/Y (chr13: 0–9 Mb) were 68 and 70 for PH1 and PH2, respectively, suggesting little if any decay of the Atlantic halibut chrY (S14 Fig). That no large-scale loss of genetic material has occurred on chrY is supported by the observation that PH1 and PH2 are both 20.4 Mb in size. It is clear that the overall divergence, as well as non-synonymous and synonymous divergence is higher between species than between Atlantic halibut X and. Y (S14 Fig), showing that the Atlantic halibut sex-chromosome evolved after the split from Pacific halibut, i.e. < 4.5 MYA. Altogether, at 7611 single nucleotide alleles Atlantic halibut chrY differs from another allele shared among Atlantic halibut chrX and Pacific halibut. The majority of these (excluding the 68 nonsynonymous sites, giving 7543 sites) are expected to be neutral. Using the 7543 putatively neutral sites together with mutation rates determined in Atlantic herring (2x10^-9^ per site per generation [33] and Cichlid fish (3.5x10^-9^ per site per generation [34] and a mean generation time of seven years, we estimate that the age of the ~10Mb Atlantic halibut chrY-locus is at most 1.5–2.6 million years. This calculation should be interpreted as the absolute upper possible age and is likely a gross overestimation since Atlantic halibut π dictates that most of the 7543 observed differences could represent standing variation present at the time of the X/Y split, rather than mutations which occurred after the split. From the intersected RNA-seq alleles on chr13 (S4 Fig), we assessed allelic imbalance for 130 SNPs in the 7 male RNA-seq samples and six of these SNPs showed consistent allelic imbalance, three expressed in higher levels from X and three in higher levels from Y, including *gsdf* (S15 Fig).

## Discussion

In this study we show that *gsdf* is most likely the master sex determining gene in Atlantic halibut. The same conclusion was recently made in a separate study [35]. In comparison with the study by Einfeldt et. al, we provide much more detail regarding the molecular events accompanying the birth of *gsdf* as a novel sex determining gene. It should be noted that the Einfeldt et al. study has a different chromosome/LG numbering compared to our and previous studies [19,20] (S6 Table). The germline is constantly being invaded by transposable elements and sometimes such insertions will result in host phenotypic change, which may be favored by selection [36]. Our interpretation is that following an ancient Gypsy element insertion on the autosomal progenitor of chr13, this element degraded and left a remnant LTR, which now acts as a promoter driving the expression of *gsdf* ectopically during development of XY individuals, making carriers develop into phenotypic males. Interestingly, in Luzon ricefish, where *gsdf* is also the MSD gene, the region immediately upstream of *gsdf* was shown to be most important for modulating *gsdf* expression [24]. The region tested corresponds approximately to the site of TE insertion in the Atlantic halibut *gsdf* locus. A role of *gsdf* in early gonadal development is supported by experiments in medaka where targeted homozygous deletion of *gsdf* in genetic (XY) males triggers an early development of ovaries, with the majority of these *gsdf* KO homozygote fish still developing testes later in life [37]. Furthermore, in accordance with previous studies [38] we observe *gsdf* expression in adult testes and ovaries in Pacific halibut and in Atlantic halibut testis, indicating a function of Gsdf in mature gonads. Little is known about when sex differentiation takes place in halibut, but in this study we observe early increasing expression (48 hpf until 13 dpf) of *gsdf* in developing males but not females, presumably due to expression of *gsdf* prior to and during early testis development. Our analysis was restricted to whole embryos and further studies will be needed to show whether *gsdf* expression is restricted to early testicular differential stages and investigate the effects of the chrY specific Gypsy element insertion on *gsdf* expression. TEs are known to be involved in sex-chromosome evolution [39], e.g. TEs are probably responsible for translocation of *sdY* in salmonids [40] and in Medaka a LINE insertion at the *dmrt1b* locus has been implicated as the sex-determining variant [41]. It has been repeatedly observed that TEs are repurposed for genomic innovation by domestication [42], as might be expected for a dynamic part of the genome.

In this study we observed that meiotic recombination is dramatically different between male and female gonads, and this can also be seen when revisiting a previous Atlantic halibut linkage study [20], where this phenomenon was not strongly emphasized. Several hypotheses exist but little is known about which mechanisms contribute to the establishment and maintenance of heterochiasmy [43]. A common feature for observations of heterochiasmy in teleosts is enrichment of male crossovers towards chromosome ends [14,16,44,45], which is also the case in Atlantic halibut. All chromosomes for which we could annotate centromere placement had the centromeric end coinciding with the FRR (S3 Table), which supports findings from other teleost species, where female meiotic recombination is directed towards the centromere [14,16,44,45]. The observation that male and female crossovers predominantly occurred in mutually exclusive regions of chromosomes was more surprising since previously described heterochiasmic species tend to have more evenly distributed female recombination along the chromosomes [13,14,45]. We found that four righteye flounder species all had higher π in the regions orthologous to Atlantic halibut MRRs than in corresponding regions orthologous to FRRs (Fig 4C). This may reflect conservation of regions of sex-restricted recombination and heterochiasmy pattern across *Pleuronectidae*. The reason for this interpretation is that few polymorphic sites are expected to be shared between species over the millions of years separating the four analyzed species. Thus, the diverging π distributions must have been established independently, presumably by selective forces associated with heterochiasmy. It should be noted that our hypothesis of shared heterochiasmy among *Pleuronectidae* is solely based on genomic distributions of the proxy π and that heterochiasmy should be validated by analysis of meiotic recombination also in other species than Atlantic halibut.

In medaka, sex-restricted recombination between sex chromosomes has been shown to follow the XY male pattern also in female-to-male sex-reversed XX fish [46], which supports that a mechanism associated with the gonadal environment governs recombination suppression in meiosis. We propose to study the mechanisms of heterochiasmy in sex-reversed genetic female Atlantic halibut, which are used as breeding males in aquaculture. Regions of TE-accumulation in genomes have been implicated as putative heterochromatin sinks [47], and both TEs and heterochromatin are positively correlated with suppressed recombination [48]. It is difficult to disentangle whether TEs are involved in the establishment of meiotic recombination suppression or if their differing distributions in MRRs/FRRs is merely a consequence of how the genome evolves due to unknown selective pressures imposed by heterochiasmy already being in place. One possibility could be that Atlantic halibut male and female gonads differ for which regions of the genome become heterochromatin, perhaps as a defense against TE transposition, and that male and female gonads direct recombination differently in response to variable TE content along the chromosomes. Possibly, transposable elements are more likely to accumulate in FRRs, for example if regions only recombining in males (MRRs) exist in a state less accessible for transposition during female and/or male meiotic recombination. Accumulation of local TE clusters in FRRs may also contribute to the further homogenization of this chromosome compartment by gene conversion of homologous TE insertions. It should be noted that we have analyzed repeats broadly, and future studies aiming to investigate repeat classifications in relation to heterochiasmy may benefit from more detailed classifications. We show that π is higher in MRRs than in FRRs and hypothesize that the higher recombination rate on the physically smaller MRRs increase the probability of retaining diversity at linked sites during purifying selection [49], or that differences in purifying selection due to the differing TE-composition landscapes of FRR and MRRs has been involved in shaping π.

Sex-restricted meiotic recombination, as we describe here, suppresses recombination for large segments along autosomes. Along the lines of previous studies [13,15,16] we propose that heterochiasmy has the potential to facilitate the evolution of haplotypes on chromosomes carrying new SDGs by linking sexually antagonistic (SA) alleles to the SDG (S16 Fig), and we hypothesize that heterochiasmy may be associated with accelerated sex chromosomsome turnover cycles [50] and speciation rates [45,51]. Interestingly, in two Hyla tree frog species, the sex determining gene in a Z/W system is located in a FRR region [13], which disagrees with our proposed model (S16 Fig). The existence of such a FRR Z/W system suggests that a single sex determining locus, without linked sexually antagonistic (SA) loci, is sufficient to uphold a genetic sex determination system and therefore challenges the importance of SA genes. Interestingly, we observed a 1 Mb segment (chr13:5.5–6.5 Mb) (Fig 1B) showing differentiation equal to the genome average, which we interpret as X/Y recombination, although rare, not being fully suppressed on chrY (the FRR of chr13). This could mean that the non-recombining and male-promoting core of chrY is smaller than we could define in this study. Previous fine mapping of the sex determining locus to a smaller marker interval [19] (Fig 3A) in a region corresponding to the herein defined chrY supports that recombination is not fully suppressed between X and Y.

For mammalian Y, as well as avian W, loss of purifying selection over millions of years has led to pseudogenization, gradual loss of genes and a reduction in chromosome size [52], where remaining genes and regulatory elements are involved in promotion of sex-specific phenotypes. Our analysis of coding changes observed on linked read pseudo-haplotypes corresponding to chrY and chrX revealed no enrichment of missense mutations on chrY, suggesting little if any chrY decay. A limitation of our study with regard to studying chrY decay is the haploid nature of the IMR-Hiphip.v1 assembly. As expected, X and Y haplotypes on chr13, like haplotypes on all autosomes, were not sufficiently divergent for the assembler to output separate contigs for X and Y. Future efforts, using for example highly accurate PacBio HiFi sequencing and haplotype-resolved scaffolding, should be able to assemble X and Y into separate scaffolds which could be used for assembling these relatively lowly differentiated chromosomes separately. Such an endeavor, coupled with larger sample sizes of phenotypic male and female fish will be beneficial for fine-scale investigation of differences between X and Y including analyses of chrY decay.

It has been reported that female is the heterogametic sex (ZZ/ZW) in both Pacific halibut and European plaice [17] as well as the half-smooth tongue sole [53], with the main candidate MSD gene being *dmrt1*. This makes it conceivable that the Atlantic halibut X/Y system replaced a Z/W system shared across different flatfish species. We conclude that a retrotransposon insertion into the *gsdf* locus started the transformation of an autosome into a sex chromosome in Atlantic halibut less than 4 million years ago.

## Methods

### Ethics statement

For this study the halibut were reared under conditions similar to standard commercial fish farming conditions. Such conditions are listed as an exception in The Norwegian Regulation on Animal Experimentation, thus approval of the experimental protocol of this experiment by NARA (the governmental Norwegian Animal Research Authority) was not required.

### Halibut material

All samples were taken during routine production of Atlantic halibut at the Austevoll Research Station, Institute of Marine Research, Storebø, Norway. Halibut breeding individuals were stripped, eggs fertilized and incubated, and juvenile halibut produced according to standard aquaculture protocols developed at the Austevoll Research Station [54–56]. Briefly, females were stripped according to their individual ovulatory rhythms to ensure high (>90%) fertilization. Fertilized eggs were incubated in 250 l conical incubators with upwelling water for 60 day degrees (i.e. 10 days at 6°C) and transferred to larger incubation units, silos (vol. 50,000 l) before hatching. Yolk sac larvae were held in the silos until ca. 265 day degrees posthatch when they were moved to first feeding tanks and fed *Artemia* for 70 days until metamorphosis was complete. At this stage, the larvae were weaned onto a formulated diet. Juvenile halibut were moved to ongrowing tanks when they had reached an average size of ca. 10 cm and held there until they had reached a harvest size of 2–5 kg. One adult, three-year old male was euthanized with metacain (100 mg/l) and muscle, spleen and blood samples were taken for reference genome sequencing. For sequencing of pooled family samples, tissue samples were taken from ten full siblings of mixed sex (ten males and ten females). All samples were snap frozen in liquid nitrogen (N_2_) and stored at -80°C before DNA extraction. RNA-sequencing was made on a series of samples taken through embryonic development. Before hatching, embryo samples were snap frozen in liquid N_2_ and stored at -80°C. Larvae were euthanized in metacain (100 mg/l), transferred into RNA later, kept at 4°C for 24 h and stored at -20°C until RNA isolation. The samples for qPCR were snap frozen in liquid N_2_ and stored at -80°C. Digital images of all embryo-larval stages were captured using video cameras Moticam 1080 (Motic) mounted on Olympus SZX10 stereomicroscope using Motic Live Imaging Module. Living embryos and larvae were individually immobilized in a Petri dish containing 2% methylcellylose/98% seawater. Images were then scaled and processed by ImageJ software [57].

### DNA extraction and sequencing of male and female pools

DNA was extracted from muscle samples using Qiagen DNeasy Blood and Tissue Kit. Paired-end libraries of equimolar male (10 individuals) and female (10 individuals) DNA pools and from one single individual of each sex were constructed using the Genomic DNA Sample Preparation Kit (Illumina) according to manufacturer’s instructions and sequenced on the Illumina HiSeq2000 platform (Illumina) at the Norwegian Sequencing center (https://www.sequencing.uio.no, Oslo, Norway).

### High Molecular Weight DNA extraction and sequencing

Several tissues were collected from a single male Atlantic Halibut. Muscle and spleen were snap frozen in liquid nitrogen and stored at -80°C until further use. Fresh blood was used to isolate erythrocyte nuclei and then prepare High Molecular Weight (HMW) DNA from these nuclei according to a Phenol/Chloroform protocol (Quick). Extracted DNA was either left unfragmented or sheared using either MegaRuptor (Diagenode) or by passing DNA through a 100μl pipette tip 10–20 times. From these DNA samples we produced MinION sequencing libraries with the standard adapter ligation protocol kit (LSK-108), 1D^2 sequencing kit (LSK-308) and the RAD002 and RAD003 kits (Oxford Nanopore Technologies). For libraries prepared with the RAD002 and RAD003 kits we used a protocol developed to produce ultra–long reads (Quick, protocols.io). In total we used 9 flow cells and sequenced 18.8 Gbp in 2,963,108 reads with a read N50 of 17.8 kb. Sequenced fast5 raw signal data files were basecalled using albacore v.2. The size distribution of sequenced reads is presented in S1A Fig. We also used the collected muscle samples to produce HiC libraries (Dovetail), sequenced on one lane of HiSeq X (Illumina). One of the DNA preps inferred to have HMW DNA was used to prepare a linked reads Chromium library (10x genomics) and was sequenced on one lane of HiSeqX (Illumina).

### Genome assembly, polishing and scaffolding

Nanopore reads passing filters were used in assembly by first constructing all-vs-all alignments between all reads using minimap2 [58]. These all-vs-all alignments were then used in the software miniasm to produce a haploid contig assembly. The contig assembly was polished using racon [59] with the nanopore data followed by pilon [60] using the Illumina data from the male pool. Scaffolding of the polished miniasm assembly was performed using the HiRise pipeline (Dovetail Genomics). The quality of the generated assembly was assessed using BUSCO [61]. Initial nanopore contigs assembled with miniasm [58] had a contig N50 of 3.2Mb and the resulting final assembly had a scaffold N50 of 27.2 Mb.

### Assembly of linked read data

The software Supernova2 [62] (10X Genomics) was used to produce a genome assembly of the sequencing data resulting from the Illumina sequencing of the Chromium library. For this assembly, reads were subsampled to get close to the recommended 56x genome coverage recommended in the Supernova2 user manual. The two pseudo-haplotype linked read assemblies featured one scaffold each which covered the whole of IMR_Hiphip.v1 chr13. We used SNP markers identified as divergent between the male and female short read data to investigate the pseudo-haplotypes in relation to sex divergent markers, i.e. if one pseudo-haplotype could represent the Y chromosome and the other the X-chromosome. This analysis revealed that for the first 10 Mb of chr13, the allele being heterozygous in males and not present in females (the perceived Y-allele) was observed on pseudo-haplotype number 1 (1,954 SNPs). The perceived Y-allele was observed only for 2 SNPs in this region for pseudo-haplotype number 2. The corresponding counts for the rest of chr13 was 125 and 213 alleles on pseudo-haplotype numbers 1 and 2, respectively.

### RNA-sequencing and gene annotation

Total RNA was extracted from whole larvae from seven different stages of development using miRNeasy Mini Kit (Qiagen) and treated with TURBO DNase-Free kit (Ambion). TruSeq cDNA libraries were sequenced on the HiSeq 4000 sequencing platform (Illumina). The average number of reads was 50,320,639 per sample. Twenty-two samples (four samples from 54 dpf and three samples from each other stage) were assembled individually with Trinity using the Agalma pipeline [63]. The transcriptome assemblies were annotated with BLAST against Swissprot (download date 20191227) and the Ensembl gene models of zebrafish (GRCz11) and stickleback (BROAD S1). The hits with the best scores were kept. Transcripts from all assemblies were mapped to the halibut genome with BLAT. The thresholds for mapping were set to 98% match for sequences with length > = 401 nt and 95% match for sequences with length < = 400 nt. Long sequences were mapped before short sequences. The mapped sequences were clustered on genome location, selecting the sequence with the highest score for each non-overlapping cluster. Only clusters with an annotation (>300 in BLAST score threshold) were saved. Neighboring clusters annotated with the same gene from either Swissprot, zebrafish or stickleback were merged in cases where the annotation indicated that the gene was split in several clusters (the neighboring clusters were only merged in cases where the annotation was from different parts of the same gene). For the merged clusters, the sequence with the highest BLAT score from all merged clusters were chosen. Additionally, the transcriptome assembly sequence with the highest scoring BLAT match to the merged cluster was added. We found 15,273 annotated genes based on the Trinity assemblies. The zebrafish and stickleback ENSEMBL gene models were mapped to the halibut genome using exonerate in order to add genes without RNA-seq evidence. The exonerate hits were clustered by location, using the exonerate hit with the highest score for each cluster. Sequences for each exonerate based gene were extracted from the halibut genome sequence. 2,269 genes were added based on the exonerate analysis. We added 222 genes that had no annotation (BLAST threshold score <300) but had RNA-seq expression. After running BUSCO 3.1 with the Actinopterygii odb9 dataset on the non-filtered Trinity assemblies that mapped to the genome [61], we added 550 genes from the BUSCO analysis that were missing from our filtered list. This resulted in a total number of 18,314 genes and 20,579 transcripts giving a 80.9% complete, 8.7% fragmented and 10.4% missing BUSCO coverage.

### Nanopore sequencing of testis RNA

Testis tissue was homogenized at 4°C in 1ml QIAzol Lysis Reagent (Qiagen, Hilden, Germany) together with RNase-Free Zirconium Oxide Beads (NextAdvance, Inc., Troy, NY, USA) during 1 min, maximum effect, in a BulletBlender (NextAdvance) and total RNA was prepared according to the QIAzol (Qiagen) protocol. Polyadenylated RNA was isolated with the Dynabeads mRNA direct purification kit (Thermo Fisher Scientific), following the kit protocol. For full-length cDNA Oxford Nanopore sequencing, the cDNA-PCR Sequencing SQK-PCS108 kit (Oxford Nanopore Technologies) was used according to the kit protocol with minor modifications. The finished cDNA library was sequenced on a MinION device using a R9.4 flow cell (Oxford Nanopore Technologies).

### Screening for male vs. female divergence

Illumina sequences from the male- and female DNA pools were filtered by trimmomatic [64], then aligned to the assembly IMR_hipHip.v1 using BWA-MEM [65]. Aligned reads were subjected to duplicate flagging (“Picard Toolkit.” 2019. Broad Institute, GitHub Repository. http://broadinstitute.github.io/picard/; Broad Institute) and were then used as input for calling SNPs as well as small Insertions/Deletions using Genome Analysis Toolkit (GATK) [66] UnifiedGenotyper. Raw variant vcf files were subjected to variant filtration using GATK Best Practices procedure with SNP-clusters being added as an additional filter (more than 5 SNPs in a window of 20 bases were filtered out). SNPs passing filters (n = 2,500,656) were isolated from the vcf file and allele frequencies from the male- and female pools were extracted and used for calculating male vs. female ΔAF, first calculated for each individual SNP having read depths > = 10, and average ΔAF was then calculated for 500kb windows along each chromosome.

### Sex differentiating PCR assay

Genetic sex of each embryo was determined by PCR using sex-specific primers (Table 1). The thermal cycling conditions were as follows: 94°C for 5 min, 32 cycles with 94°C for 30 sec, 65°C for 1 min and 72°C for 1 min, and finally 72°C for 5 min. Each 12 μl PCR reaction contained 5X GOTaq Flexi Buffer, MgCl, dNTPs, GoTaq Polymerase, PCR primers and template cDNA.

**Table 1:** Brx PCR primers.

PCR primer	5’-3’ sequence
brx_fwd	CAAGCCTGTTCACATCGAAA
brx_malerev	ATGGCAACAAACGCTTATCAA
brx_femalerev	TGGCAACAAACGCTTAACAC

### Differential gene expression between sexes

We generated RNA-sequencing data from 12 Atlantic halibut whole embryos representing stages 28 (n = 4), 43 (n = 4) and 54 (n = 4) dpf. Since one cannot determine the phenotypic sex at this stage we aligned the RNA-seq data using Bowtie 2 [67] to IMR_hipHip.v1 and extracted expressed allele counts at positions identified as divergent between males and females in the pool DNA-seq. Extracted RNA-seq allele ratios were used to cluster the individuals, revealing two well-separated clusters of XY and XX individuals and this assignment was used to identify genetic male and female individuals for the differential expression screen. The differential expression screen involved contrasting across-sample normalized gene expression levels of the seven inferred males to the five inferred females in a two-tailed t-test. Principal Component Analysis (PCA) was performed, using the R function “prcomp”, on the normalized gene expression levels from the 12 embryos.

### *gsdf* quantitative PCR

Genomic DNA and total RNA was extracted from single embryos using the Allprep DNA/RNA/miRNA Universal Kit (Qiagen), according to the manufacturer’s instructions. The RNA was DNase treated as part of the extraction process, according to the manufacturer’s instructions. For each sample, cDNA was synthesized from 125 ng RNA using the Superscript VILO cDNA synthesis Kit (ThermoFisher Scientific). The primers used for qPCR were designed using BatchPrimer3 (https://wheat.pw.usda.gov/demos/BatchPrimer3/) (Table 2). The gene *gtf3c6* was used as an internal reference gene for normalization of the qPCR data due to its stable expression in the developmental stages analysed in this study (S17 Fig). Using different RNA concentrations, the PCR efficiencies (E = -1+10(-1/slope)) for the primers were calculated to be 108.5% (*gsdf*) and 92.4% (*gtf3c6*). A melt curve analysis was performed to confirm that the primers did not amplify more than one PCR product. qPCR was performed in duplicates in 384-well optical plates in a QuantStudio 5 Real-Time PCR system (ThermoFisher Scientific) using default settings. 2 μl cDNA (diluted 1:20) was used in a 6 μl Fast SYBR Green qPCR reaction (ThermoFisher Scientific). No-template controls for each gene were run in all qPCR plates. The relative gene expression level was calculated using the comparative Ct (or 2^−ΔΔCt^) method. All values were normalized to *gtf3c6* and calibrated to the sample with the lowest ΔCt.

**Table 2:** Primers used for qPCR.

QPCR primer	5’-3’ sequence
*gsdf* forward	TGCTGGACTCGTCCACATAG
*gsdf* reverse	GTCCATCATCCCACACCAAT
*gtf3c6* forward	CACCGTGAAGAAGCTGATGA
*gtf3c6* reverse	ACTTCCTGCTGTGCTGGTTT

### Detection of structural variants, small insertion/deletions and annotation of repeat sequences

The long-read SV identification tool Sniffles [31] was used to identify SVs from the ONT data. The ONT data from the single male individual used for genome assembly was aligned to IMR_hipHip.v1 using minimap2 [58] with the “-x map-ont” option and the resulting bam file was used in sniffles, requiring > = 5 observations of a variant allele to be retained as a potential SV.

### Analysis of the evolutionary origin of the 1.2 kb insertion upstream of *gsdf*

The 1.2 kb insertion (IMR_Hiphip.v1 chr13:8503902–8505127) was used as a query sequence in BLAT [68] searches against IMR_HipHip.v1. Full length hits were retained (n = 30) and were extended to include 2kb up- and downstream sequences and these approximately 5.3 kb sequences were used as input for multiple sequence alignment using MUSCLE [69]. The resulting alignments were visualized in AliView [70] and abrupt drops in sequence similarities were observed for 24 of the sequences on each flank. The extent of highly similar sequences started and ended at perfectly conserved TG and CA di-nucleotides, the hallmark motifs of LTR boundaries, and the insertion was immediately flanked by 4–6 nucleotide duplications of loci TE target sites (TSD) confirming the LTR-retrotransposon insertion.

### DNA methylation calling from ONT data and CpG island prediction

Basecalled ONT reads previously used for the genome assembly were aligned back to the IMR_Hiphip.v1 assembly using minimap2. The software nanopolish [71] was used to index the raw ONT fast5-files to enable extraction of likelihoods of CpG site methylation along individual mapped reads in genome assembly coordinates. Next, the nanopolish script “calculate_methylation_frequency.py” was used to extract the methylation frequency at each adequately covered CpG cluster in the assembly. CpG islands were predicted for the IMR_Hiphip.v1 assembly using gcluster [72] employing default settings.

### Estimation of nucleotide diversity

For Atlantic Halibut we estimated π by creating chromosome-specific samtools mpileup files from the aligned data previously used for variant calling, using only positions with a mapping quality > = 20 and read depths ranging between 10 and 50. The script “WinNuclDivmPileup.py” (https://github.com/cjrubinlab/python_scripts2) was used to estimate π in 500kb windows along the genome for the male- and female pools. For Pacific halibut no DNA-sequencing data was available in public sequence repositories, so we devised a way to estimate π from RNA-sequencing data. Sequencing data from 12 RNA-seq experiments of various tissues and individuals was downloaded from the SRA (SRR11826178-SRR11826189) and these data were aligned to the Oxford nanopore derived Pacific Halibut genome assembly (GCF_013339905.1) using the STAR aligner [73]. The resulting alignments were randomly subsampled 20 times and then subjected to duplicate-pair removal using picard-tools MarkDuplicates, retaining 70% of the reads after filtering. From the bam file containing filtered reads an mpileup file was generated using samtools (mapping quality exclusion filter < 20). For the Greenland Halibut (*Reinhandtius Hippoglossoides*) we downloaded the genome assembly from NCBI (GCA_006182925.2_MU_Rhippo_v1) as well as RAD-sequencing data from 20 individuals (SRA accessions: SRR11351808-SRR11351827)[74], aligned the short-read data to the assembly using minimap2 with the “-x sr” option. From mpileup files derived from samtools mpileup for Pacific Halibut and Greenland halibut the estimated heterozygosity (He) was calculated for positions having coverage> = 10 and mapping quality > = 20 using the WinNuclDivmPileup.py” script. For European plaice (*Pleuronectes platessa*), a species lacking a genome assembly, we downloaded paired genome resequencing Illumina reads (SRA accession: SRX8240416), aligned these reads to the IMR_Hiphip.v1 assembly using BWA-MEM and proceeded with the same pipeline as was used for estimation of Atlantic halibut π. In order to lift over genomic intervals annotated as MRRs and FRRs in the Atlantic halibut to the Pacific- and Greenland halibut assemblies we used the satsuma [75] commands “Chromosemble” to generate a detailed list of lift-over intervals and “BlockDisplaySatsuma” to summarize the detailed list into coherent blocks. We then converted these coordinates to the bed-format and used the bedtools [76] “intersectbed” command to link coordinates in the other assemblies to the corresponding coordinates in the Atlantic halibut assembly and MRRs and FRR classification.

### Linkage analysis

RAD-sequencing data from an Atlantic halibut half-sib pedigree altogether comprising three parents and 90 offspring were downloaded from the SRA (accession = SRX193134) [19]. The downloaded individual files were split by barcodes and were then connected with annotation information from the original publication regarding phenotypic sex, parent/offspring status and family B/C [19]. The paired-end data from each individual in the pedigree was then aligned to IMR_hipHip.v1 using BWA-MEM. Resulting bam-files were subjected to duplicate removal using picard-tools “MarkDuplicates”, then raw variant (SNP and indel) calling using GATK UnifiedGenotyper. Raw SNPs were filtered using GATK “VariantFiltration” with the following arguments, also with a list of raw indel positions given to the “InDel” mask; “—maskName InDel”, “—maskExtension = 5”, “—clusterSize = 5”, “—clusterWindowSize = 15”, "BaseQRankSum<-7.0", "BaseQRankSum > 7.0", "MQRankSum < -7.0", "MQRankSum > 7.0", "QD < 5.0","FS > 50.0","ReadPosRankSum > 7.0", "ReadPosRankSum < -7.0" and "DP > 40". SNPs remaining after filtration were saved in a new vcf file which was given as input to Lep-MAP3 [77] to generate recombination maps for the two families. The commands used in Lep-MAP were: “ParentCall2”, with the options (removeNonInformative = 1 XLimit = 2 halfSibs = 1), then “Filtering2”, with the option data = - (dataTolerance = 0.01), then “SeparateChromosomes2” with the options (lodLimit = 10 distortionLod = 1) to identify linkage groups in the pedigrees. SeparateChromosomes2 with lodLimit = 10 generated a map of 31 linkage groups, out of which 20 had 964–3063 markers assigned to them (24 were expected based on karyotype). We continued with the output from SeparateChromosomes2 (lodLimit = 10) to run “OrderMarkers2” to, for each identified linkage group, order the markers without having given the software access to genome assembly coordinates. The output files from the first iteration of “OrderMarkers2” were then subjected to a second round of “OrderMarkers2”, this time giving the Lep-MAP3 access to genome coordinates of markers on each linkage group using the option “evaluateOrder”. The resulting linkage group specific files containing male- and female centiMorgan (cM) positions of each marker were filtered to prune obviously inflated cM positions in the very beginning and the very end of assembled chromosomes and by splitting four linkage groups where two assembled chromosomes had been merged into one linkage group, thereby increasing the observed linkage group count from 20 to the expected 24. Resulting maps from this stage were retained for downstream analyses.

### Analysis of chrY decay

The two linked-read pseudo-haplotype assemblies (PH1 and PH2) of the male Atlantic halibut used for the genome assembly were aligned against the nanopore assembly and it was concluded that scaffold 45 from the two pseudo-haplotypes corresponded to chr13. Scaffold 45 from PH1 and PH2 were independently converted to overlapping short reads which were separately aligned using BWA-MEM to the Pacific halibut reference genome. SNPs and small indels were called using bcftools and these were annotated using SNPEff, with gene models derived from the Pacific halibut IPHC_HiSten_1.0 gtf-file “GCF_013339905.1_IPHC_HiSten_1.0_genomic.gtf”. SNPEff “missense” and “synonymous” annotations were counted for SNPs that were uniquely observed in one pseudo-haplotype Annotation frequencies were compared between the two pseudohaplotypes for the interval where they represent the X and Y (Atlantic halibut: chr13:0–9 Mb, Pacific halibut: CM023513.1:11–21 Mb).

### Pacific halibut RNA-seq contrasts

Sequencing data from 12 RNA-seq experiments of various tissues and individuals (SRR11826178-SRR11826189) was downloaded from SRA. Four of these tissues (testis, ovary, white muscle and brain) were used for characterization of *gsdf* gene expression levels and validation of gene models from the gff-file accompanying the Pacific halibut assembly on NCBI. Alignments were performed using STAR as described above for π estimation.

### RepeatMasker analysis

RepeatMasker v4.1.0 [30] was used to annotate both simple and interspersed repeats. We deployed the RMBLASTN (v2.6.0.+) search engine to scan for known repeats among ray-finned fishes (“-species actinopterygii”) using a database that combined Dfam_3.1 and RepBase-20181026 repeat sequences. A custom Perl script was used to parse the main output table from RepeatMasker and compute the densities of major repeat classes (i.e. “Simple_repeat”, “LINE”, “LTR”, “Low_complexity”, “DNA”, “RC”, “SINE”, “Satellite”, “Unknown”, “Retroposon”) in windows of 50 kbp and 500 kbp, respectively.

## Supporting information

S1 Fig **A** Read length distribution of nanopore reads used to generate the IMR_Hiphip.v1 assembly. **B** Assembly continuities for the initial ONT contig assembly and the scaffolded IMR_Hiphip.v1 assembly. **C** Dot plot of genome to genome alignment between the IMR_hiphip.v1 assembly and the Pacific halibut reference genome assembly (IPHC_HiSten_1.0). Five chromosomes show larger inversions which are visible as green alignments perpendicular to the linear alignments (blue line) in the plot. A high degree of synteny was observed.
(PDF)

S2 Fig HiC contact matrix after scaffolding the Oxford Nanopore contig assembly using HiRise. Scaffolding resulted in 24 major scaffolds, in agreement with the karyotype of Atlantic halibut. Chromosomes are sorted numerically 1–24 and are separated by white lines.
(PDF)

S3 Fig Principal component analysis of the RNA-seq data reveals clustering by maturation stage rather than genetic sex. Dots indicate coordinates of individual RNAseq samples on PC1 and PC2. Sample sames indicate their ages in days post fertilization (dpf). Box colors indicate the genetic sex assignment of samples.
(PDF)

S4 Fig **A** RNA-seq data samples clustered for 383 SNPs on chr13 previously found to be fixed in female DNA pool-seq while being variable in the male DNA pool-seq. We retained only positions were both alleles were observed in the RNA-seq data. Coordinates of genes along the Hiphip.v1 assembly are shown to the right. The samples without (NEG) *gsdf* expression in the RNA-seq and the samples with *gsdf* expression (POS) are in separate clusters. It was concluded that the individuals labeled XX and XY were female and male samples, respectively. **B** 65 SNPs fixed in the RNA-seq NEG samples but with both alleles observed in the RNA-seq POS samples along chr13. The nucleotide positions (n) of the SNPs are shown to the right of the Figure. Made in https://software.broadinstitute.org/morpheus/.
(PDF)

S5 Fig Genetic sex assignment by PCR. Sex differentiating PCR using sex specific primers (Table 1 in the manuscript) on cDNA from Atlantic halibut embryos at different developmental stages. Control samples were genomic DNA from individuals with known sex. Genetic sex of the samples was determined using a PCR based assay designed to differentiate genetic female and males. The forward primer was common for chr13X and chr13Y, while the reverse primers differed for two nucleotides in the 3’UTR of brx on chr13:9125004–9125007. For each individual sample (marked with a number) both female specific primers (brx_fwd + brx_femalerev; left well) and male specific primers (brx_fw + brx_malerev; right well) were run. In the case of a female sample, one band is detected in the left well while no band is detected in the right well. In the case of a male sample, two bands are detected. Genetic sex could not be determined for the 1, 8 and 24 hpf samples based on cDNA since this is prior to zygotic transcription of brx and the amount of gDNA extracted from these early stages were insufficient for reliable PCR.
(PDF)

S6 Fig Synteny of chromosomal regions associated with *gsdf*. The diagram is not to scale for the lengths of each gene nor the distance between genes. NCBI (http://www.ncbi.nlm.nih.gov) and Ensembl (http://www.ensembl.org) genome browsers were used to ascertain order, identity and orientation of genes immediately adjacent to *gsdf*.
(PDF)

S7 Fig **A** XY-scatter plot of the cumulative genetic distances of chromosomally anchored linkage map markers in males and females reveals a large extent of heterochiasmy. **B** MRRs are smaller but have higher recombination rate. Differences in effective recombination rate (cM/Mb) between the Male-only and Female-only meiotic Recombination regions (MRRs/FRRs) as a function of the fraction of chromosomal size annotated as MRR. Shown on the X-axis is MRR size divided by chromosome length. Shown on the Y-axis is the recombination rate (cM/Mb) for each MRR divided by the rate for the corresponding FRR. Circle sizes are proportional to chromosome lengths. The Atlantic halibut sex chromosome (chr13) is indicated by an arrow. Chr9, the smallest chromosome, is the only chromosome having a larger MRR than FRR as well as a higher female effective recombination rate.
(PDF)

S8 Fig The relationship between nucleotide diversity and recombination rate for MRRs and FRRs. On the x-axis: the log2 fold change (M-value) for recombination rate observed in MRRs and FRRs. On the y-axis: the log2 fold change (M-value) for nucleotide diversity in MRRs and FRRs. Circle colors indicate the chromosome and circle sizes are proportional to the relative size of the MRR on each chromosome (size MRR/ chr size). Chr13 (the sex chromosome) shows the largest difference of all chromosomes for MRR/FRR nucleotide diversity M-value. The FRR of chr13 is the X/Y chromosome.
(PDF)

S9 Fig Circular representation of repeat class content in the Atlantic halibut genome. The outmost track visualizes the individual chromosomes. Locations of MRRs and FRRs are shown as alternating black/grey rectangular tracks beneath each chromosome. Densities of repeat superclasses in 500 kb windows are indicated as dot plots in the five innermost tracks (LINE and SINE represent Long- and short interspersed nucleotide elements, respectively. LTR indicates Long Terminal Repeats. LCR and SR indicate low complexity- and simple repeats, respectively).
(PDF)

S10 Fig Density of RepeatMasker classifications along Atlantic halibut chromosomes. X-axes show coordinates along the chromosome (10^7^ bp). Y-axes show the proportion of nucleotides in 500 kb windows overlapping an element belonging to each specific repeat superclass. Repeat superclass is indicated to the left of each subplot. Circle colors indicate whether a 500 kb window is classified as a MRR or FRR.
(PDF)

S11 Fig HiC contact maps (all chromosomes and chr13 separately) of the male (XY) Atlantic halibut used to generate the genome assembly. No apparent large-scale inversions or translocations were observed (would appear as off-diagonal contacts)
(PDF)

S12 Fig Chr. Y carries a transposon-derived LTR upstream of gsdf. Male- and female haplotypes differ for a 1.2 kb transposable element derived segment 2 kb upstream of *gsdf* transcription start site of. Males carry the insertion allele which contains a lowly methylated CpG-island inferred to act as a derived promoter of *gsdf* uniquely in males. Shown are alignments of male- and female pool-seq data, nanopore reads and supernova pseudo-haplotype assemblies from the single male used to create the IMR_Hiphip.v1 assembly. A heterozygote 1.2 kb insertion is evident in nanopore read alignments as well as in the linked-read haplotype alignments and chr13Y carries the insertion allele. The insertion is less evident in the male short-read data due to poor mappability in the in/del region. Male pool short reads carrying chr13Y haplotype variant tags have fever red colored mates supporting the deletion allele (short read alignments are sorted by the SNP allele in the center red/blue box, blue boxes = chrY-allele, red boxes = chrX-allele). *gsdf* gene model in blue shows the extent of *gsdf* expression detected in Atlantic halibut adult testis and adult ovary and testis in Pacific halibut. The two *gsdf* gene models in red indicate isoforms detected only in males from three separate developmental stages.
(PDF)

S13 Fig Schematic representation of *gsdf* and *brx* PCR results. The forward *gsdf* primer is 481 nt upstream of the 1.2 kb chrY Gypsy-LTR insertion. The reverse primer is 251 nt into the LTR. The *brx* primers are described in Methods. The resulting PCR products are shown in an agarose gel. The same 20 individuals are shown for both primer pairs. Individuals 1–10 are phenotypic males and 11–20 are phenotypic females. PCR results show that *brx* classification and presence of Gypsy-LTR insertion are in agreement and distinguish all 10 males from females.
(PDF)

S14 Fig Atlantic halibut chrY and chrX carry similar numbers of missense variants in relation to Pacific halibut. **A** The Chromium pseudo-haplotype assemblies of the reference genome male individual represents chrY (pseudo-haplotype 1) and chrX (pseudo-haplotype 2) for the interval chr13:0–9 Mb. The Chromium scaffold ID is number 45 for both PH1 and PH2. Our assembly of Atlantic halibut chr13 is inverted in comparison with the Chromium pseudo-haplotypes as well as the syntenic Pacific halibut chromosome (CM023513.1). Syntenic regions corresponding to the location of the Atlantic halibut chrY-core (chr13:0–9 Mb) are indicated in the Figure. **B** Counts of SNP alleles uniquely observed in each of the Atlantic halibut pseudo-haplotype assemblies in the core X/Y region and in the corresponding region of Pacific halibut chromosome CM023513.1. It is clear that the overall divergence, as well as non-synonymous and synonymous divergence is higher between species than between Atlantic halibut X and. Y. **C** The table shows counts of missense and synonymous variants observed in pseudo-haplotypes 1 and 2 (PH1 and PH2) in reation to Pacific halibut gene models. Counts per Mb are shown in Pacific halibut coordinates along the syntenic chromosome CM023513.1. Only variants differing between PH1 and PH2 were included.
(PDF)

S15 Fig Allelic imbalance on chr13. Among X/Y differentiating pool-seq SNPs where different alleles were observed also in male and female RNA-seq (S4A Fig), 130 SNPs were retained due to both the X and Y alleles being observed in at least one read in at least 6/7 male samples. Out of these 130 SNPs, six displayed allelic imbalance in males (defined as all seven male samples expressing one allele in higher levels). For three of these six (in the genes FLOWR, WDR5 and an unknown transcript) the imbalance is towards the ChrX-allele, while for three (CKMT2, *gsdf* and an unknown transcript) the imbalance is towards the ChrY-allele. Nucleotide positions of SNPs are shown to the right of the heat-map. Figure generated in https://software.broadinstitute.org/morpheus/.
(PDF)

S16 Fig Theoretical evolution of sex chromosomes in a heterochiasmy setting. This model assumes linkage between sexually antagonistic (SA) loci and the sex determining gene being important. On top, three fictive autosome pairs (A^1^-A^3^) are shown. One of these acquires genetic material governing development of carriers into phenotypic males (left arrow) or phenotypic females (right arrow), thereby creating a novel sex chromosome. Thick black lines in between chromatids indicate the chromosomal interval undergoing meiotic recombination in each sex. The new sex determining gene (*) must occur in a region incapable of meiotic recombination in the heterogametic sex, or else, it does not become isolated from recombining with its former autosomal pair during meiosis. Arrows within boxes next to the sex determining gene location indicate the interval where a new sex determining gene could occur in the two respective systems, (XY or ZW).
(PDF)

S17 Fig Reference gene for qPCR. To find a suitable (stable) internal reference gene for the normalization of the qPCR data, we selected a few candidates from the RNA-seq dataset with a stable number of reads throughout the developmental stages available. **A** Expression of the selected genes *ints9*, *JKAMP* and *gtf3c6* in Atlantic halibut embryos and larvae, shown as number of reads. Data are shown as mean with SEM. N = 4. **B** The selected genes *ints9*, *JKAMP* and *gtf3c6* were selected for testing with qPCR on the actual samples to be analyzed for *gsdf* expression. Expression of in Atlantic halibut embryos and larvae, shown as cycle threshold (CT)-values. Data are shown as mean with SEM. N = 4–9. *gtf3c6* had the most stable expression throughout the developmental stages, and was therefore chosen as the internal reference gene in this study.
(PDF)

S1 Table LepMap3 derived genetic maps from analysis of Palaikostas et. al. data aligned to IMR_hiphip.v1. (XLSX)

S2 Table Classification of the Atlantic halibut genome into Male-only meiotic Recombination (MRR) or Female-only meiotic Recombination (FRR) regions based on LepMap3 derived genetic maps from analysis of Palaikostas et. al. data aligned to IMR_hiphip.v1 NR = neither recombines BR = Both recombine. (XLSX)

S3 Table Predicted location of centromeres in IMR_Hiphip.v1 coordinates. Analysis of data from Reid et al, 2007.
(XLSX)

S4 Table Parsed RepeatMasker output for major repeat classes. Values in columns E and up indicate the proportion of bases in each interval classified as that specific repeat superclass group.
(XLSX)

S5 Table Sniffles raw output from analysis of ONT reads vs. IMR_Hiphip.v1. (XLSX)

S6 Table Chromosome/LG naming across Atlantic halibut studies. (XLSX)

S1 File Integration site analysis of the 1.2 kb insertion upstream of *gsdf*, which controls male development, and related insertions in the Atlantic halibut genome. (PDF)

## References

[1] Kikuchi K, Hamaguchi S. Novel sex-determining genes in fish and sex chromosome evolution. Dev Dynam. 2013;242(4):339–53. doi: 10.1002/dvdy.23927 23335327

[2] Heule C, Salzburger W, Bohne A. Genetics of sexual development: an evolutionary playground for fish. Genetics. 2014;196(3):579–91. doi: 10.1534/genetics.114.161158 24653206 PMC3948791

[3] Todd EV, Liu H, Muncaster S, Gemmell NJ. Bending Genders: The Biology of Natural Sex Change in Fish. Sex Dev. 2016;10(5–6):223–41. doi: 10.1159/000449297 27820936

[4] Ross JA, Urton JR, Boland J, Shapiro MD, Peichel CL. Turnover of sex chromosomes in the stickleback fishes (gasterosteidae). PLoS genetics. 2009;5(2):e1000391. doi: 10.1371/journal.pgen.1000391 19229325 PMC2638011

[5] Cnaani A, Lee BY, Zilberman N, Ozouf-Costaz C, Hulata G, Ron M, et al. Genetics of sex determination in tilapiine species. Sex Dev. 2008;2(1):43–54. doi: 10.1159/000117718 18418034

[6] Franchini P, Jones JC, Xiong P, Kneitz S, Gompert Z, Warren WC, et al. Long-term experimental hybridisation results in the evolution of a new sex chromosome in swordtail fish. Nat Commun. 2018;9(1):5136. doi: 10.1038/s41467-018-07648-2 30510159 PMC6277394

[7] Kottler VA, Feron R, Nanda I, Klopp C, Du K, Kneitz S, et al. Independent Origin of XY and ZW Sex Determination Mechanisms in Mosquitofish Sister Species. Genetics. 2020;214(1):193–209. doi: 10.1534/genetics.119.302698 31704715 PMC6944411

[8] Herpin A, Schartl M. Plasticity of gene-regulatory networks controlling sex determination: of masters, slaves, usual suspects, newcomers, and usurpators. EMBO reports. 2015;16(10):1260–74. doi: 10.15252/embr.201540667 26358957 PMC4766460

[9] Yano A, Guyomard R, Nicol B, Jouanno E, Quillet E, Klopp C, et al. An immune-related gene evolved into the master sex-determining gene in rainbow trout, Oncorhynchus mykiss. Current biology: CB. 2012;22(15):1423–8. doi: 10.1016/j.cub.2012.05.045 22727696

[10] Bao L, Tian C, Liu S, Zhang Y, Elaswad A, Yuan Z, et al. The Y chromosome sequence of the channel catfish suggests novel sex determination mechanisms in teleost fish. BMC biology. 2019;17(1):6. doi: 10.1186/s12915-019-0627-7 30683095 PMC6346536

[11] Rafati N, Chen J, Herpin A, Pettersson ME, Han F, Feng C, et al. Reconstruction of the birth of a male sex chromosome present in Atlantic herring. Proc Natl Acad Sci U S A. 2020;117(39):24359–68. doi: 10.1073/pnas.2009925117 32938798 PMC7533707

[12] King AC, Gut M, Zenker AK. Shedding new light on early sex determination in zebrafish. Arch Toxicol. 2020;94(12):4143–58. doi: 10.1007/s00204-020-02915-y 32975586 PMC7655572

[13] Dufresnes C, Brelsford A, Baier F, Perrin N. When Sex Chromosomes Recombine Only in the Heterogametic Sex: Heterochiasmy and Heterogamety in Hyla Tree Frogs. Mol Biol Evol. 2021;38(1):192–200. doi: 10.1093/molbev/msaa201 32761205 PMC7782862

[14] Bergero R, Gardner J, Bader B, Yong L, Charlesworth D. Exaggerated heterochiasmy in a fish with sex-linked male coloration polymorphisms. Proc Natl Acad Sci U S A. 2019;116(14):6924–31. doi: 10.1073/pnas.1818486116 30894479 PMC6452659

[15] Jeffries DL, Lavanchy G, Sermier R, Sredl MJ, Miura I, Borzee A, et al. A rapid rate of sex-chromosome turnover and non-random transitions in true frogs. Nat Commun. 2018;9(1):4088. doi: 10.1038/s41467-018-06517-2 30291233 PMC6173717

[16] Sardell JM, Cheng CD, Dagilis AJ, Ishikawa A, Kitano J, Peichel CL, et al. Sex Differences in Recombination in Sticklebacks. G3-Genes Genom Genet. 2018;8(6):1971–83. doi: 10.1534/g3.118.200166 29632132 PMC5982825

[17] Luckenbach JA, Borski RJ, Daniels HV, Godwin J. Sex determination in flatfishes: Mechanisms and environmental influences. Semin Cell Dev Biol. 2009;20(3):256–63. doi: 10.1016/j.semcdb.2008.12.002 19121404

[18] Tvedt HB, Benfey TJ, Martin-Robichaud DJ, McGowan C, Reith M. Gynogenesis and sex determination in Atlantic halibut (Hippoglossus hippoglossus). Aquaculture. 2006;252(2–4):573–83.

[19] Palaiokostas C, Bekaert M, Davie A, Cowan ME, Oral M, Taggart JB, et al. Mapping the sex determination locus in the Atlantic halibut (Hippoglossus hippoglossus) using RAD sequencing. Bmc Genomics. 2013;14. doi: 10.1186/1471-2164-14-14 23957753 PMC3765698

[20] Reid DP, Smith CA, Rommens M, Blanchard B, Martin-Robichaud D, Reith M. A genetic linkage map of Atlantic halibut (Hippoglossus hippoglossus L.). Genetics. 2007;177(2):1193–205. doi: 10.1534/genetics.107.075374 17720928 PMC2034623

[21] Grant WS, Teel DJ, Kobayashi T, Schmitt C. Biochemical Population-Genetics of Pacific Halibut (Hippoglossus-Stenolepis) and Comparison with Atlantic Halibut (H-Hippoglossus). Can J Fish Aquat Sci. 1984;41(7):1083–8.

[22] Drinan DP, Loher T, Hauser L. Identification of Genomic Regions Associated With Sex in Pacific Halibut. J Hered. 2018;109(3):326–32. doi: 10.1093/jhered/esx102 29136178

[23] Brown NP, Bromage NR, Penman DJ, Shields RJ. The karyotype of the Atlantic halibut, Hippoglossus hippoglossus (Linnaeus). Aquac Res. 1997;28(7):489–91.

[24] Myosho T, Otake H, Masuyama H, Matsuda M, Kuroki Y, Fujiyama A, et al. Tracing the emergence of a novel sex-determining gene in medaka, Oryzias luzonensis. Genetics. 2012;191(1):163–70. doi: 10.1534/genetics.111.137497 22367037 PMC3338257

[25] Rondeau EB, Messmer AM, Sanderson DS, Jantzen SG, von Schalburg KR, Minkley DR, et al. Genomics of sablefish (Anoplopoma fimbria): expressed genes, mitochondrial phylogeny, linkage map and identification of a putative sex gene. Bmc Genomics. 2013;14:452. doi: 10.1186/1471-2164-14-452 23829495 PMC3708741

[26] Hendry CI, Martin-Robichaud DJ, Benfey TJ. Hormonal sex reversal of Atlantic halibut (Hippoglossus hippoglossus L.). Aquaculture. 2003;219(1–4):769–81.

[27] Babiak J, Babiak I, van Nes S, Harboe T, Haugen T, Norberg B. Induced sex reversal using an aromatase inhibitor, Fadrozole, in Atlantic halibut (Hippoglossus hippoglossus L.). Aquaculture. 2012;324:276–80.

[28] Vinnikov KA, Thomson RC, Munroe TA. Revised classification of the righteye flounders (Teleostei: Pleuronectidae) based on multilocus phylogeny with complete taxon sampling. Mol Phylogenet Evol. 2018;125:147–62. doi: 10.1016/j.ympev.2018.03.014 29535031

[29] Kumar S, Stecher G, Suleski M, Hedges SB. TimeTree: A Resource for Timelines, Timetrees, and Divergence Times. Molecular Biology and Evolution. 2017;34(7):1812–9. doi: 10.1093/molbev/msx116 28387841

[30] Tarailo-Graovac M, Chen N. Using RepeatMasker to identify repetitive elements in genomic sequences. Curr Protoc Bioinformatics. 2009;Chapter 4:Unit 4 10. doi: 10.1002/0471250953.bi0410s25 19274634

[31] Sedlazeck FJ, Rescheneder P, Smolka M, Fang H, Nattestad M, von Haeseler A, et al. Accurate detection of complex structural variations using single-molecule sequencing. Nat Methods. 2018;15(6):461–+. doi: 10.1038/s41592-018-0001-7 29713083 PMC5990442

[32] Hubley R, Finn RD, Clements J, Eddy SR, Jones TA, Bao WD, et al. The Dfam database of repetitive DNA families. Nucleic Acids Res. 2016;44(D1):D81–D9. doi: 10.1093/nar/gkv1272 26612867 PMC4702899

[33] Feng C, Pettersson M, Lamichhaney S, Rubin CJ, Rafati N, Casini M, et al. Moderate nucleotide diversity in the Atlantic herring is associated with a low mutation rate. Elife. 2017;6. doi: 10.7554/eLife.23907 28665273 PMC5524536

[34] Malinsky M, Svardal H, Tyers AM, Miska EA, Genner MJ, Turner GF, et al. Whole-genome sequences of Malawi cichlids reveal multiple radiations interconnected by gene flow. Nat Ecol Evol. 2018;2(12):1940–55. doi: 10.1038/s41559-018-0717-x 30455444 PMC6443041

[35] Einfeldt AL, Kess T, Messmer A, Duffy S, Wringe BF, Fisher J, et al. Chromosome level reference of Atlantic halibut Hippoglossus hippoglossus provides insight into the evolution of sexual determination systems. Mol Ecol Resour. 2021. doi: 10.1111/1755-0998.13369 33655659

[36] Bourque G, Burns KH, Gehring M, Gorbunova V, Seluanov A, Hammell M, et al. Ten things you should know about transposable elements. Genome Biol. 2018;19. doi: 10.1186/s13059-018-1398-0 30454069 PMC6240941

[37] Imai T, Saino K, Matsuda M. Mutation of Gonadal soma-derived factor induces medaka XY gonads to undergo ovarian development. Biochem Bioph Res Co. 2015;467(1):109–14. doi: 10.1016/j.bbrc.2015.09.112 26408909

[38] Sawatari E, Shikina S, Takeuchi T, Yoshizaki G. A novel transforming growth factor-beta superfamily member expressed in gonadal somatic cells enhances primordial germ cell and spermatogonial proliferation in rainbow trout (Oncorhynchus mykiss). Dev Biol. 2007;301(1):266–75. doi: 10.1016/j.ydbio.2006.10.001 17109839

[39] Dechaud C, Volff JN, Schartl M, Naville M. Sex and the TEs: transposable elements in sexual development and function in animals. Mob DNA. 2019;10:42. doi: 10.1186/s13100-019-0185-0 31700550 PMC6825717

[40] Faber-Hammond JJ, Phillips RB, Brown KH. Comparative Analysis of the Shared Sex-Determination Region (SDR) among Salmonid Fishes. Genome Biol Evol. 2015;7(7):1972–87. doi: 10.1093/gbe/evv123 26112966 PMC4524489

[41] Herpin A, Braasch I, Kraeussling M, Schmidt C, Thoma EC, Nakamura S, et al. Transcriptional rewiring of the sex determining dmrt1 gene duplicate by transposable elements. PLoS genetics. 2010;6(2):e1000844. doi: 10.1371/journal.pgen.1000844 20169179 PMC2820524

[42] Jangam D, Feschotte C, Betran E. Transposable Element Domestication As an Adaptation to Evolutionary Conflicts. Trends Genet. 2017;33(11):817–31. doi: 10.1016/j.tig.2017.07.011 28844698 PMC5659911

[43] Lenormand T. The evolution of sex dimorphism in recombination. Genetics. 2003;163(2):811–22. doi: 10.1093/genetics/163.2.811 12618416 PMC1462442

[44] Sutherland BJG, Rico C, Audet C, Bernatchez L. Sex Chromosome Evolution, Heterochiasmy, and Physiological QTL in the Salmonid Brook Charr Salvelinus fontinalis. G3 (Bethesda). 2017;7(8):2749–62. doi: 10.1534/g3.117.040915 28626004 PMC5555479

[45] Sardell JM, Kirkpatrick M. Sex Differences in the Recombination Landscape. Am Nat. 2020;195(2):361–79. doi: 10.1086/704943 32017625 PMC7537610

[46] Matsuda M, Sotoyama S, Hamaguchi S, Sakaizumi M. Male-specific restriction of recombination frequency in the sex chromosomes of the medaka, Oryzias latipes. Genet Res. 1999;73(3):225–31.

[47] Lemos B, Branco AT, Hartl DL. Epigenetic effects of polymorphic Y chromosomes modulate chromatin components, immune response, and sexual conflict. Proc Natl Acad Sci U S A. 2010;107(36):15826–31. doi: 10.1073/pnas.1010383107 20798037 PMC2936610

[48] Kent TV, Uzunovic J, Wright SI. Coevolution between transposable elements and recombination. Philos T R Soc B. 2017;372(1736). doi: 10.1098/rstb.2016.0458 29109221 PMC5698620

[49] McGaugh SE, Heil CSS, Manzano-Winkler B, Loewe L, Goldstein S, Himmel TL, et al. Recombination Modulates How Selection Affects Linked Sites in Drosophila. Plos Biol. 2012;10(11). doi: 10.1371/journal.pbio.1001422 23152720 PMC3496668

[50] Furman BLS, Metzger DCH, Darolti I, Wright AE, Sandkam BA, Almeida P, et al. Sex Chromosome Evolution: So Many Exceptions to the Rules. Genome Biol Evol. 2020;12(6):750–63. doi: 10.1093/gbe/evaa081 32315410 PMC7268786

[51] Kitano J, Ross JA, Mori S, Kume M, Jones FC, Chan YF, et al. A role for a neo-sex chromosome in stickleback speciation. Nature. 2009;461(7267):1079–83. doi: 10.1038/nature08441 19783981 PMC2776091

[52] Bachtrog D. Y-chromosome evolution: emerging insights into processes of Y-chromosome degeneration. Nat Rev Genet. 2013;14(2):113–24. doi: 10.1038/nrg3366 23329112 PMC4120474

[53] Chen SL, Zhang GJ, Shao CW, Huang QF, Liu G, Zhang P, et al. Whole-genome sequence of a flatfish provides insights into ZW sex chromosome evolution and adaptation to a benthic lifestyle. Nat Genet. 2014;46(3):253–+. doi: 10.1038/ng.2890 24487278

[54] Harboe T, Mangor-Jensen A, Naas KE, Naess T. A tank design for first feeding of Atlantic halibut, Hippoglossus hippoglossus L., larvae. Aquac Res. 1998;29(12):919–23.

[55] Harboe T, Tuene S, Mangorjensen A, Rabben H, Huse I. Design and Operation of an Incubator for Yolk-Sac Larvae of Atlantic Halibut. Prog Fish Cult. 1994;56(3):188–93.

[56] Norberg B, Valkner V, Huse J, Karlsen I, Grung GL. Ovulatory Rhythms and Egg Viability in the Atlantic Halibut (Hippoglossus-Hippoglossus). Aquaculture. 1991;97(4):365–71.

[57] Schneider CA, Rasband WS, Eliceiri KW. NIH Image to ImageJ: 25 years of image analysis. Nat Methods. 2012;9(7):671–5. doi: 10.1038/nmeth.2089 22930834 PMC5554542

[58] Minimap Li H. and miniasm: fast mapping and de novo assembly for noisy long sequences. Bioinformatics. 2016;32(14):2103–10. doi: 10.1093/bioinformatics/btw152 27153593 PMC4937194

[59] Vaser R, Sovic I, Nagarajan N, Sikic M. Fast and accurate de novo genome assembly from long uncorrected reads. Genome research. 2017;27(5):737–46. doi: 10.1101/gr.214270.116 28100585 PMC5411768

[60] Walker BJ, Abeel T, Shea T, Priest M, Abouelliel A, Sakthikumar S, et al. Pilon: An Integrated Tool for Comprehensive Microbial Variant Detection and Genome Assembly Improvement. Plos One. 2014;9(11). doi: 10.1371/journal.pone.0112963 25409509 PMC4237348

[61] Simao FA, Waterhouse RM, Ioannidis P, Kriventseva EV, Zdobnov EM. BUSCO: assessing genome assembly and annotation completeness with single-copy orthologs. Bioinformatics. 2015;31(19):3210–2. doi: 10.1093/bioinformatics/btv351 26059717

[62] Weisenfeld NI, Kumar V, Shah P, Church DM, Jaffe DB. Direct determination of diploid genome sequences. Genome research. 2017;27(5):757–67. doi: 10.1101/gr.214874.116 28381613 PMC5411770

[63] Dunn CW, Howison M, Zapata F. Agalma: an automated phylogenomics workflow. BMC bioinformatics. 2013;14:330. doi: 10.1186/1471-2105-14-330 24252138 PMC3840672

[64] Bolger AM, Lohse M, Usadel B. Trimmomatic: a flexible trimmer for Illumina sequence data. Bioinformatics. 2014;30(15):2114–20. doi: 10.1093/bioinformatics/btu170 24695404 PMC4103590

[65] Li H. Aligning sequence reads, clone sequences and assembly contigs with BWA-MEM. arXiv. 2013;1303.3997

[66] Van der Auwera GA, Carneiro MO, Hartl C, Poplin R, Del Angel G, Levy-Moonshine A, et al. From FastQ data to high confidence variant calls: the Genome Analysis Toolkit best practices pipeline. Curr Protoc Bioinformatics. 2013;43:11 0 1–0 33. doi: 10.1002/0471250953.bi1110s43 25431634 PMC4243306

[67] Langmead B, Salzberg SL. Fast gapped-read alignment with Bowtie 2. Nat Methods. 2012;9(4):357–9. doi: 10.1038/nmeth.1923 22388286 PMC3322381

[68] Kent WJ. BLAT—The BLAST-like alignment tool. Genome research. 2002;12(4):656–64. doi: 10.1101/gr.229202 11932250 PMC187518

[69] Edgar RC. MUSCLE: multiple sequence alignment with high accuracy and high throughput. Nucleic Acids Res. 2004;32(5):1792–7. doi: 10.1093/nar/gkh340 15034147 PMC390337

[70] Larsson A. AliView: a fast and lightweight alignment viewer and editor for large datasets. Bioinformatics. 2014;30(22):3276–8. doi: 10.1093/bioinformatics/btu531 25095880 PMC4221126

[71] Simpson JT, Workman RE, Zuzarte PC, David M, Dursi LJ, Timp W. Detecting DNA cytosine methylation using nanopore sequencing. Nat Methods. 2017;14(4):407–+. doi: 10.1038/nmeth.4184 28218898

[72] Li XY, Chen F, Chen YP. Gcluster: a simple-to-use tool for visualizing and comparing genome contexts for numerous genomes. Bioinformatics. 2020;36(12):3871–3. doi: 10.1093/bioinformatics/btaa212 32221617

[73] Dobin A, Davis CA, Schlesinger F, Drenkow J, Zaleski C, Jha S, et al. STAR: ultrafast universal RNA-seq aligner. Bioinformatics. 2013;29(1):15–21. doi: 10.1093/bioinformatics/bts635 23104886 PMC3530905

[74] Carrier E, Ferchaud AL, Normandeau E, Sirois P, Bernatchez L. Estimating the contribution of Greenland Halibut (Reinhardtius hippoglossoides) stocks to nurseries by means of genotyping-by-sequencing: Sex and time matter. Evol Appl. 2020;13(9):2155–67. doi: 10.1111/eva.12979 33005216 PMC7513701

[75] Grabherr MG, Russell P, Meyer M, Mauceli E, Alfoldi J, Di Palma F, et al. Genome-wide synteny through highly sensitive sequence alignment: Satsuma. Bioinformatics. 2010;26(9):1145–51. doi: 10.1093/bioinformatics/btq102 20208069 PMC2859124

[76] Quinlan AR, Hall IM. BEDTools: a flexible suite of utilities for comparing genomic features. Bioinformatics. 2010;26(6):841–2. doi: 10.1093/bioinformatics/btq033 20110278 PMC2832824

[77] Rastas P. Lep-MAP3: robust linkage mapping even for low-coverage whole genome sequencing data. Bioinformatics. 2017;33(23):3726–32. doi: 10.1093/bioinformatics/btx494 29036272

