# Supplementary material for: Heterochiasmy and the establishment of gsdf as a novel sex determining gene in Atlantic halibut
Source: PLoS Genet. 2022 Feb 8;18(2):e1010011. doi: 10.1371/journal.pgen.1010011 (PMC8824383; doi:10.1371/journal.pgen.1010011)
Supplement: S1 Fig — A Read length distribution of nanopore reads used to generate the IMR_Hiphip.v1 assembly. B Assembly continuities for the initial ONT contig assembly and the scaffolded IMR_Hiphip.v1 assembly. C Dot plot of genome to genome alignment between the IMR_hiphip.v1 assembly and the Pacific halibut reference genome assembly (IPHC_HiSten_1.0). Five chromosomes show larger inversions which are visible as green alignments perpendicular to the linear alignments (blue line) in the plot. A high degree of synteny was observed. (PDF) [file pgen.1010011.s001.pdf]

a

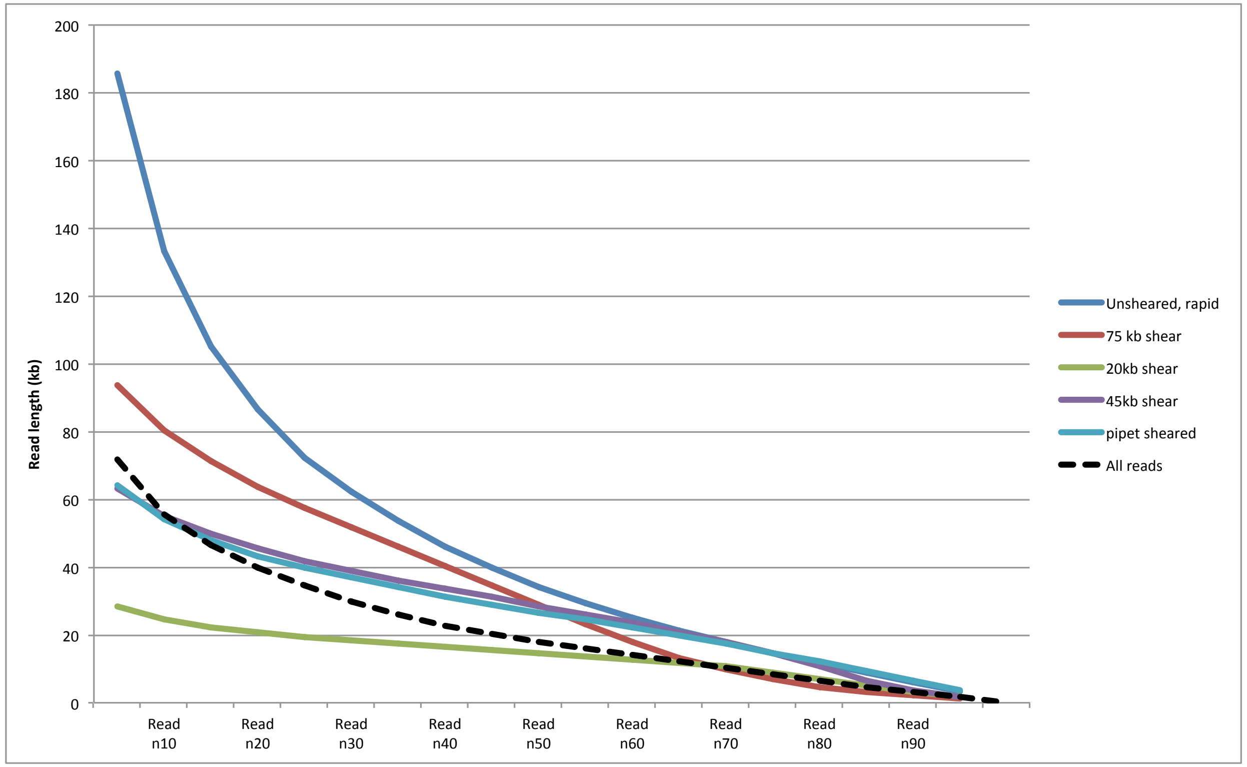

b

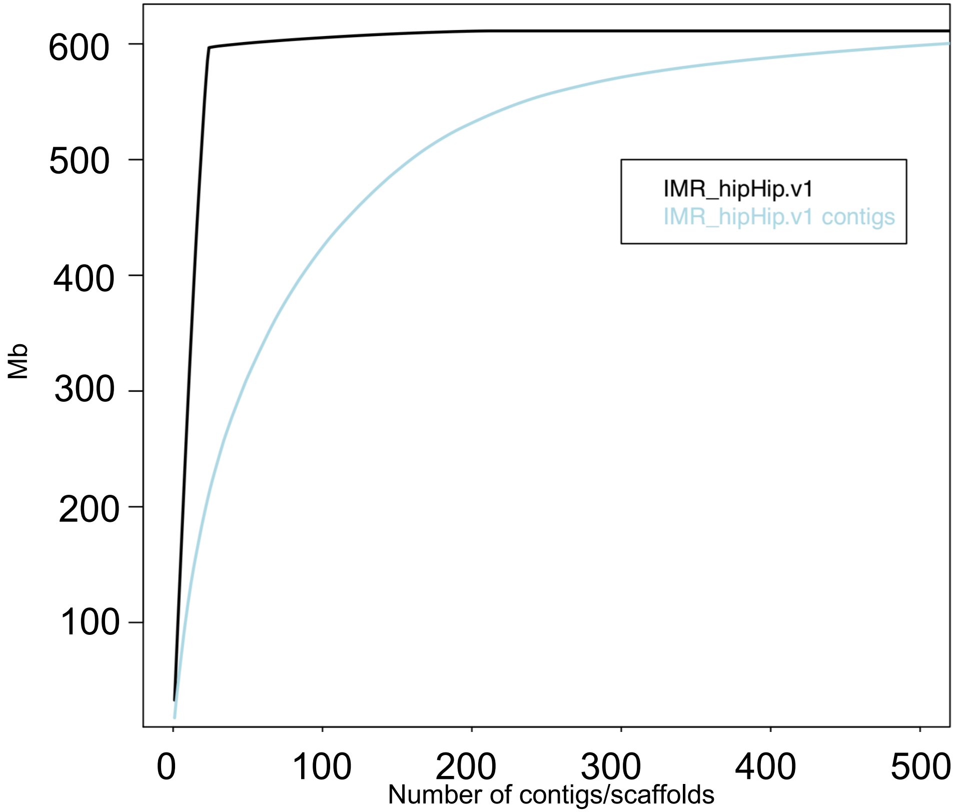

c

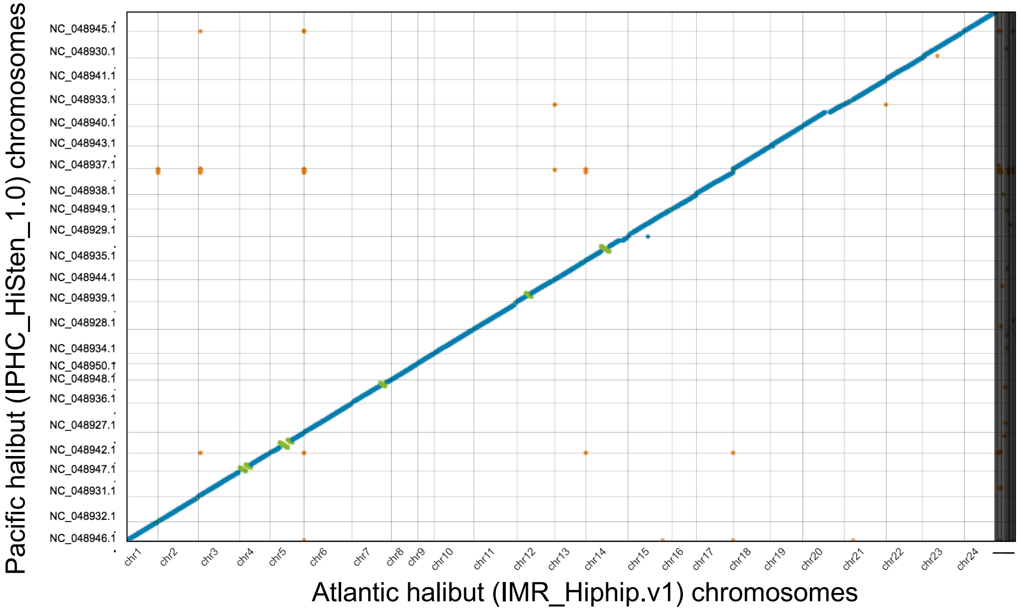

Supplementary Fig. 1: a Read length distribution of nanopore reads used to generate the IMR_Hiphip.v1 assembly. **b** Assembly continuities for the initial ONT contig assembly and the scaffolded IMR_Hiphip.v1 assembly. **c** Dot plot of genome to genome alignment between the IMR_hiphip.v1 assembly and the Pacific halibut reference genome assembly (IPHC_HiSten_1.0). Five chromosomes show larger inversions which are visible as green alignments perpendicular to the linear alignments (blue line) in the plot. A high degree of synteny was observed.
