# Supplementary material for: Heterochiasmy and the establishment of gsdf as a novel sex determining gene in Atlantic halibut
Source: PLoS Genet. 2022 Feb 8;18(2):e1010011. doi: 10.1371/journal.pgen.1010011 (PMC8824383; doi:10.1371/journal.pgen.1010011)
Supplement: S2 Fig — Scaffolding resulted in 24 major scaffolds, in agreement with the karyotype of Atlantic halibut. Chromosomes are sorted numerically 1–24 and are separated by white lines. (PDF) [file pgen.1010011.s002.pdf]

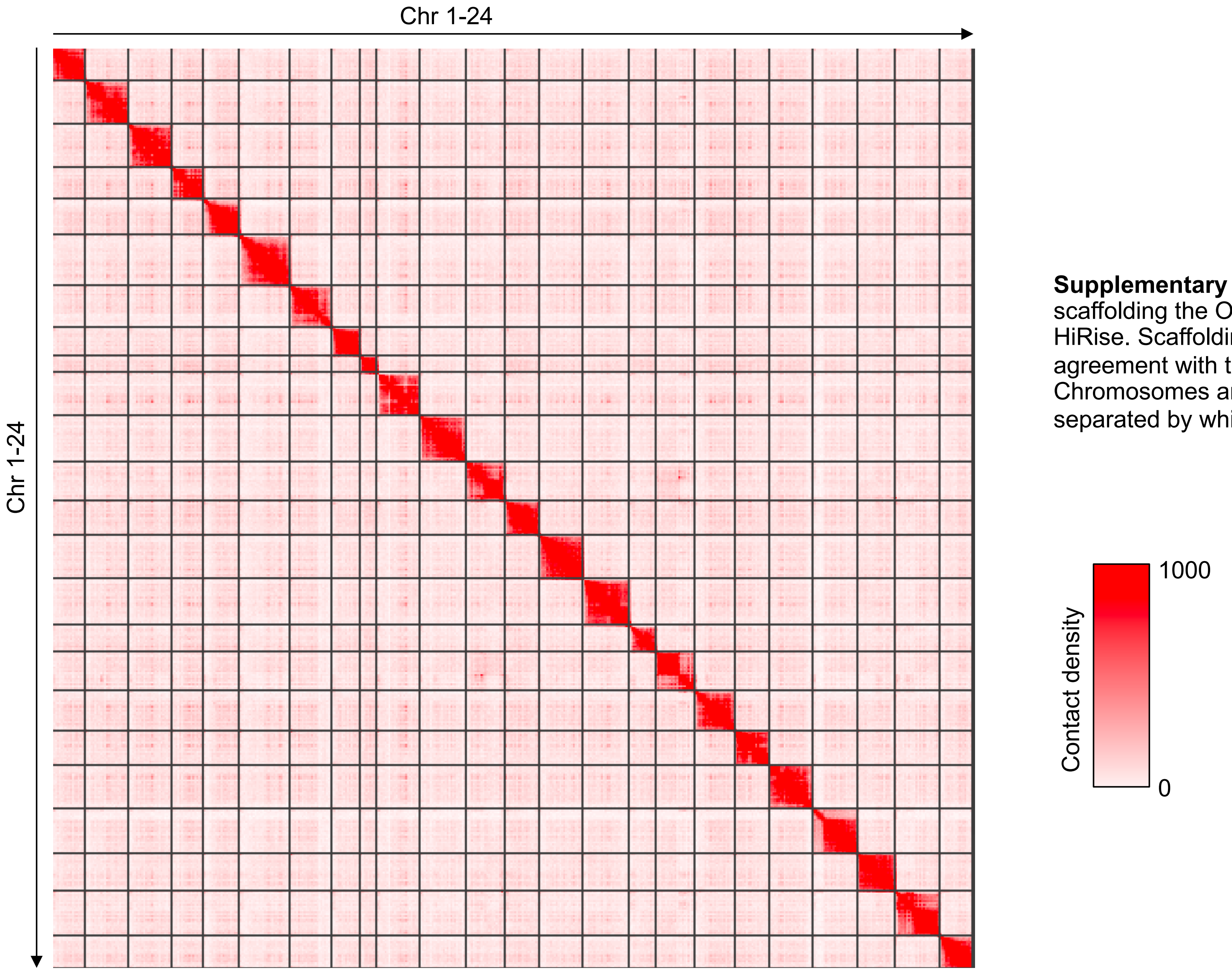

Supplementary Fig. 2: HiC contact matrix after scaffolding the Oxford Nanopore contig assembly using HiRise. Scaffolding resulted in 24 major scaffolds, in agreement with the karyotype of Atlantic halibut. Chromosomes are sorted numerically 1-24 and are separated by white lines.
