# Supplementary material for: Heterochiasmy and the establishment of gsdf as a novel sex determining gene in Atlantic halibut
Source: PLoS Genet. 2022 Feb 8;18(2):e1010011. doi: 10.1371/journal.pgen.1010011 (PMC8824383; doi:10.1371/journal.pgen.1010011)

Supplementary Fig. 3:
Principal component analysis of the RNA-seq data reveals clustering by maturation stage rather than genetic sex. Dots indicate coordinates of individual RNAseq samples on PC1 and PC2. Sample sames indicate their ages in days post fertilization (dpf). Box colors indicate the genetic sex assignment of samples.

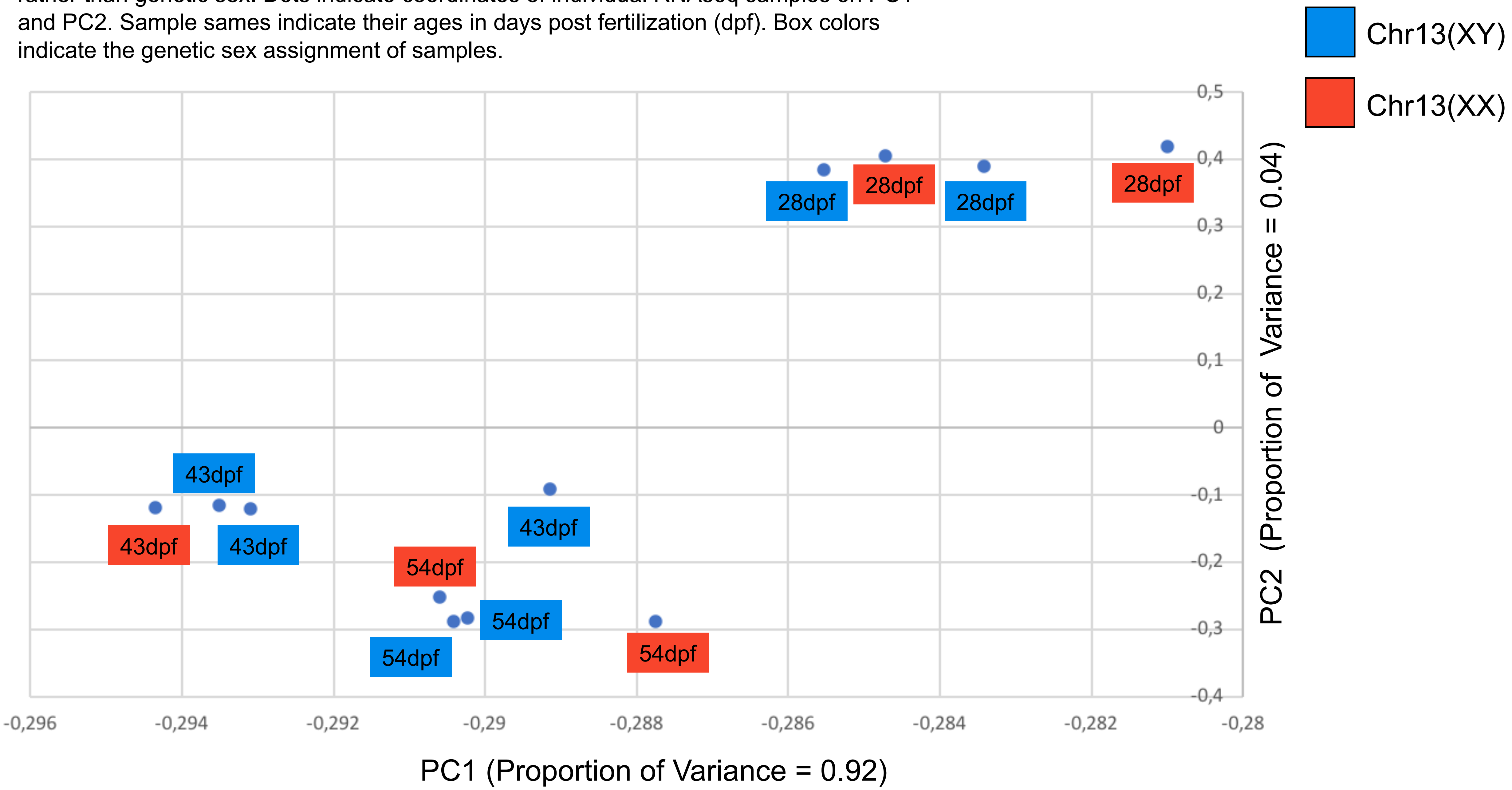

Supplement: S3 Fig — Dots indicate coordinates of individual RNAseq samples on PC1 and PC2. Sample sames indicate their ages in days post fertilization (dpf). Box colors indicate the genetic sex assignment of samples. (PDF) [file pgen.1010011.s003.pdf]
