# Supplementary material for: Heterochiasmy and the establishment of gsdf as a novel sex determining gene in Atlantic halibut
Source: PLoS Genet. 2022 Feb 8;18(2):e1010011. doi: 10.1371/journal.pgen.1010011 (PMC8824383; doi:10.1371/journal.pgen.1010011)

Supplementary Fig. 4:
a RNA-seq data samples clustered for 383 SNPs on chr13 previously found to be fixed in female DNA pool-seq while being variable in the male DNA pool-seq. We retained only positions were both alleles were observed in the RNA-seq data. Coordinates of genes along the Hiphip.v1 assembly are shown to the right. The samples without (NEG) *gsdf* expression in the RNA-seq and the samples with *gsdf* expression (POS) are in separate clusters. It was concluded that the individuals labeled XX and XY were female and male samples, respectively. **b** 65 SNPs fixed in the RNA-seq NEG samples but with both alleles observed in the RNA-seq POS samples along chr13. The nucleotide position (n) of the SNPs are shown to the right of the Figure. Made in https://software.broadinstitute.org/morpheus/

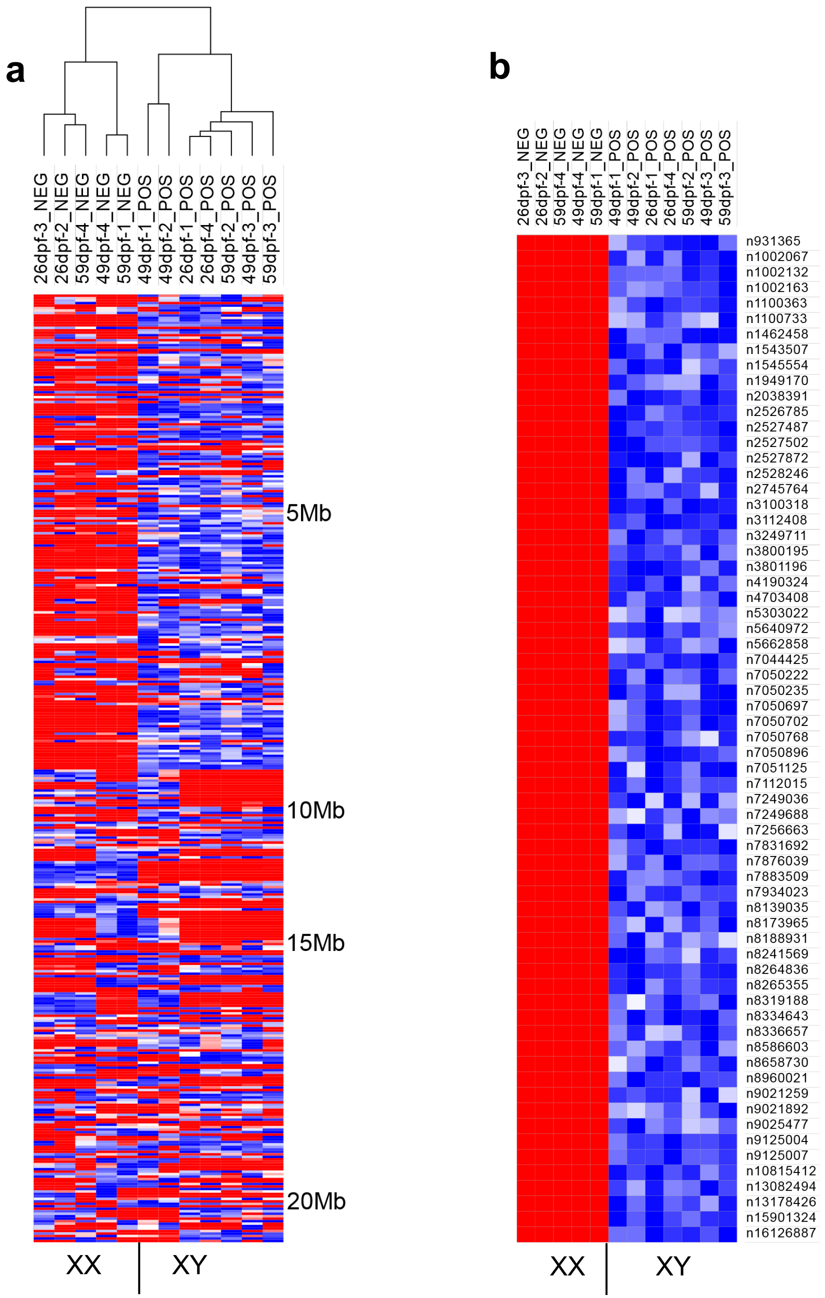

Supplement: S4 Fig — A RNA-seq data samples clustered for 383 SNPs on chr13 previously found to be fixed in female DNA pool-seq while being variable in the male DNA pool-seq. We retained only positions were both alleles were observed in the RNA-seq data. Coordinates of genes along the Hiphip.v1 assembly are shown to the right. The samples without (NEG) gsdf expression in the RNA-seq and the samples with gsdf expression (POS) are in separate clusters. It was concluded that the individuals labeled XX and XY were female and male samples, respectively. B 65 SNPs fixed in the RNA-seq NEG samples but with both alleles observed in the RNA-seq POS samples along chr13. The nucleotide positions (n) of the SNPs are shown to the right of the Figure. Made in https://software.broadinstitute.org/morpheus/. (PDF) [file pgen.1010011.s004.pdf]
