# Supplementary material for: Heterochiasmy and the establishment of gsdf as a novel sex determining gene in Atlantic halibut
Source: PLoS Genet. 2022 Feb 8;18(2):e1010011. doi: 10.1371/journal.pgen.1010011 (PMC8824383; doi:10.1371/journal.pgen.1010011)

Supplementary Fig.5: Genetic sex assignment by PCR. Sex differentiating PCR using sex specific primers (Table 1 in the manuscript) on cDNA from Atlantic halibut embryos at different developmental stages. Control samples were genomic DNA from individuals with known sex. Genetic sex of the samples was determined using a PCR based assay designed to differentiate genetic female and males. The forward primer was common for chr13X and chr13Y, while the reverse primers differed for two nucleotides in the 3'UTR of brx on chr13:9125004-9125007. For each individual sample (marked with a number) both female specific primers (brx_fwd + brx_femalerev; left well) and male specific primers (brx_fw + brx_malerev; right well) were run. In the case of a female sample, one band is detected in the left well while no band is detected in the right well. In the case of a male sample, two bands are detected. Genetic sex could not be determined for the 1, 8 and 24 hpf samples based on cDNA since this is prior to zygotic transcription of brx and the amount of gDNA extracted from these early stages were insufficient for reliable PCR.

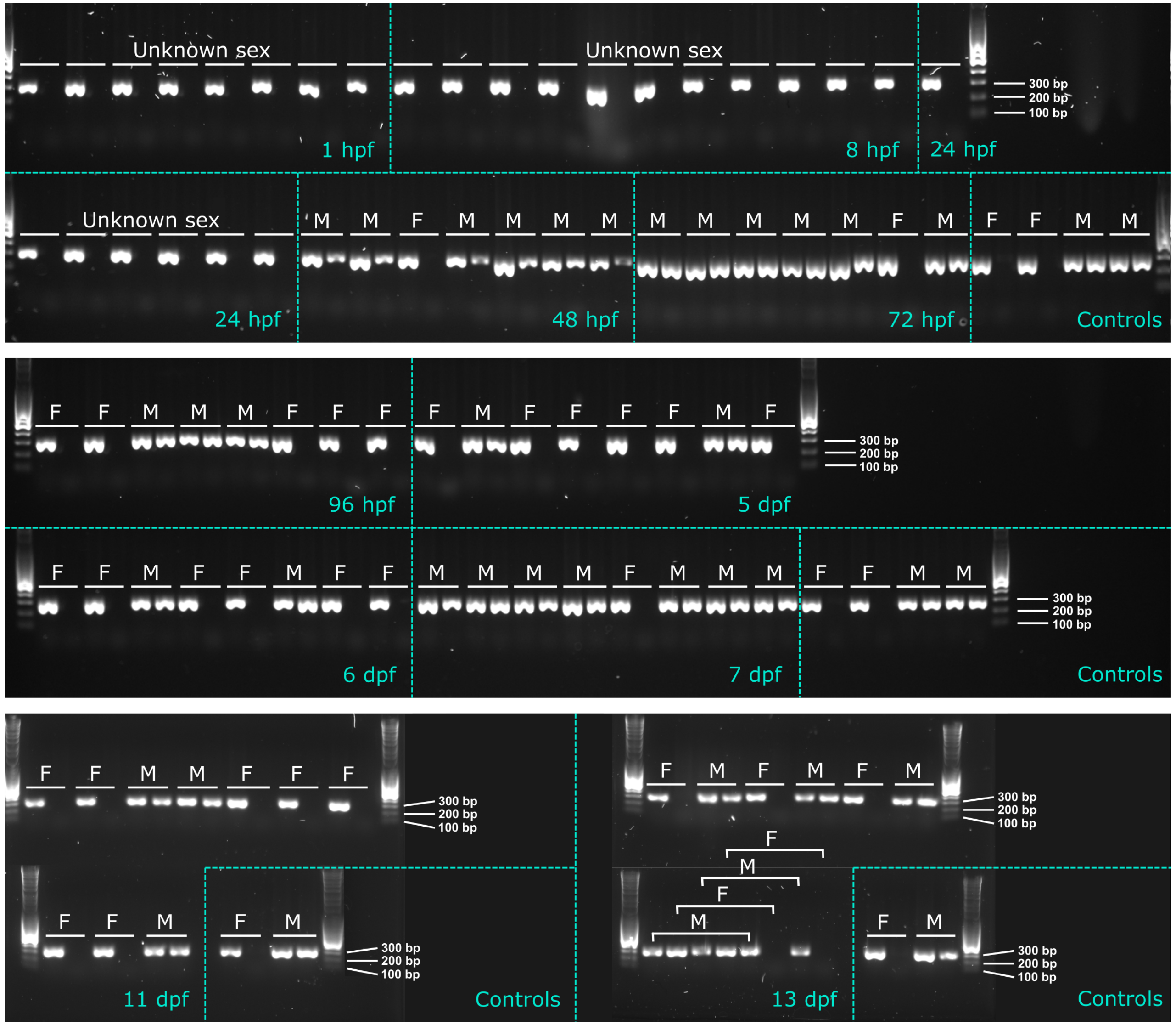

Supplement: S5 Fig — Sex differentiating PCR using sex specific primers (Table 1 in the manuscript) on cDNA from Atlantic halibut embryos at different developmental stages. Control samples were genomic DNA from individuals with known sex. Genetic sex of the samples was determined using a PCR based assay designed to differentiate genetic female and males. The forward primer was common for chr13X and chr13Y, while the reverse primers differed for two nucleotides in the 3’UTR of brx on chr13:9125004–9125007. For each individual sample (marked with a number) both female specific primers (brx_fwd + brx_femalerev; left well) and male specific primers (brx_fw + brx_malerev; right well) were run. In the case of a female sample, one band is detected in the left well while no band is detected in the right well. In the case of a male sample, two bands are detected. Genetic sex could not be determined for the 1, 8 and 24 hpf samples based on cDNA since this is prior to zygotic transcription of brx and the amount of gDNA extracted from these early stages were insufficient for reliable PCR. (PDF) [file pgen.1010011.s005.pdf]
