# Supplementary material for: Heterochiasmy and the establishment of gsdf as a novel sex determining gene in Atlantic halibut
Source: PLoS Genet. 2022 Feb 8;18(2):e1010011. doi: 10.1371/journal.pgen.1010011 (PMC8824383; doi:10.1371/journal.pgen.1010011)
Supplement: S6 Fig — The diagram is not to scale for the lengths of each gene nor the distance between genes. NCBI (http://www.ncbi.nlm.nih.gov) and Ensembl (http://www.ensembl.org) genome browsers were used to ascertain order, identity and orientation of genes immediately adjacent to gsdf. (PDF) [file pgen.1010011.s006.pdf]

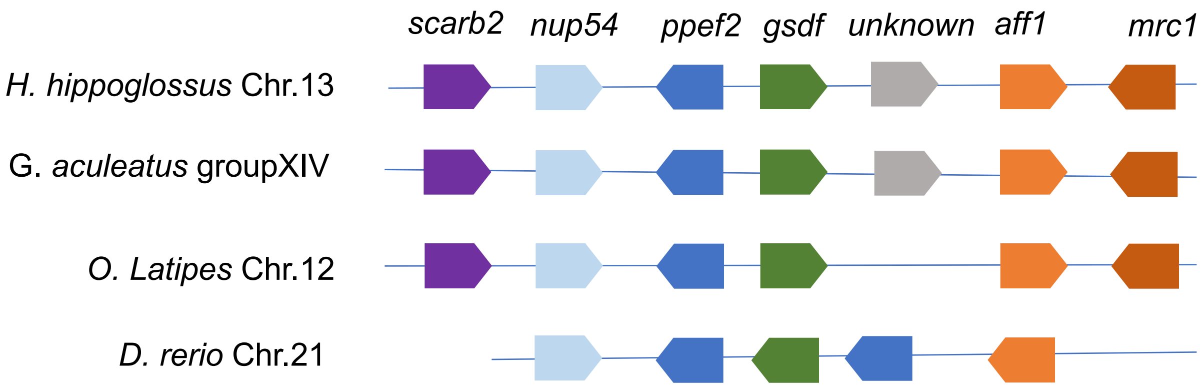

Supplementary Fig. 6: Synteny of chromosomal regions associated with *gsdf. G*ene lengths and intergenic lengths are not to scale. NCBI (http://www.ncbi.nlm.nih.gov) and Ensembl (http://www.ensembl.org) genome browsers were used to ascertain order, identity and orientation of genes immediately adjacent to *gsdf*.
