# Supplementary material for: Heterochiasmy and the establishment of gsdf as a novel sex determining gene in Atlantic halibut
Source: PLoS Genet. 2022 Feb 8;18(2):e1010011. doi: 10.1371/journal.pgen.1010011 (PMC8824383; doi:10.1371/journal.pgen.1010011)
Supplement: S7 Fig — A XY-scatter plot of the cumulative genetic distances of chromosomally anchored linkage map markers in males and females reveals a large extent of heterochiasmy. B MRRs are smaller but have higher recombination rate. Differences in effective recombination rate (cM/Mb) between the Male-only and Female-only meiotic Recombination regions (MRRs/FRRs) as a function of the fraction of chromosomal size annotated as MRR. Shown on the X-axis is MRR size divided by chromosome length. Shown on the Y-axis is the recombination rate (cM/Mb) for each MRR divided by the rate for the corresponding FRR. Circle sizes are proportional to chromosome lengths. The Atlantic halibut sex chromosome (chr13) is indicated by an arrow. Chr9, the smallest chromosome, is the only chromosome having a larger MRR than FRR as well as a higher female effective recombination rate. (PDF) [file pgen.1010011.s007.pdf]

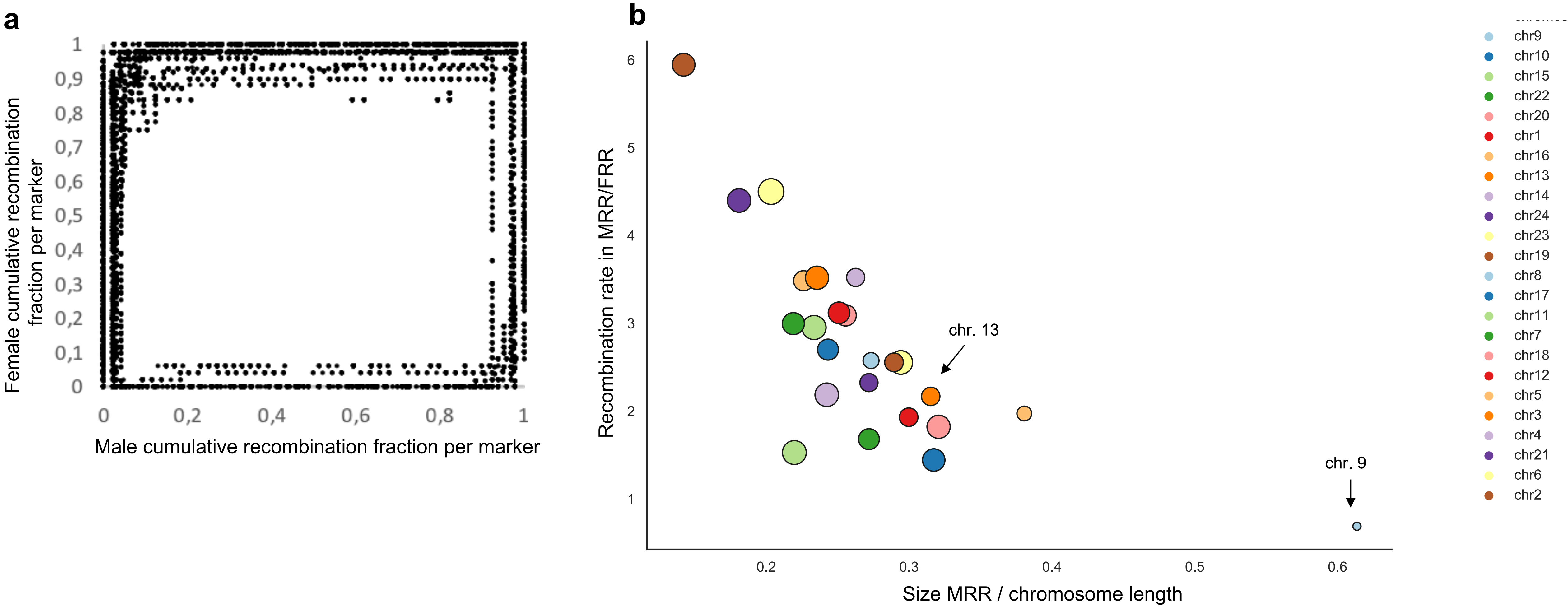

Supplementary Fig. 7: a XY-scatter plot of the cumulative genetic distances of chromosomally anchored linkage map markers in males and females reveals a large extent of heterochiasmy. **b** MRRs are smaller but have higher recombination rate. Differences in effective recombination rate (cM/Mb) between the Male-only and Female-only meiotic Recombination regions (MRRs/FRRs) as a function of the fraction of chromosomal size annotated as MRR. Shown on the X-axis is MRR size divided by chromosome length. Shown on the Y-axis is the recombination rate (cM/Mb) for each MRR divided by the rate for the corresponding FRR. Circle sizes are proportional to chromosome lengths. The Atlantic halibut sex chromosome (chr13) is indicated by an arrow. Chr9, the smallest chromosome, is the only chromosome having a larger MRR than FRR as well as a higher female effective recombination rate
