# Supplementary material for: Heterochiasmy and the establishment of gsdf as a novel sex determining gene in Atlantic halibut
Source: PLoS Genet. 2022 Feb 8;18(2):e1010011. doi: 10.1371/journal.pgen.1010011 (PMC8824383; doi:10.1371/journal.pgen.1010011)
Supplement: S8 Fig — On the x-axis: the log2 fold change (M-value) for recombination rate observed in MRRs and FRRs. On the y-axis: the log2 fold change (M-value) for nucleotide diversity in MRRs and FRRs. Circle colors indicate the chromosome and circle sizes are proportional to the relative size of the MRR on each chromosome (size MRR/ chr size). Chr13 (the sex chromosome) shows the largest difference of all chromosomes for MRR/FRR nucleotide diversity M-value. The FRR of chr13 is the X/Y chromosome. (PDF) [file pgen.1010011.s008.pdf]

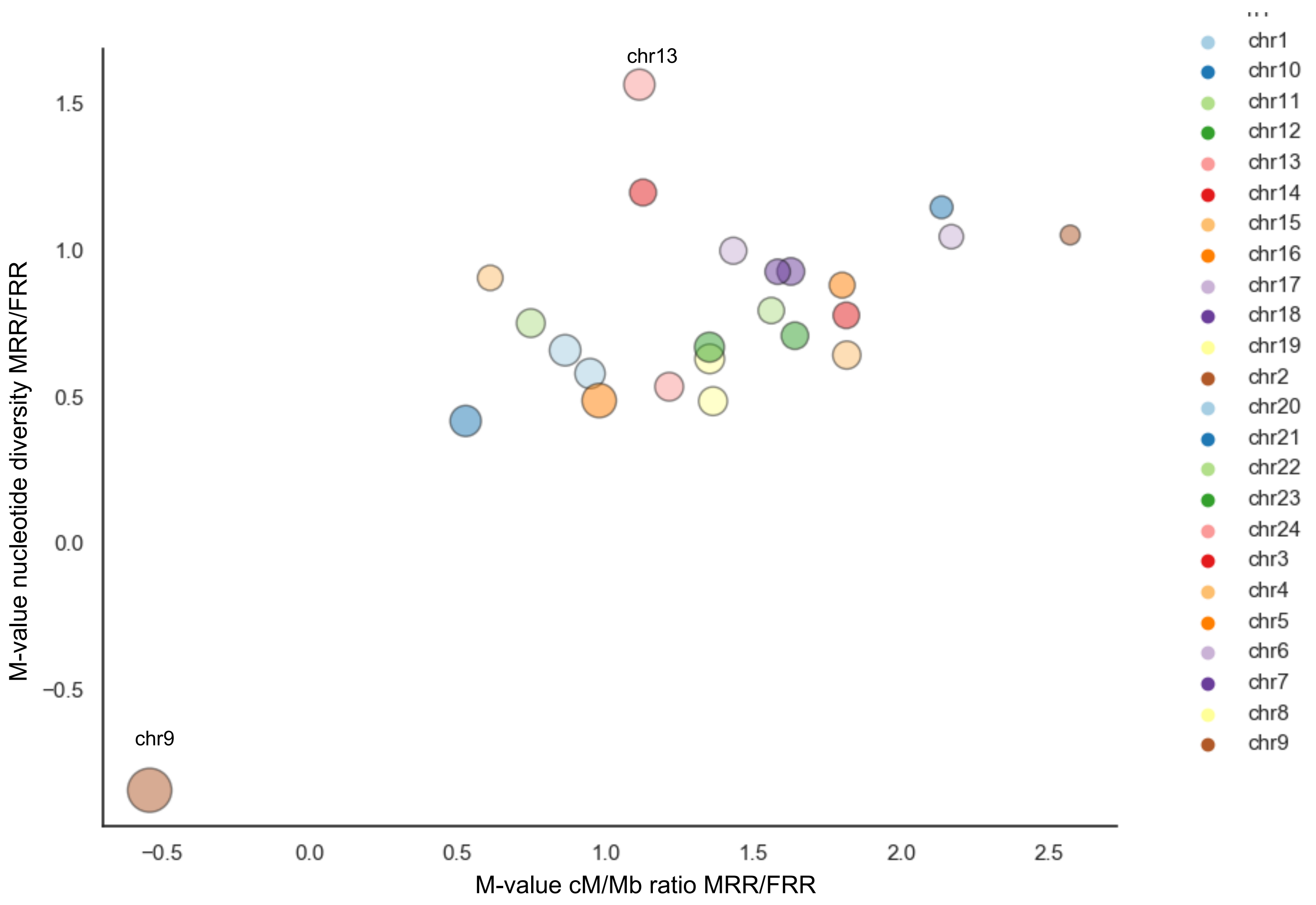

Supplementary Fig. 8:
The relationship between nucleotide diversity and recombination rate for MRRs and FRRs. On the x-axis: the log2 fold change (M-value) for recombination rate observed in MRRs and FRRs. On the y-axis: the log2 fold change (M-value) for nucleotide diversity in MRRs and FRRs. Circle colors indicate the chromosome and circle sizes are proportional to the relative size of the MRR on each chromosome (size MRR/ chr size). Chr13 (the sex chromosome) shows the largest difference of all chromosomes for MRR/FRR nucleotide diversity M-value. The FRR of chr13 is the X/Y chromosome.
