# Supplementary material for: Heterochiasmy and the establishment of gsdf as a novel sex determining gene in Atlantic halibut
Source: PLoS Genet. 2022 Feb 8;18(2):e1010011. doi: 10.1371/journal.pgen.1010011 (PMC8824383; doi:10.1371/journal.pgen.1010011)
Supplement: S9 Fig — The outmost track visualizes the individual chromosomes. Locations of MRRs and FRRs are shown as alternating black/grey rectangular tracks beneath each chromosome. Densities of repeat superclasses in 500 kb windows are indicated as dot plots in the five innermost tracks (LINE and SINE represent Long- and short interspersed nucleotide elements, respectively. LTR indicates Long Terminal Repeats. LCR and SR indicate low complexity- and simple repeats, respectively). (PDF) [file pgen.1010011.s009.pdf]

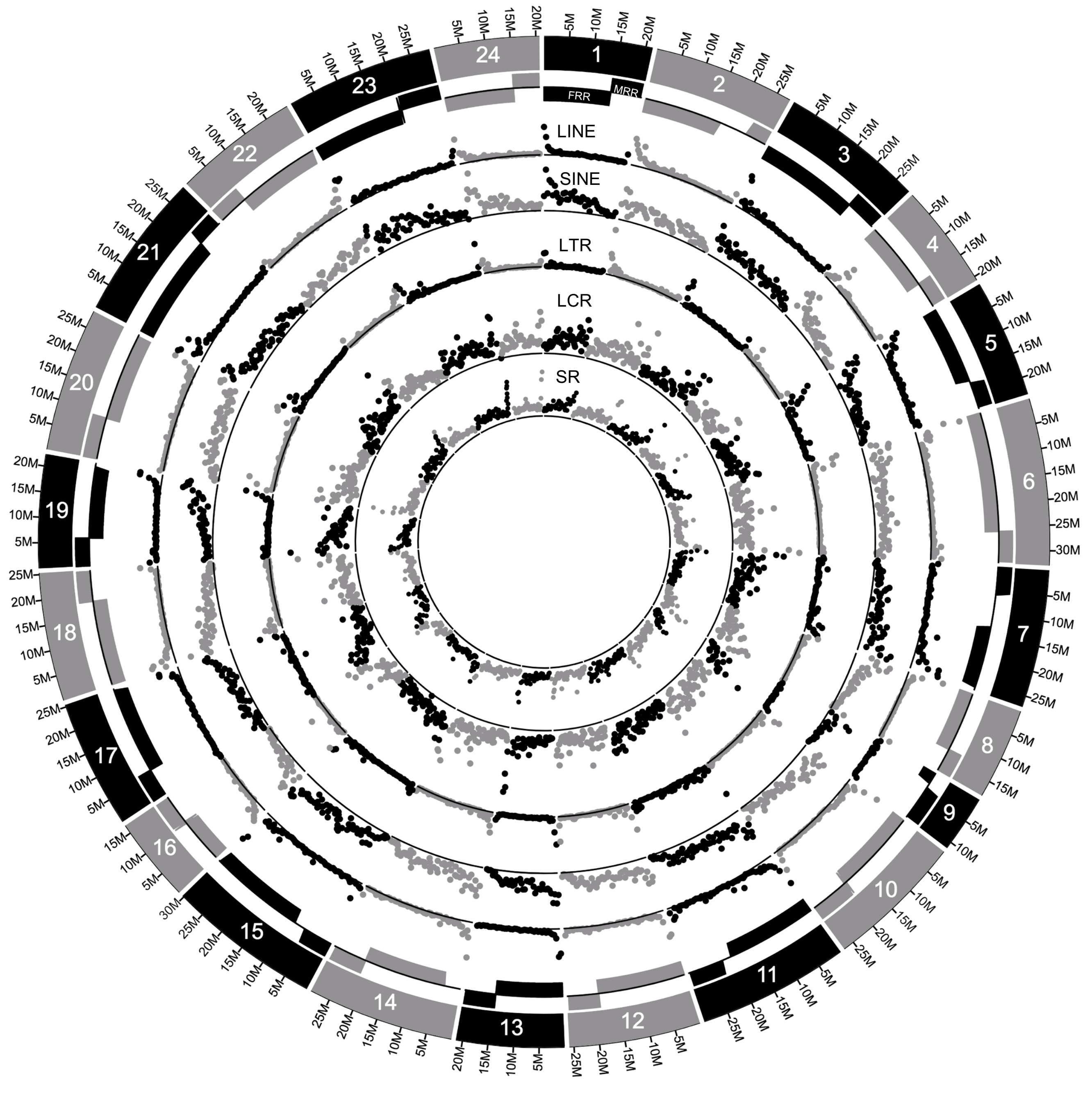

Supplementary Fig. 9:
Circular representation of repeat class content in the Atlantic halibut genome. The outmost track visualizes the individual chromosomes. Locations of MRRs and FRRs are shown as alternating black/grey rectangular tracks beneath each chromosome. Densities of repeat superclasses in 500 kb windows are indicated as dot plots in the five innermost tracks (LINE and SINE represent Long- and short interspersed nucleotide elements, respectively. LTR indicates Long Terminal Repeats. LCR and SR indicate low complexity- and simple repeats, respectively).
