# Supplementary figures and images for: Heterochiasmy and the establishment of gsdf as a novel sex determining gene in Atlantic halibut
Source: PLoS Genet. 2022 Feb 8;18(2):e1010011. doi: 10.1371/journal.pgen.1010011 (PMC8824383; doi:10.1371/journal.pgen.1010011)

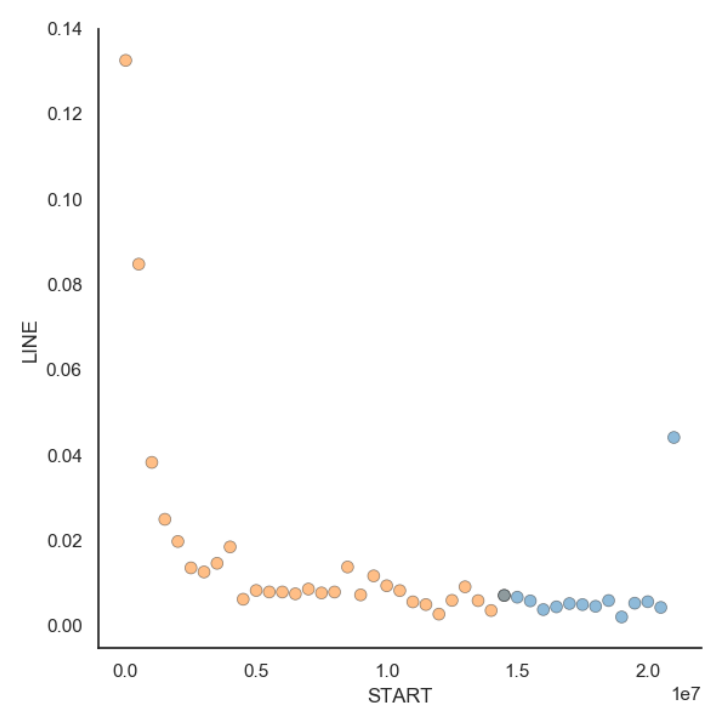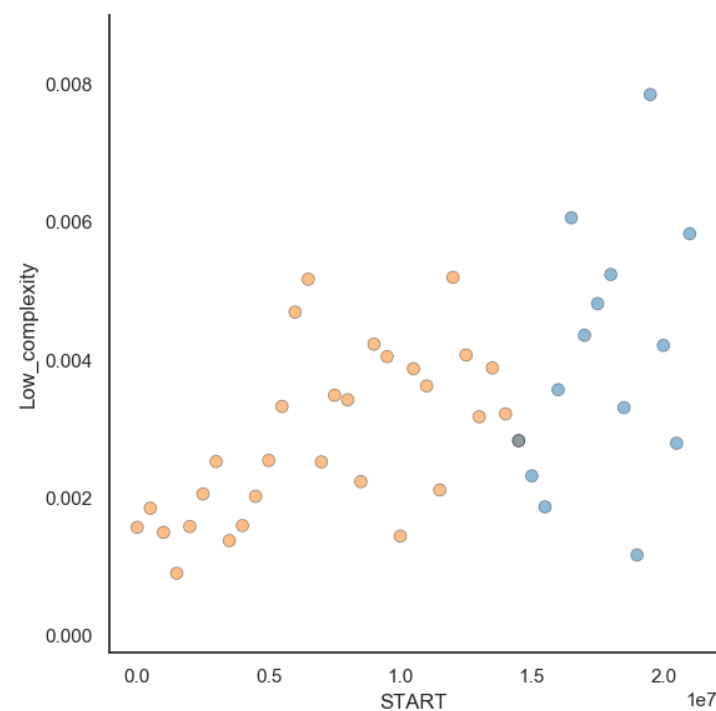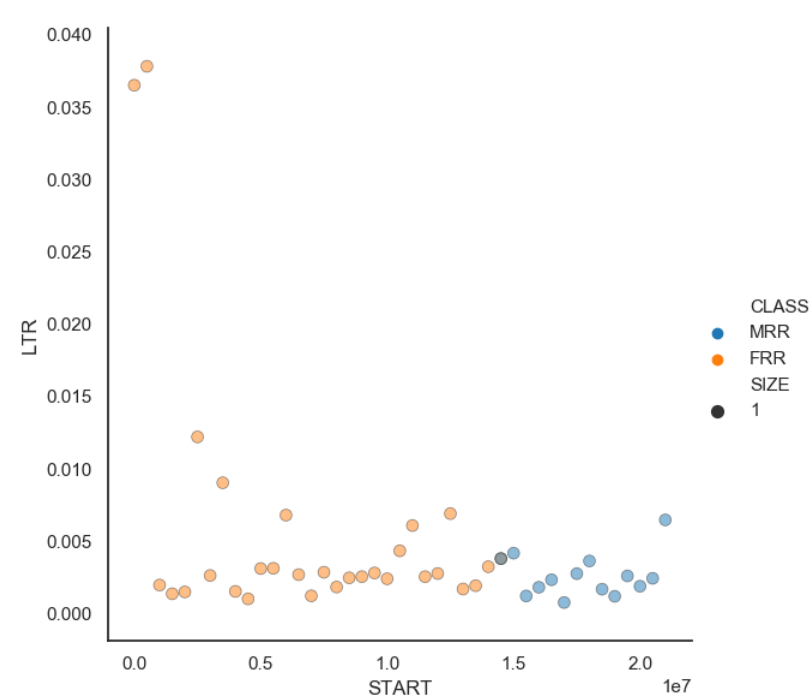

chr 1

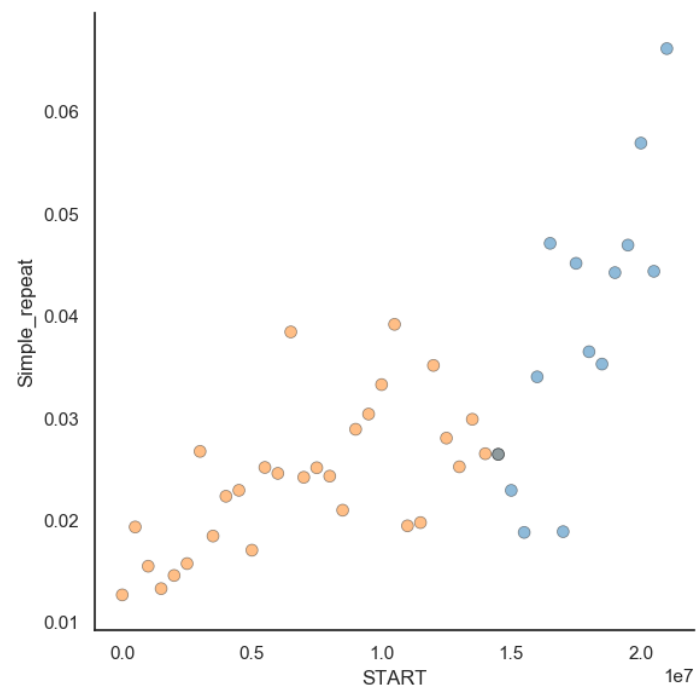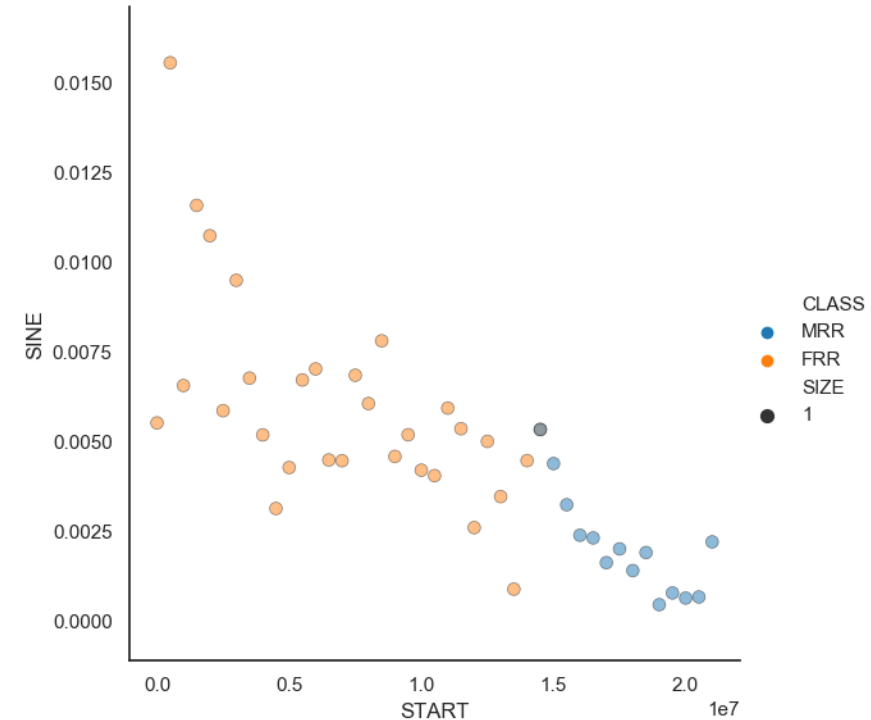

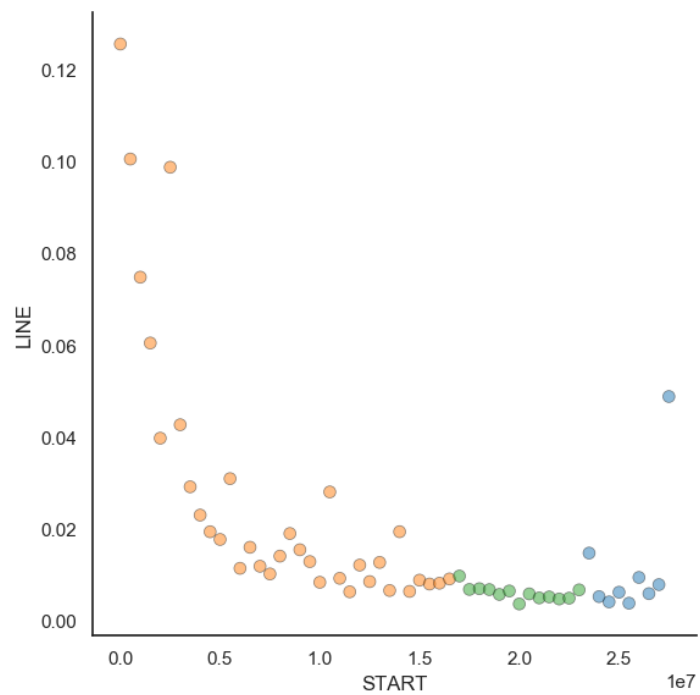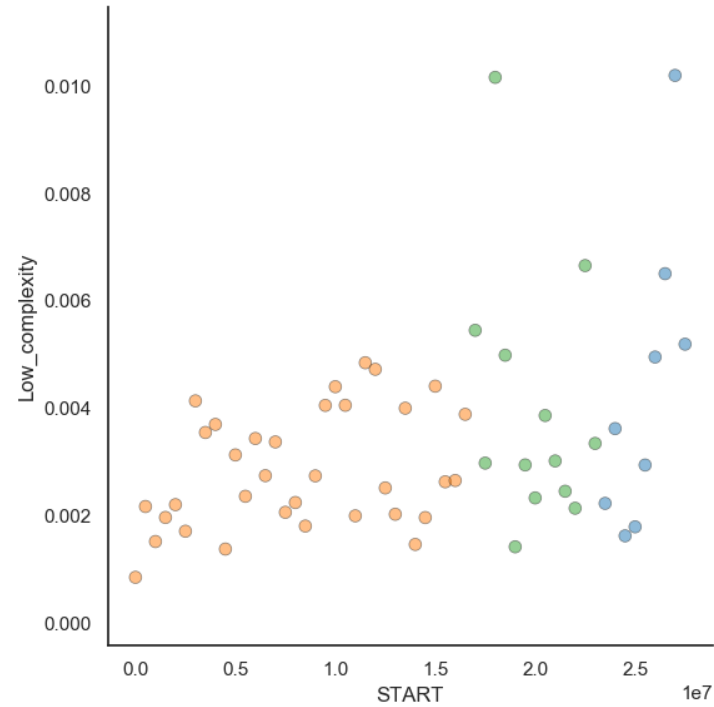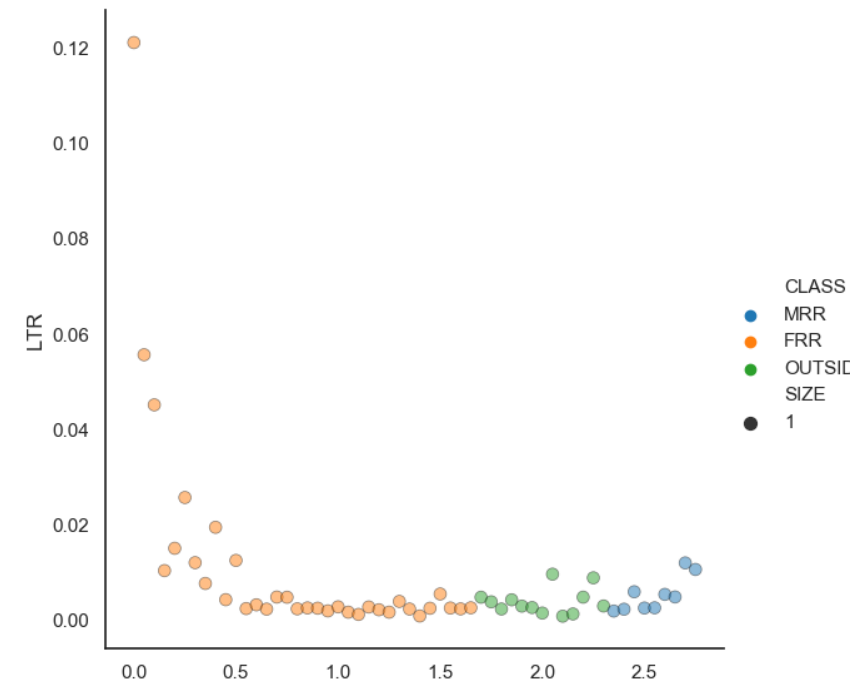

chr 2

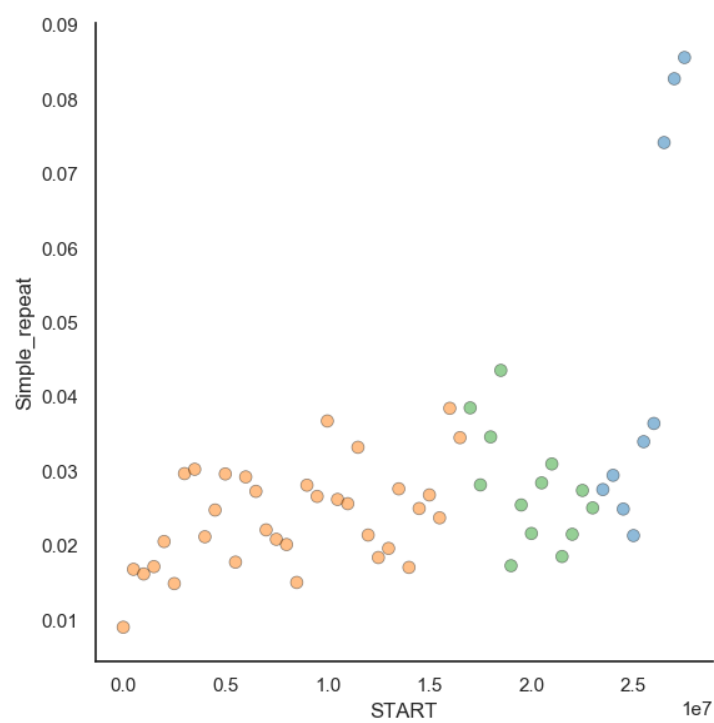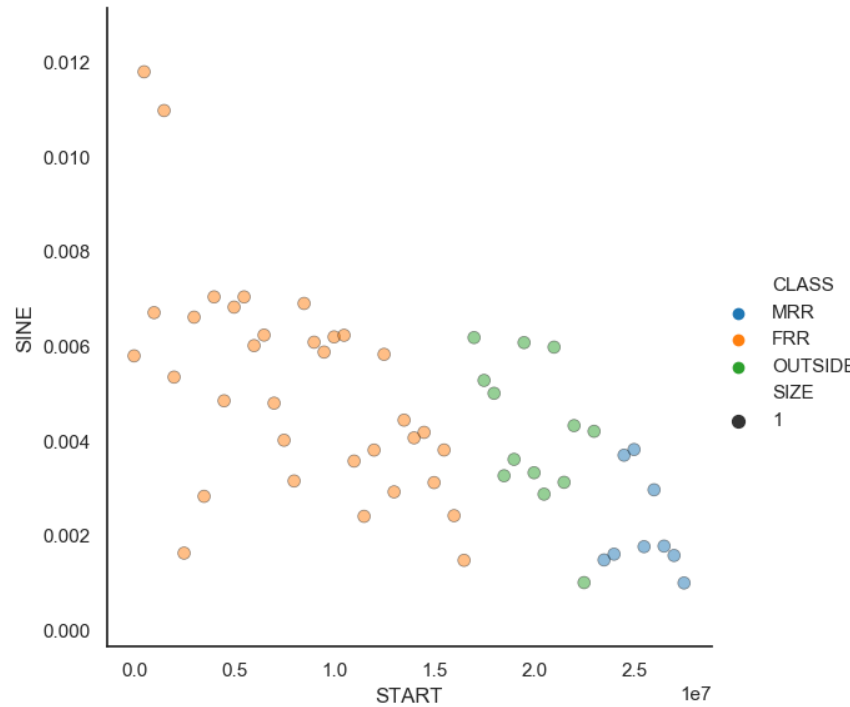

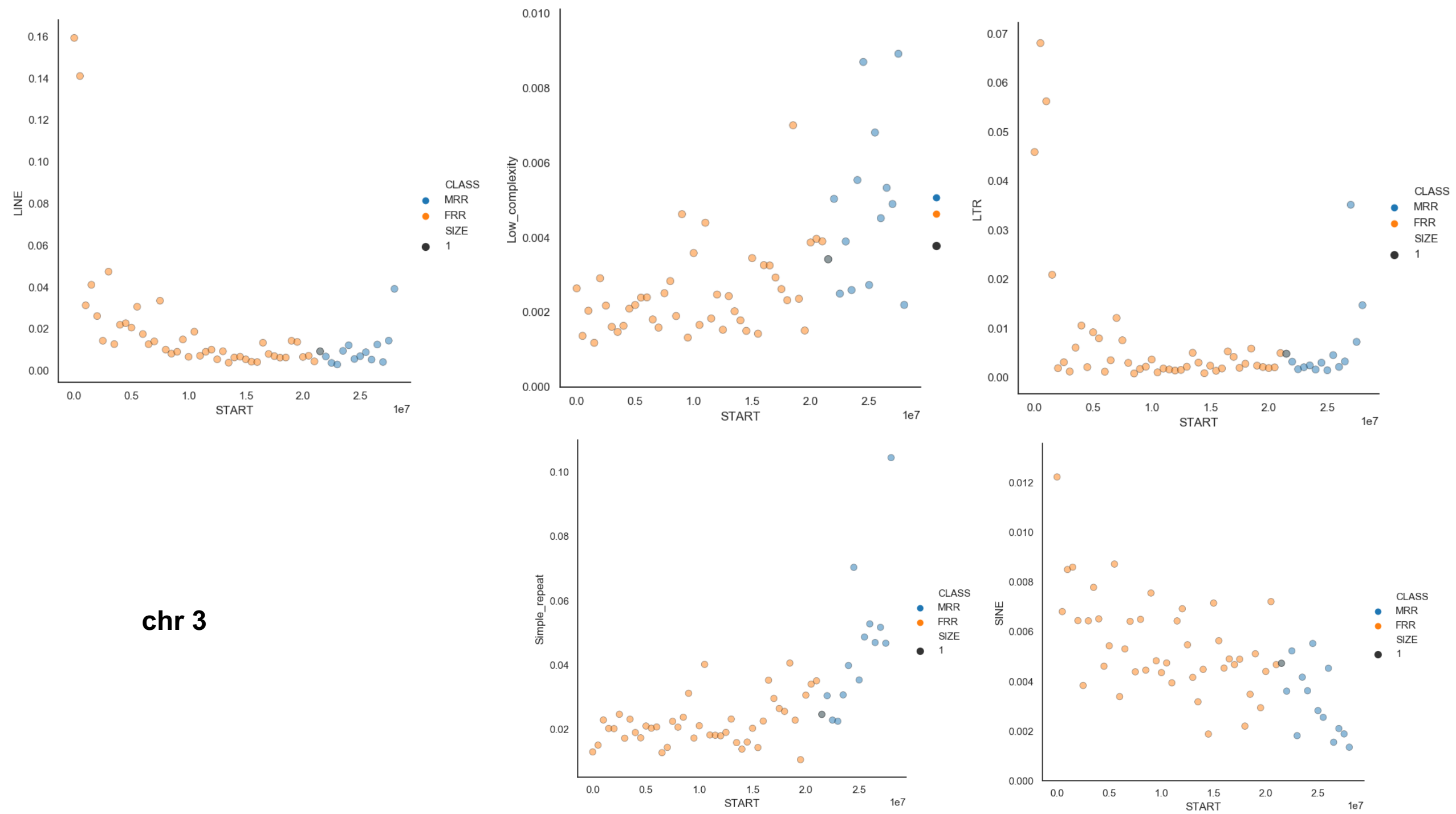

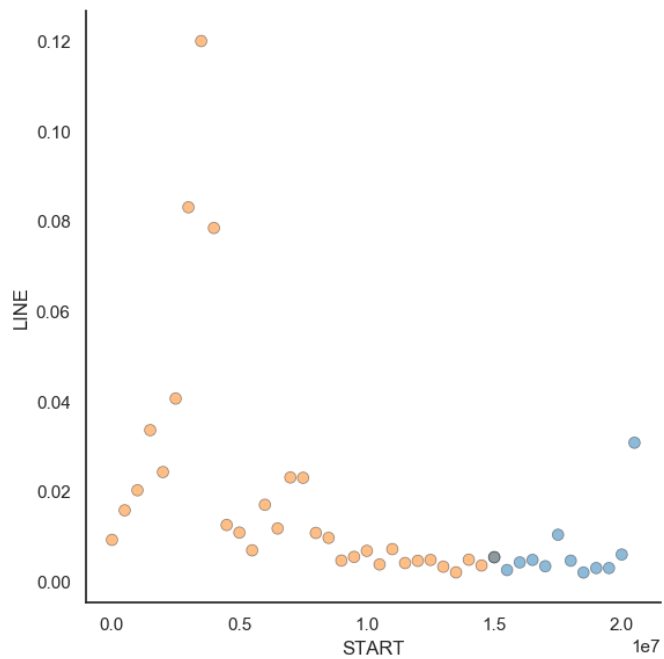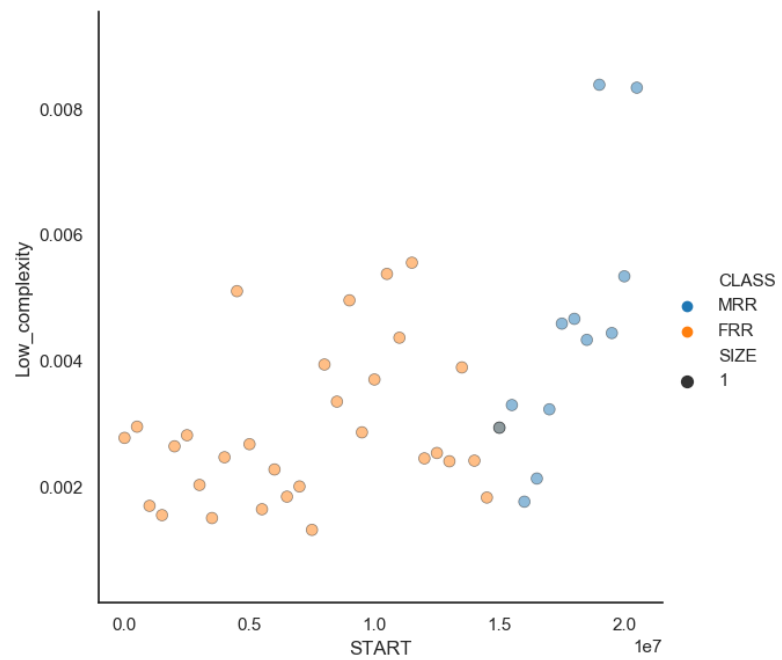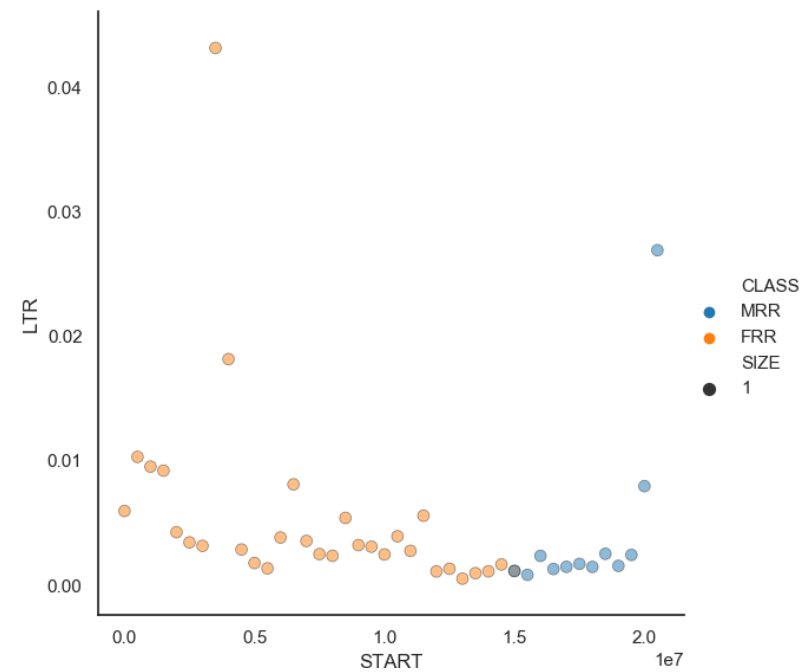

chr 4

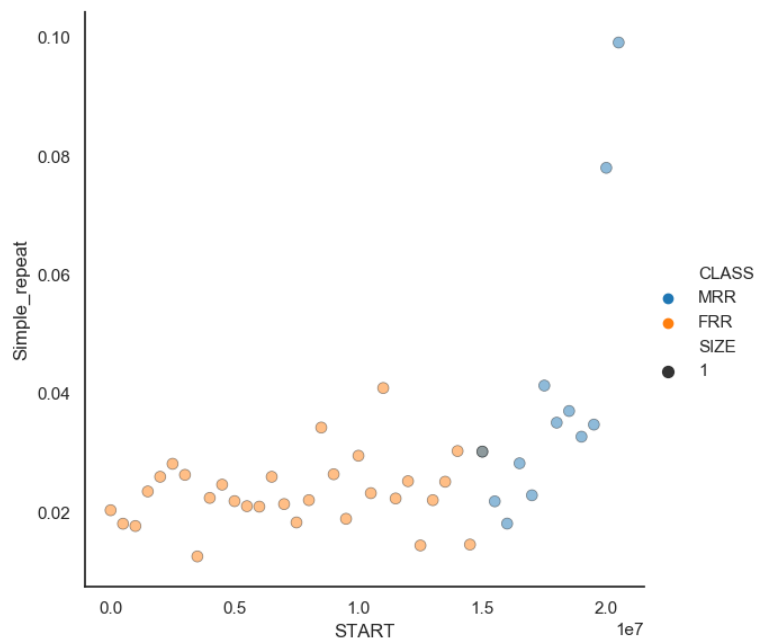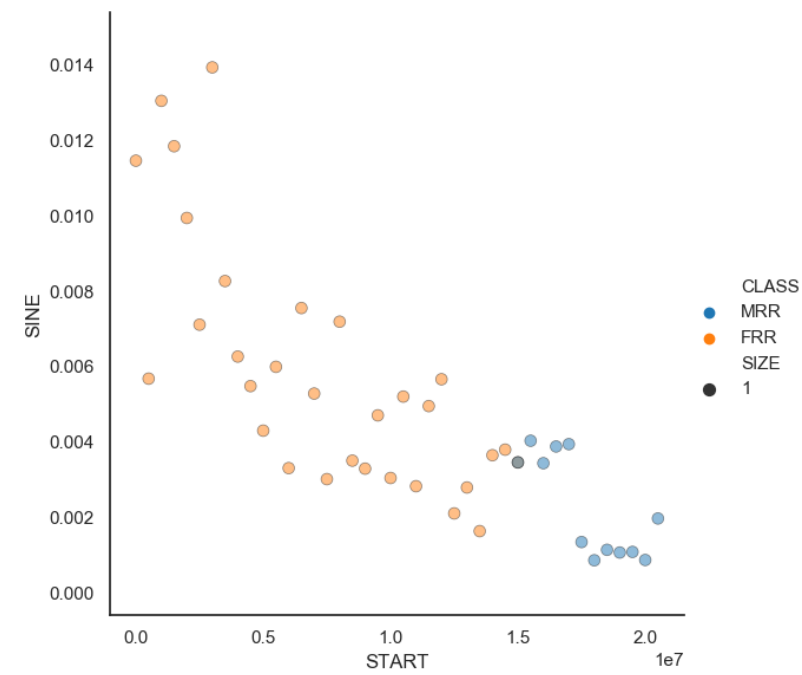

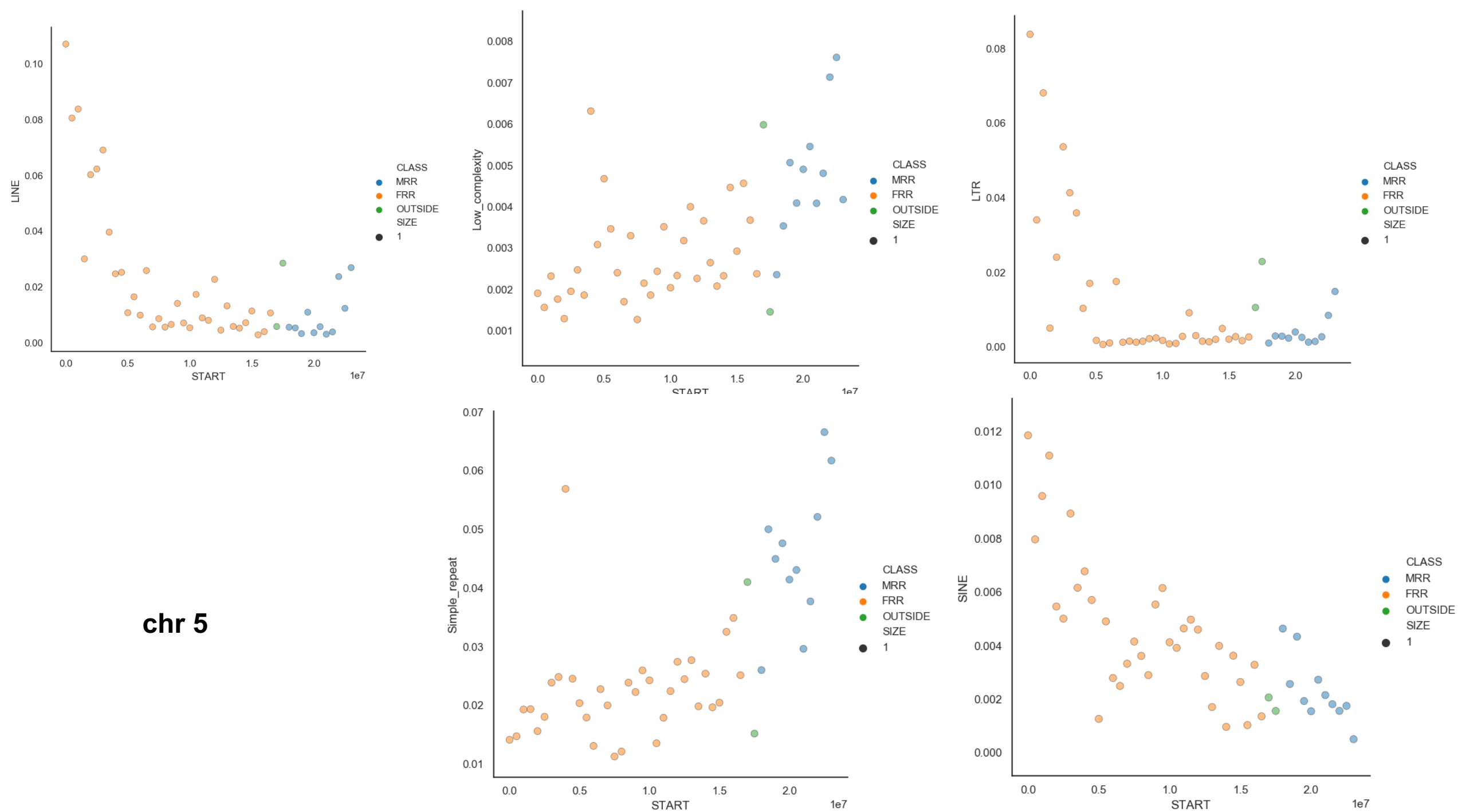

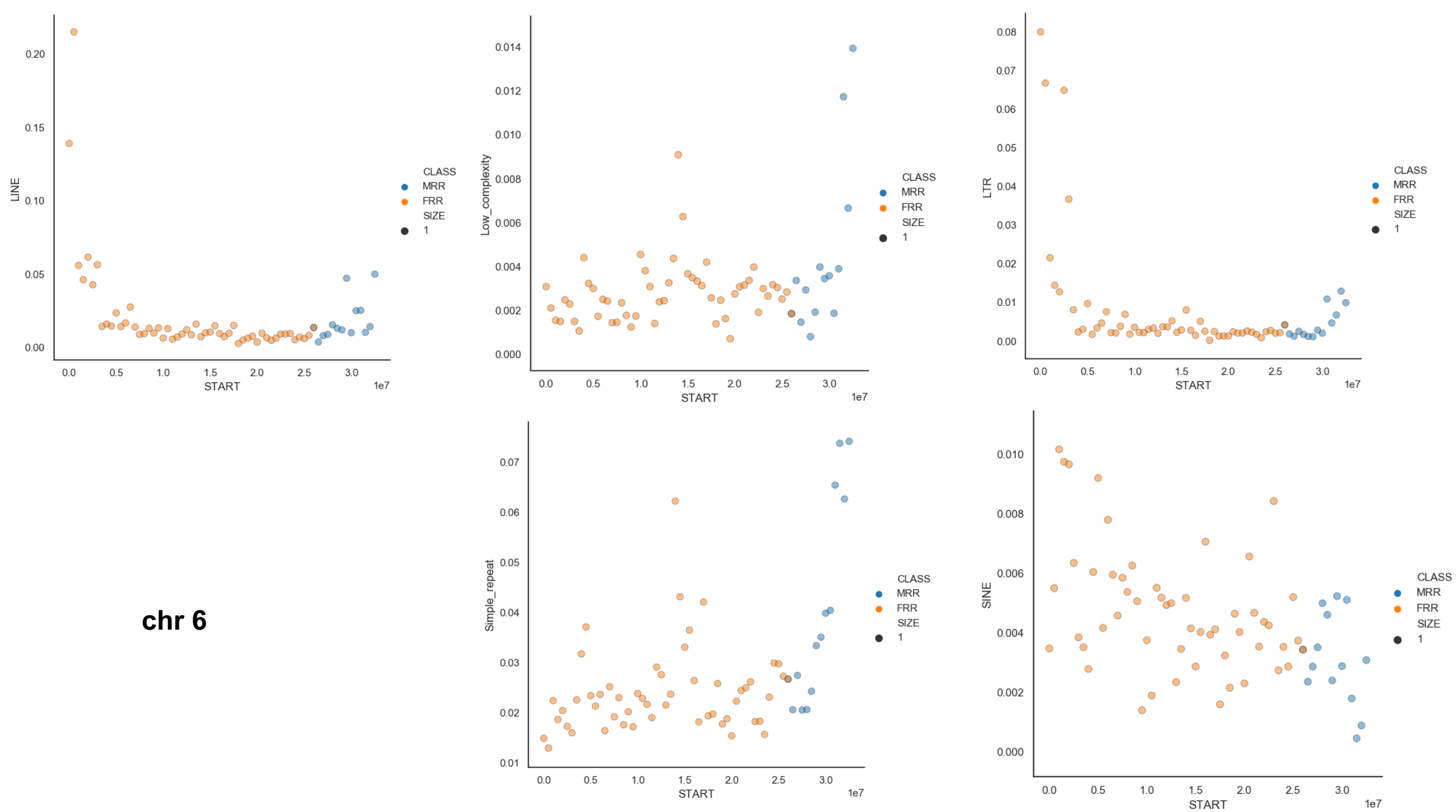

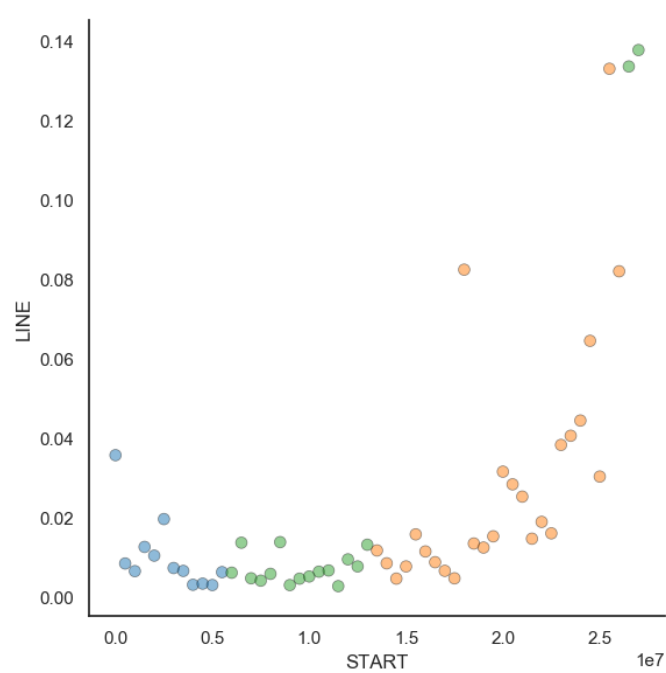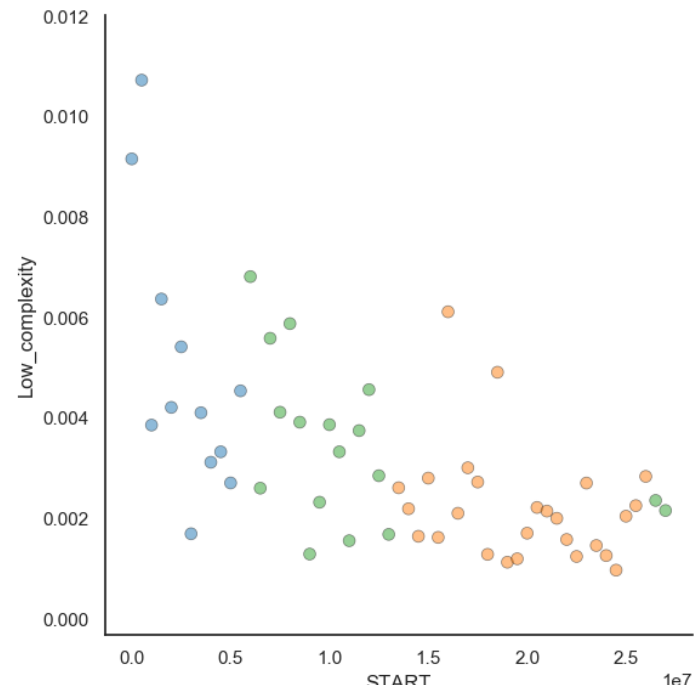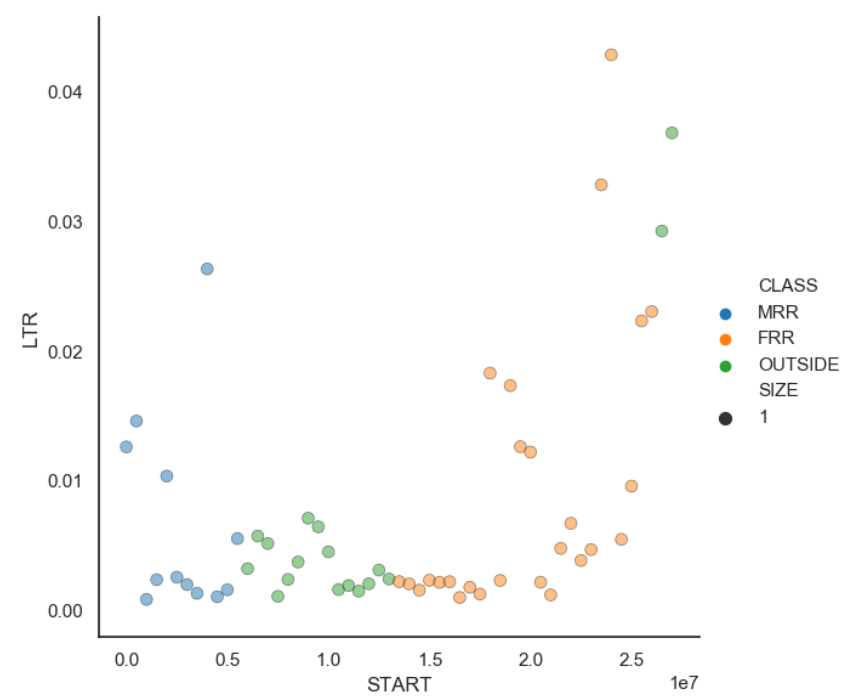

chr 7

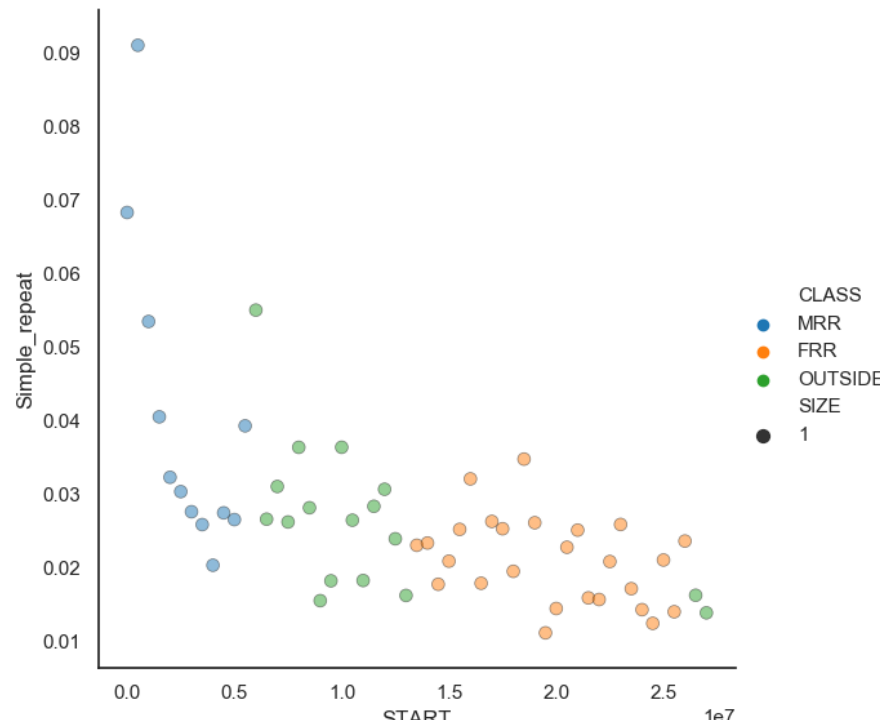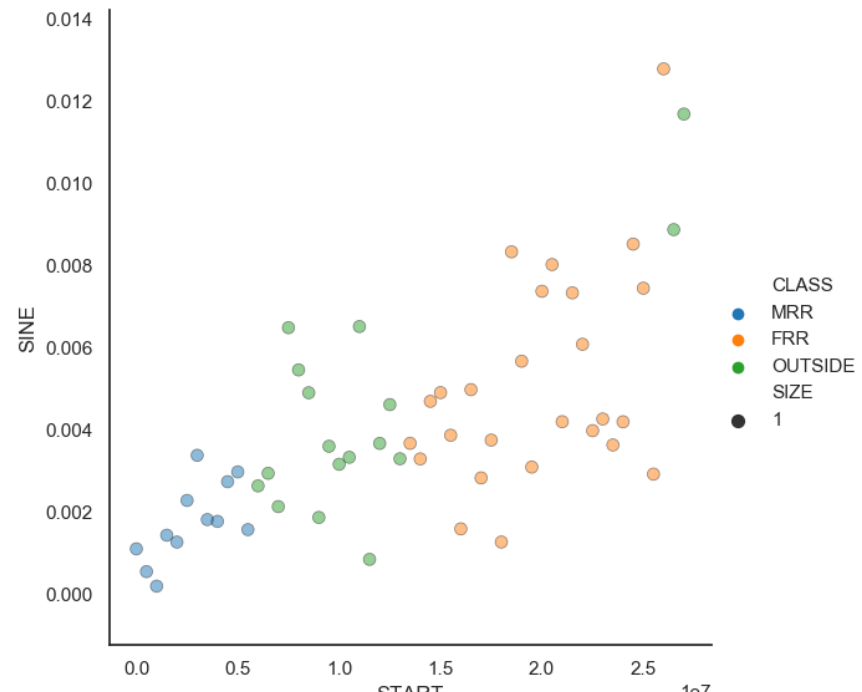

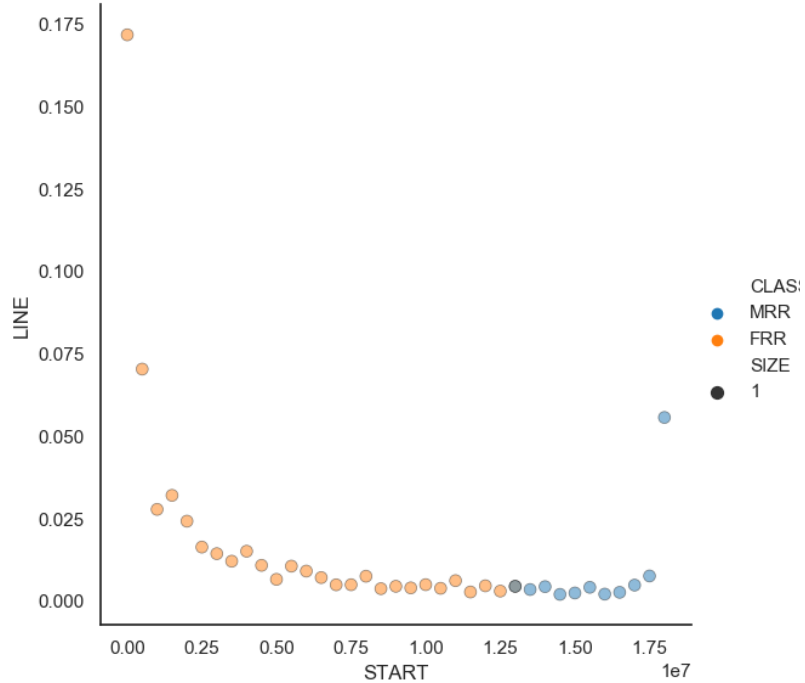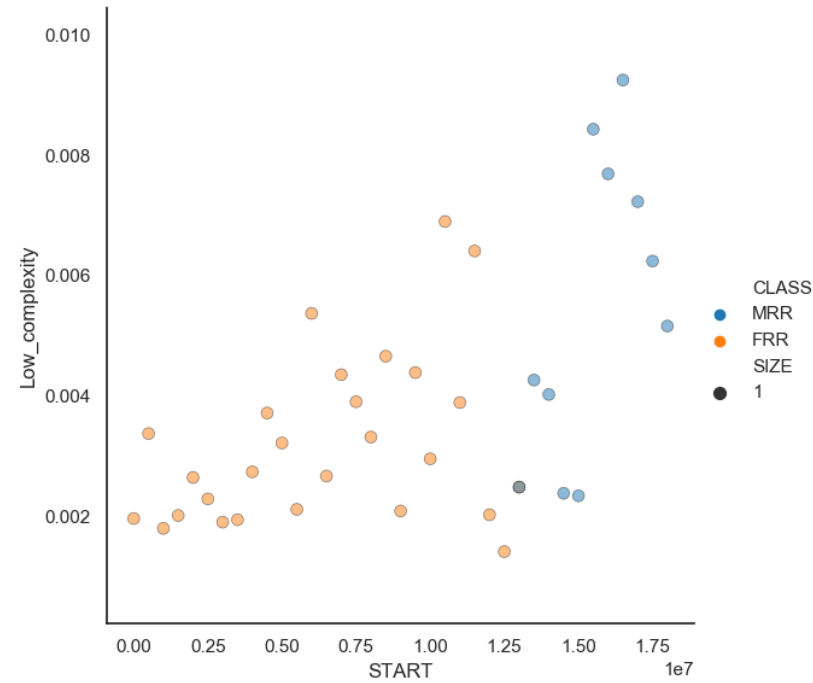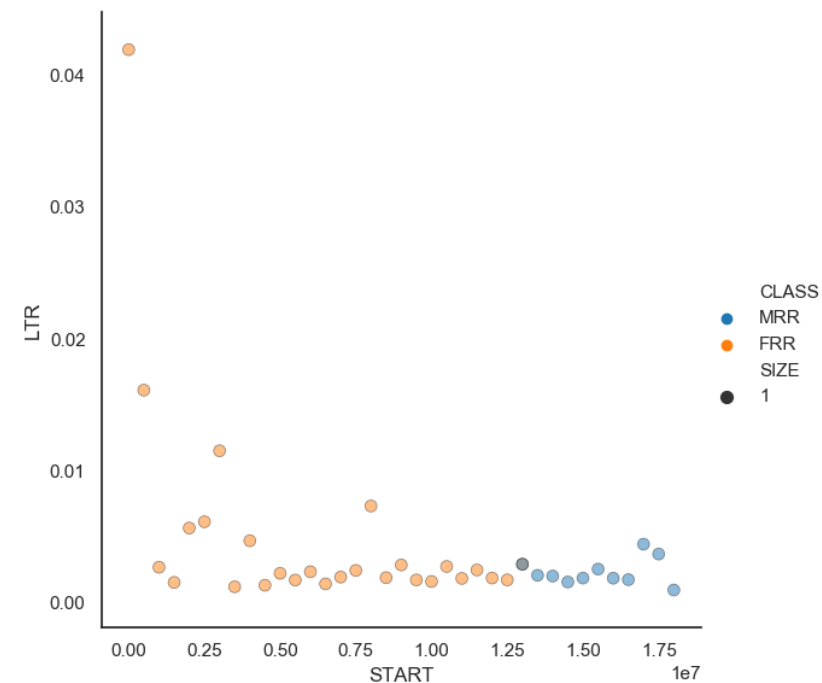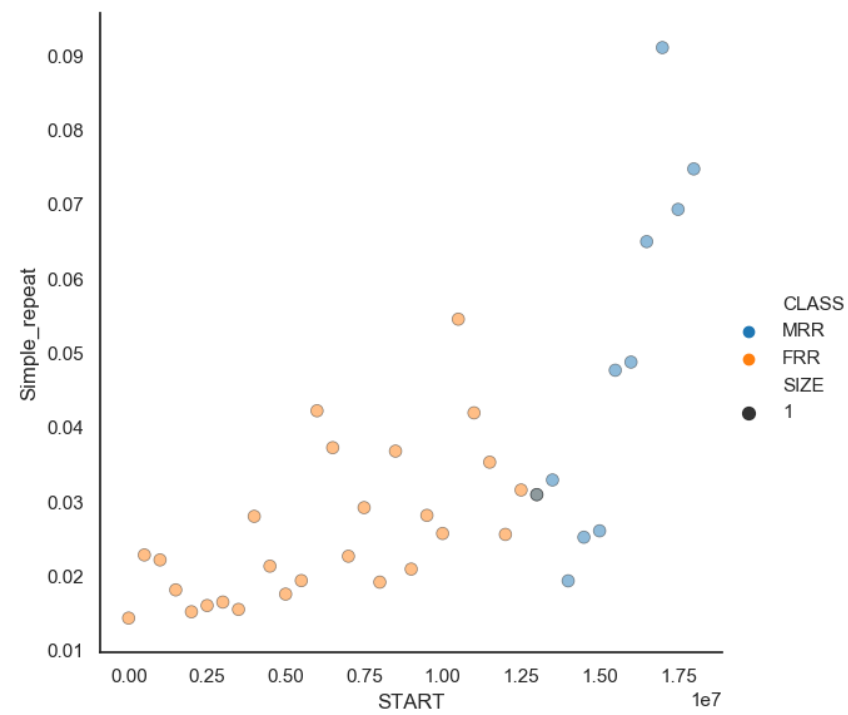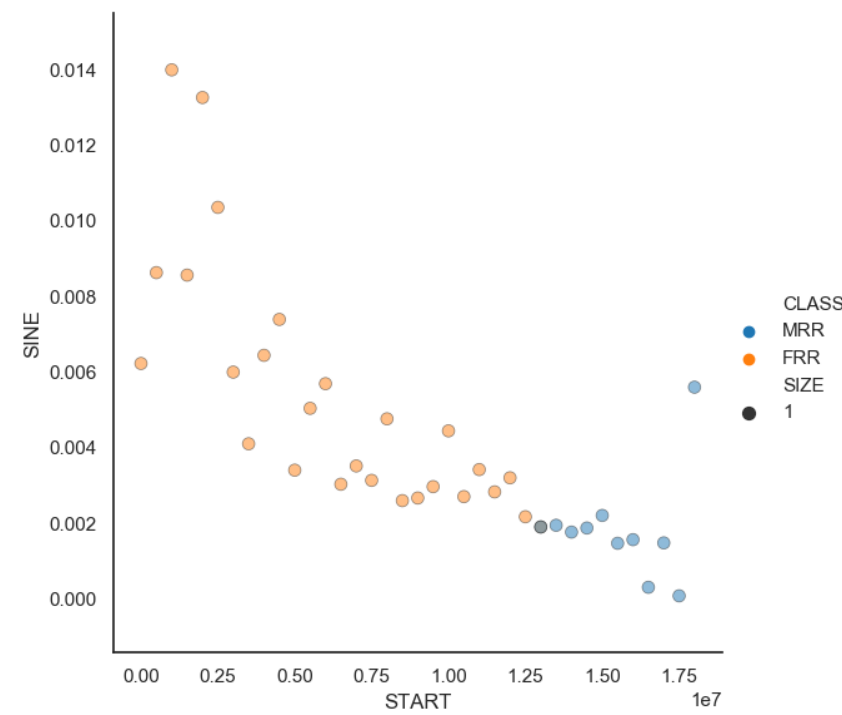

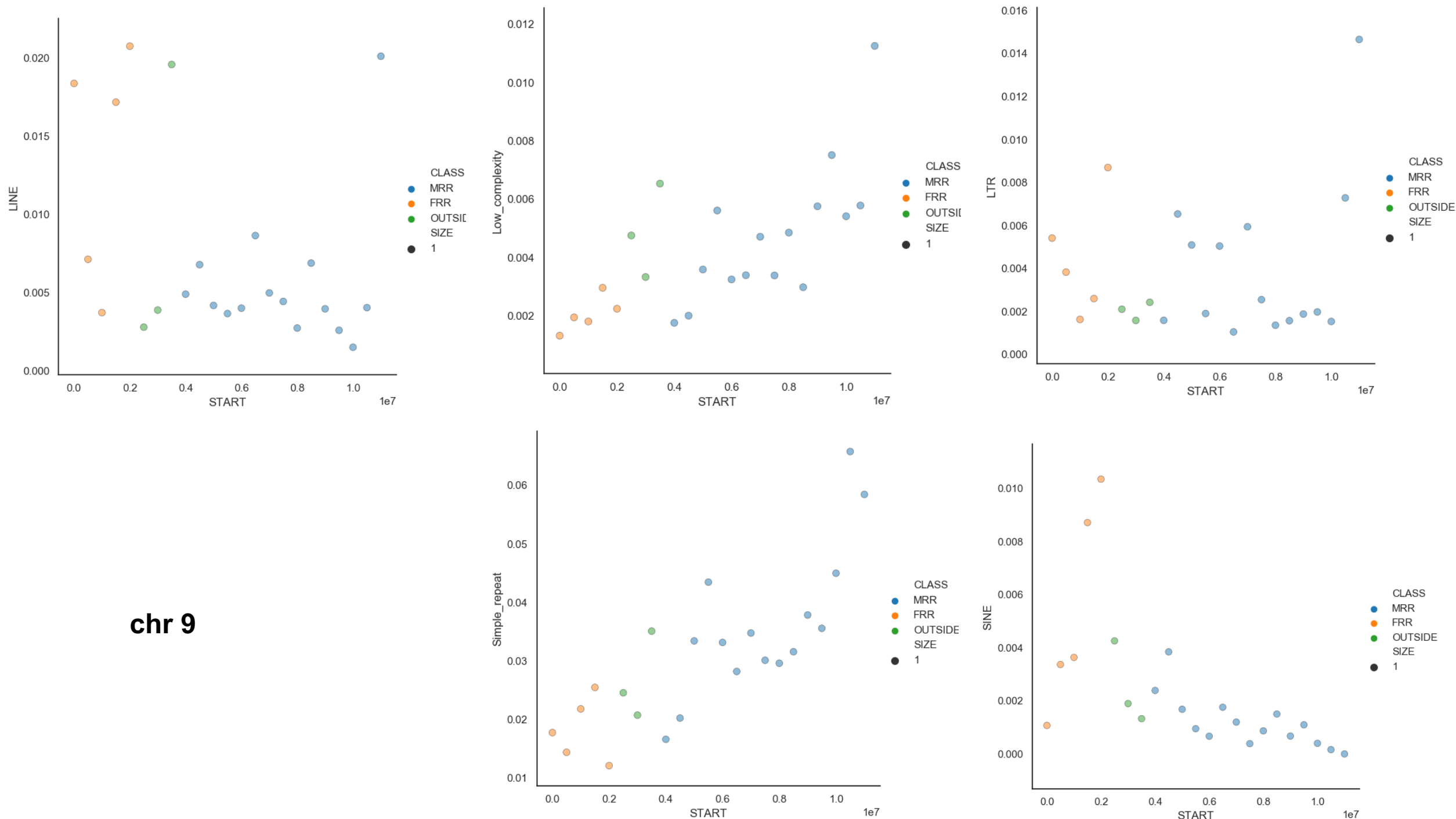

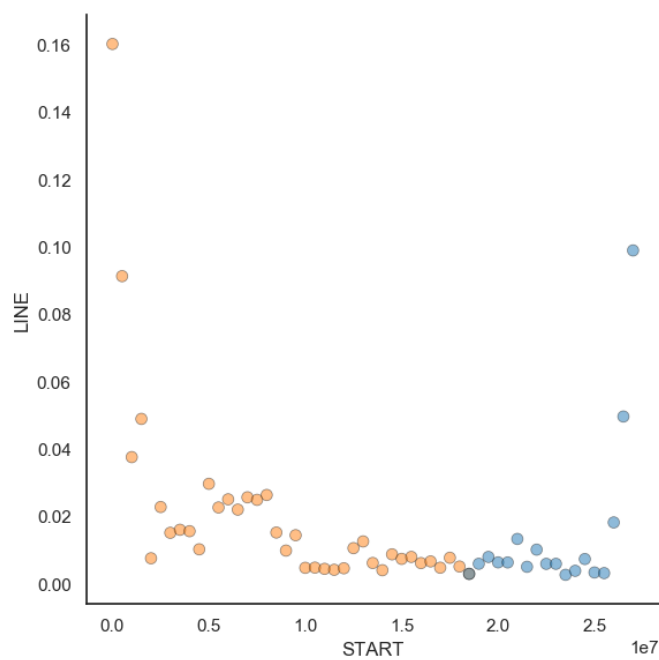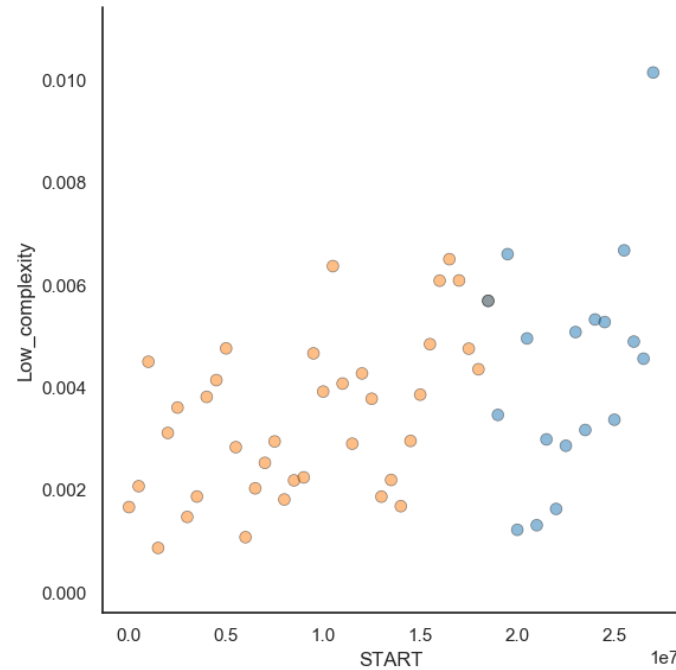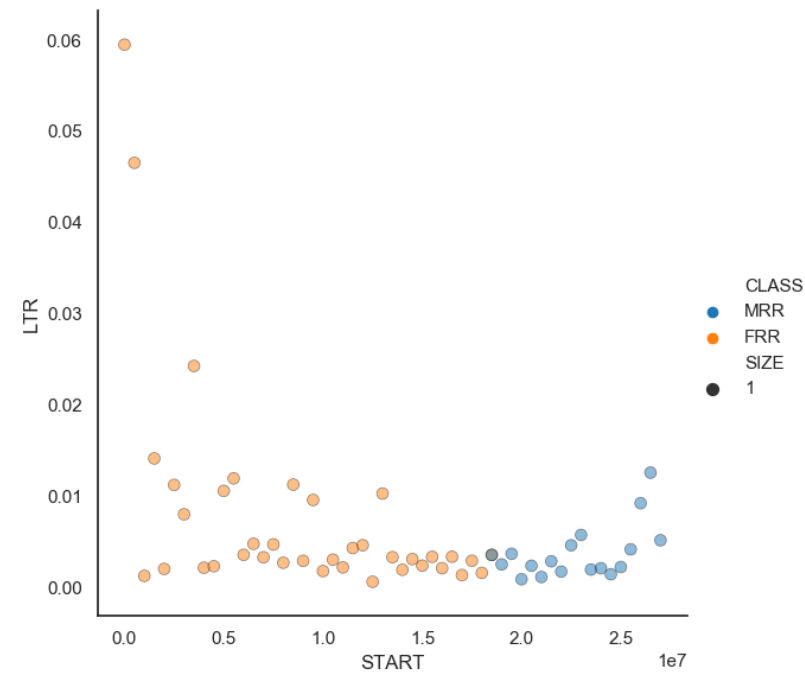

chr 10

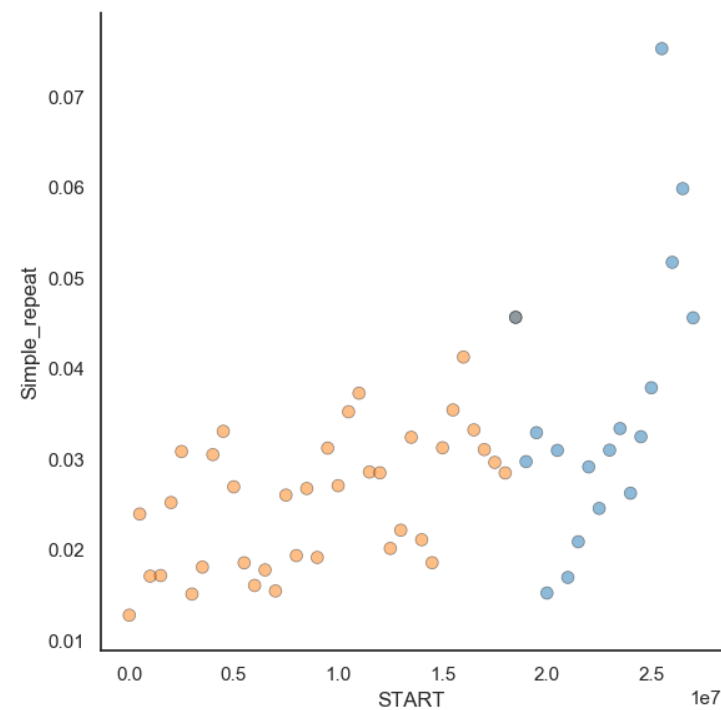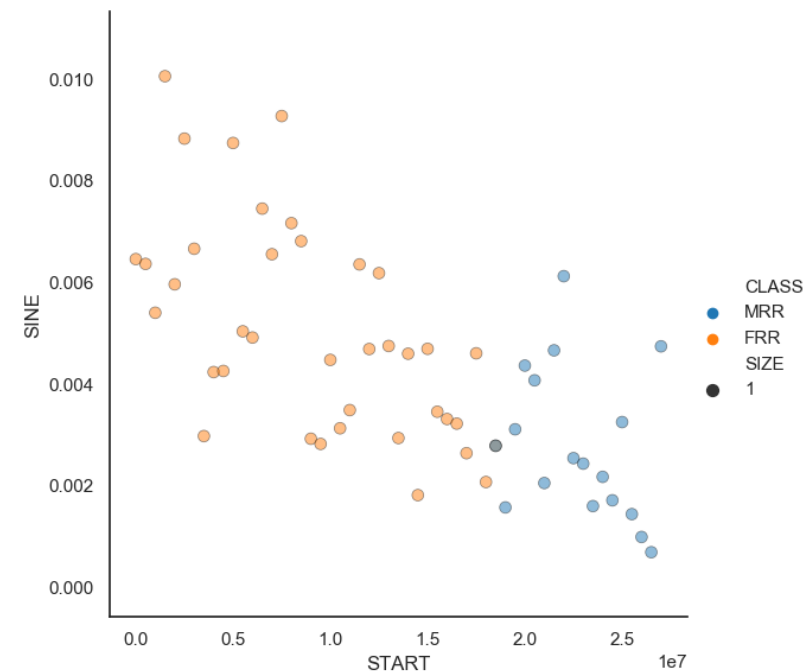

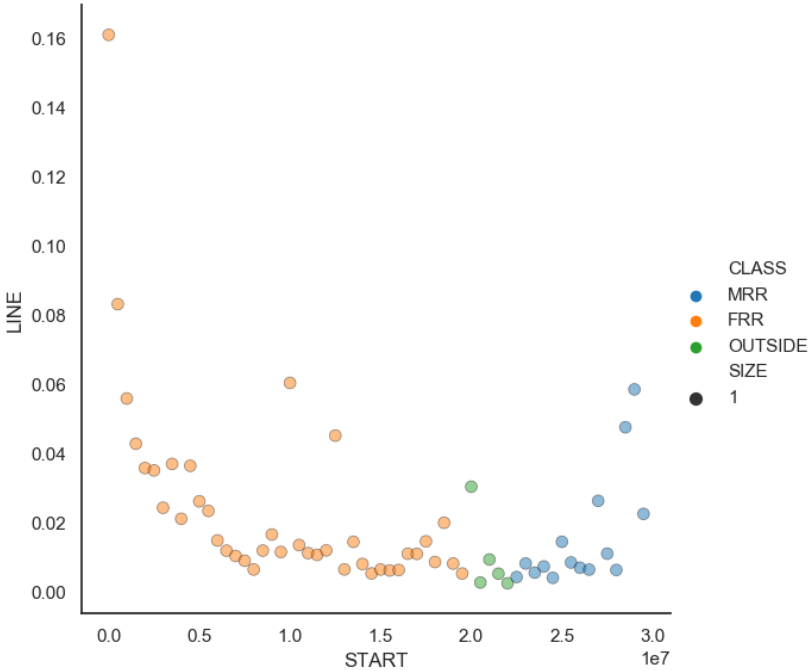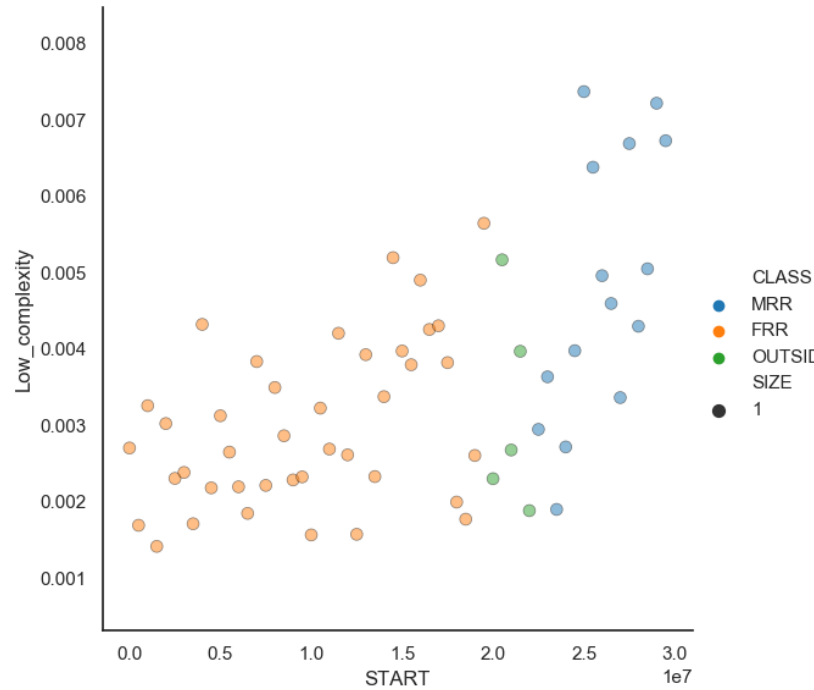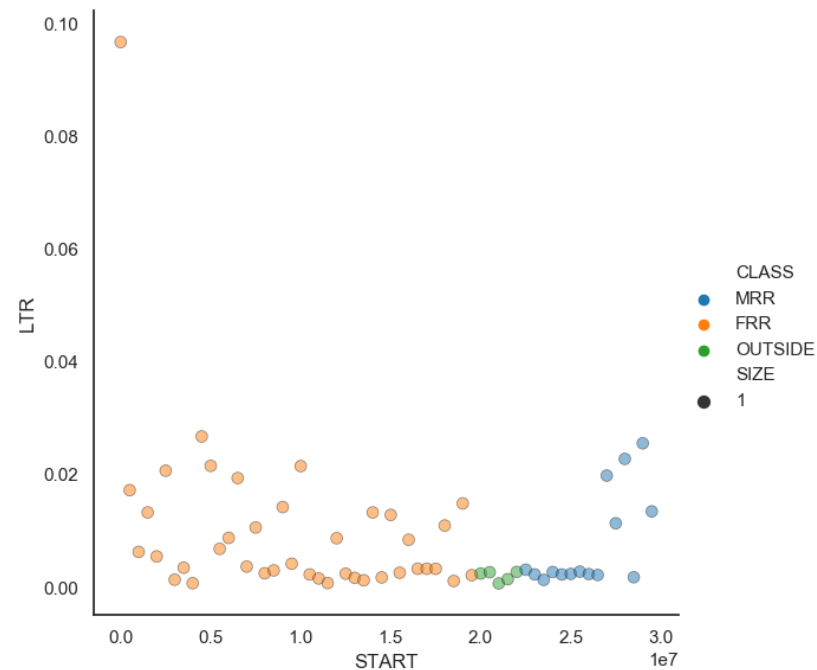

chr 11

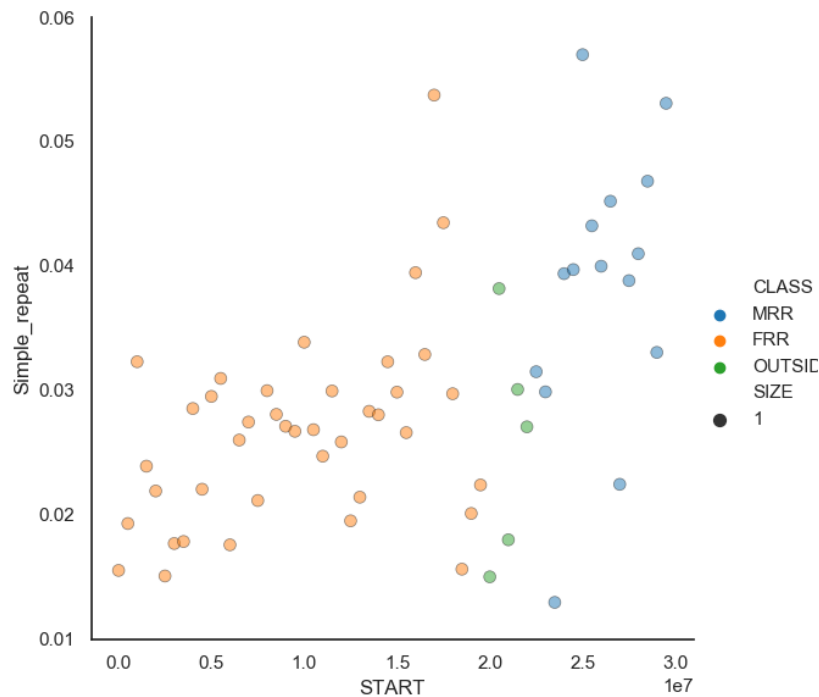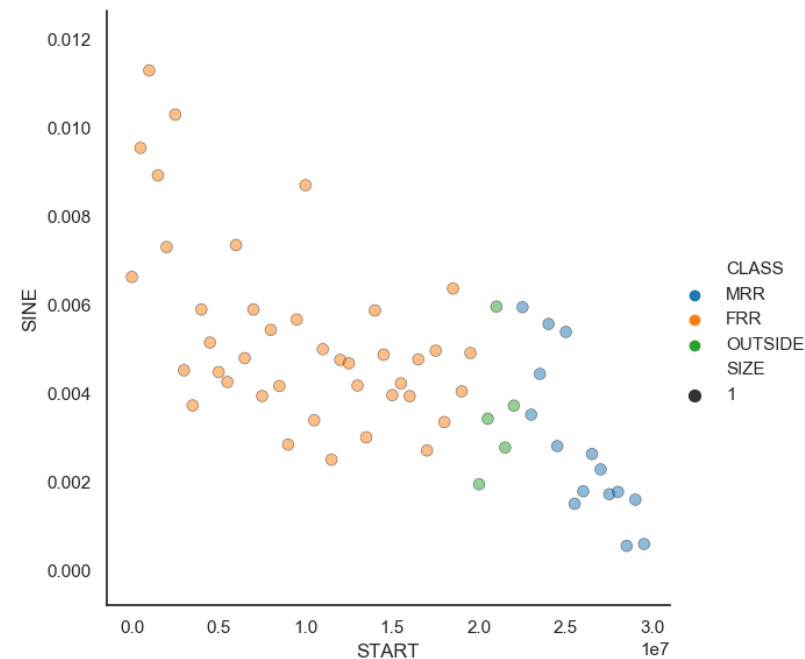

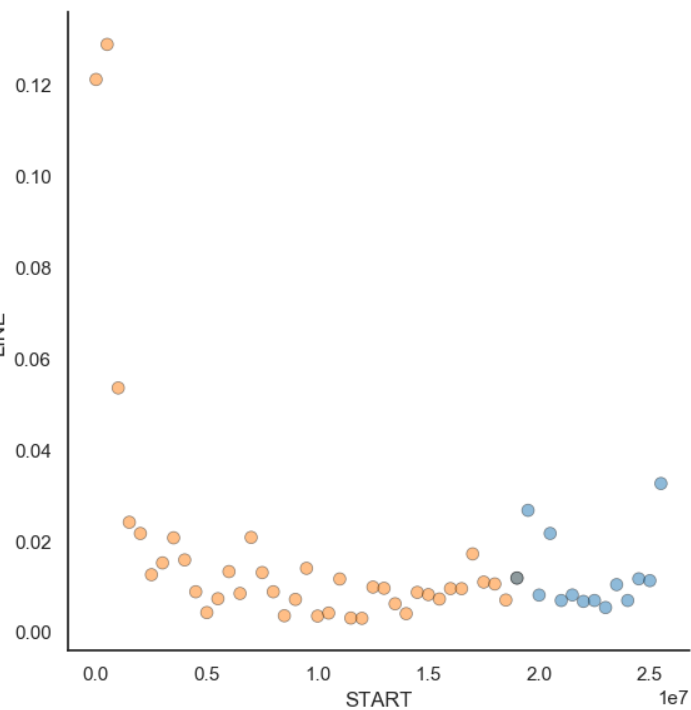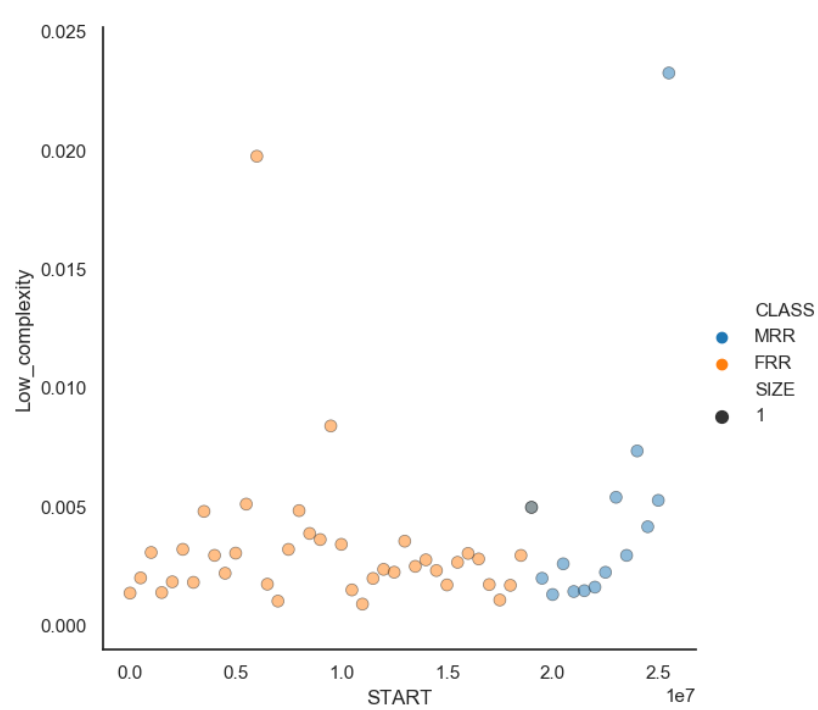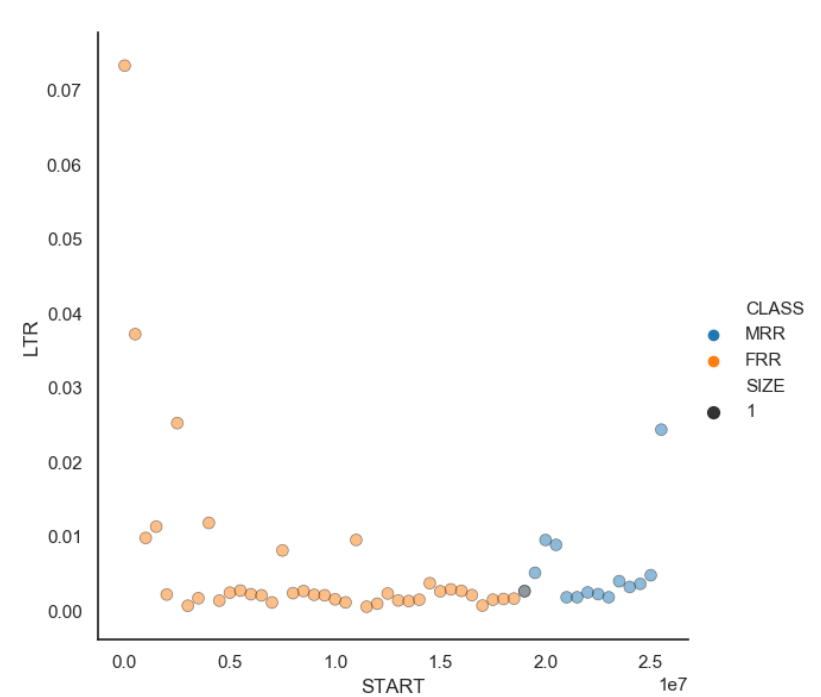

chr 12

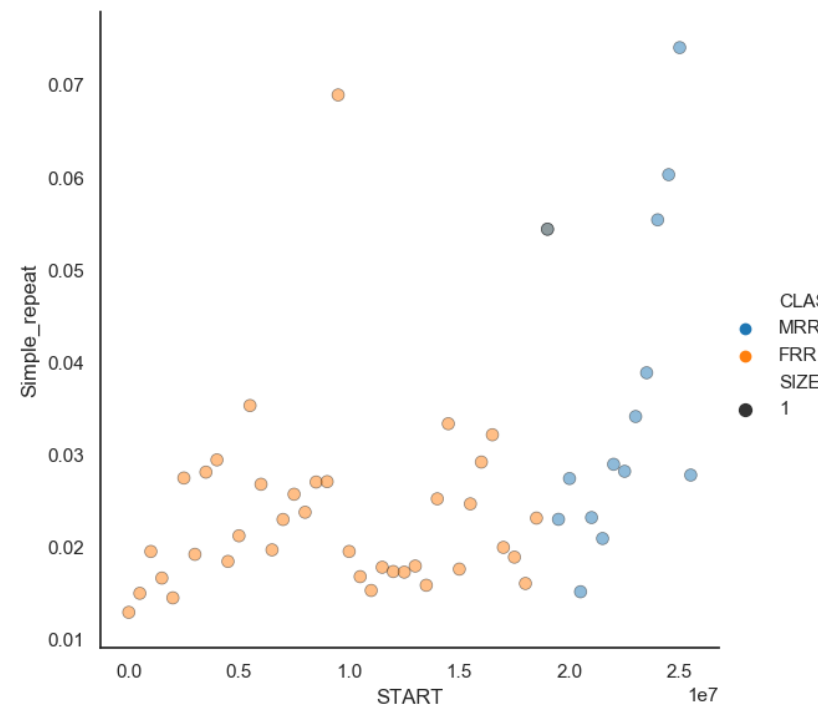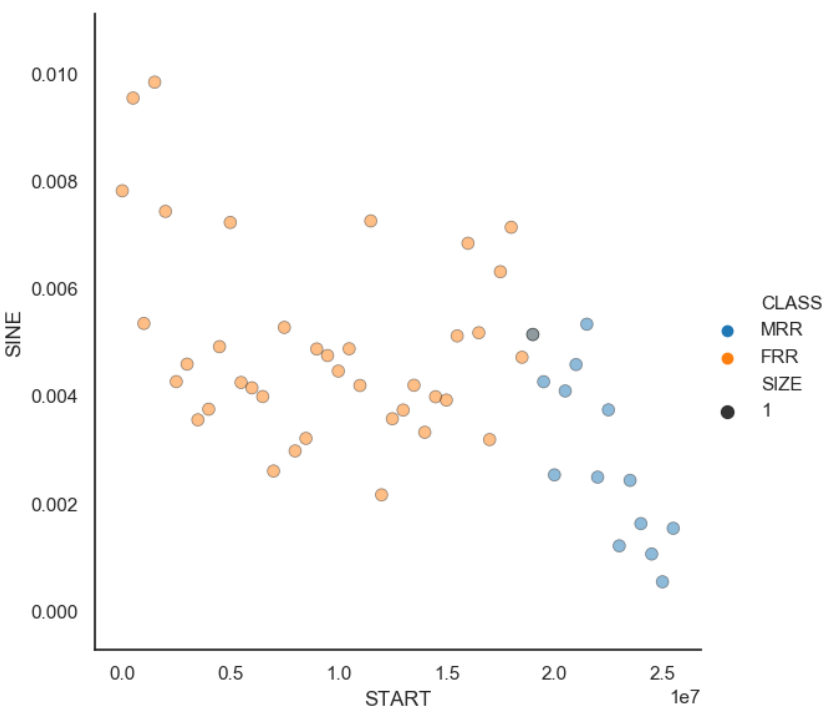

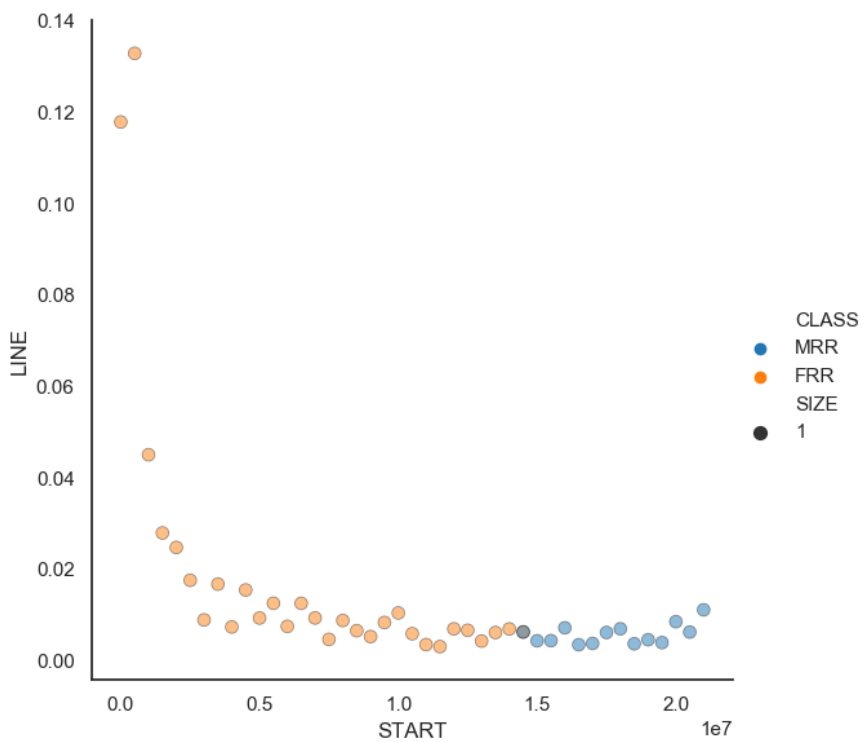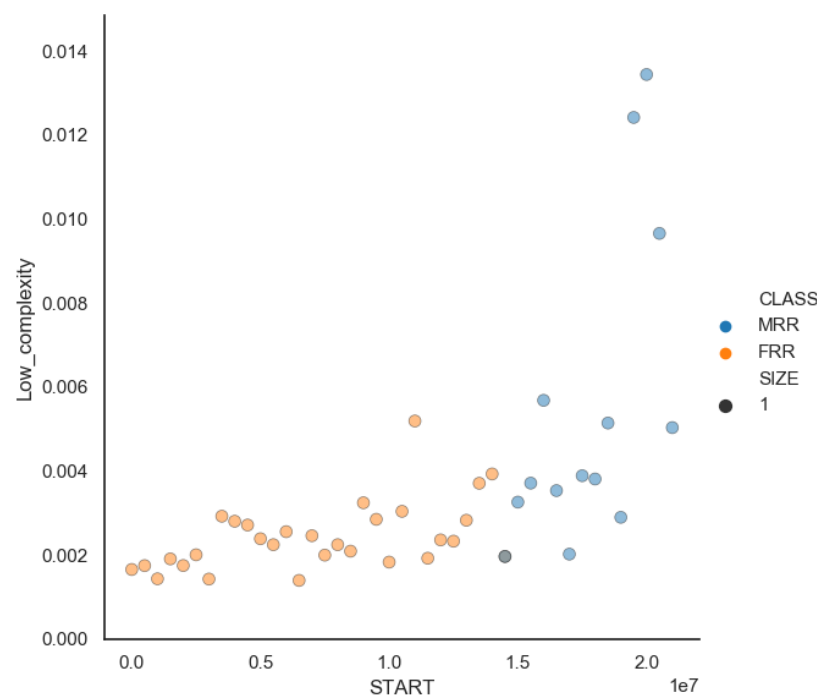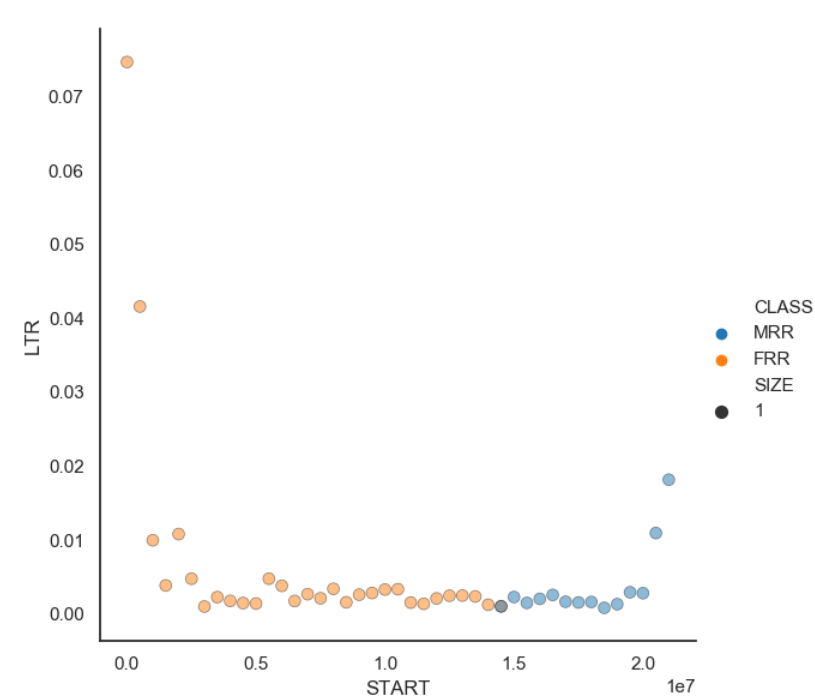

chr 13

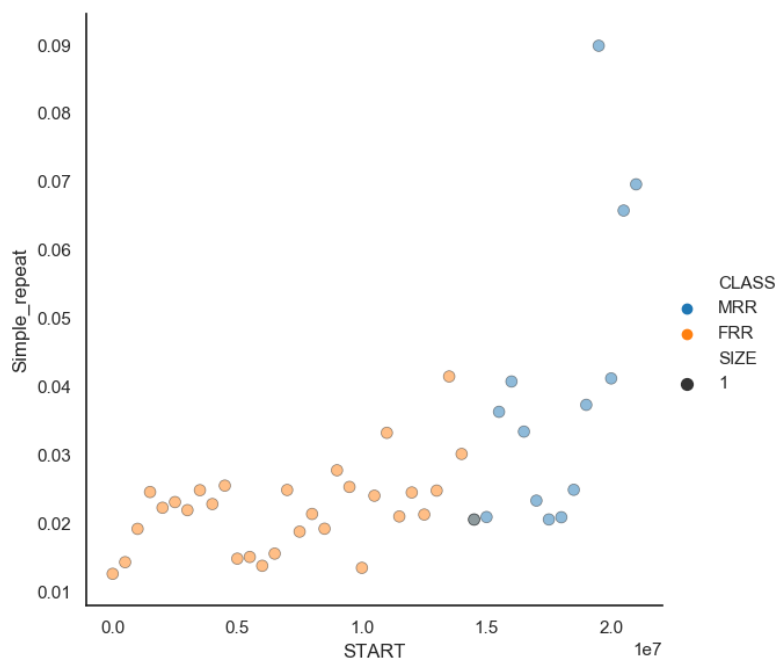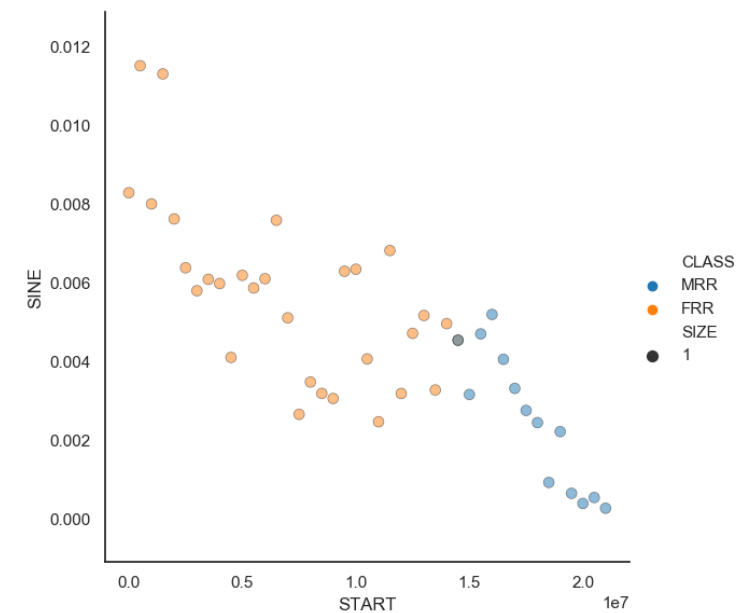

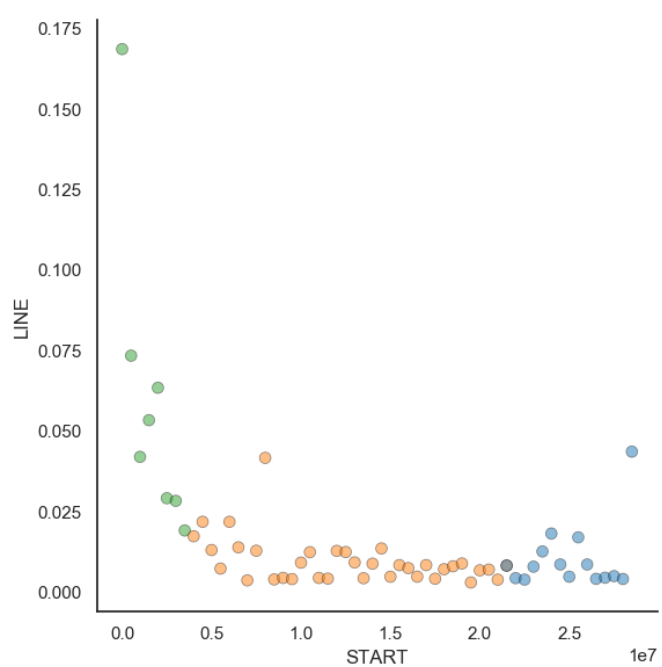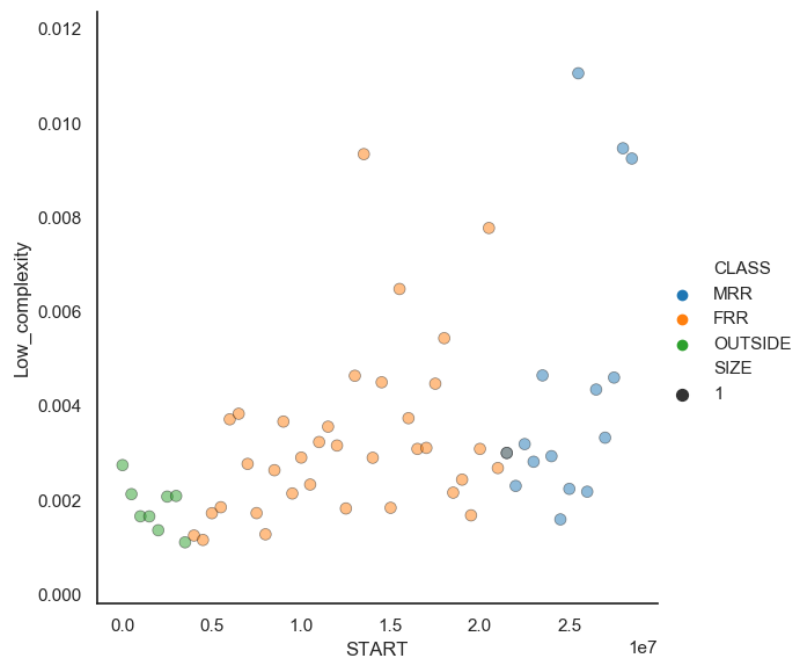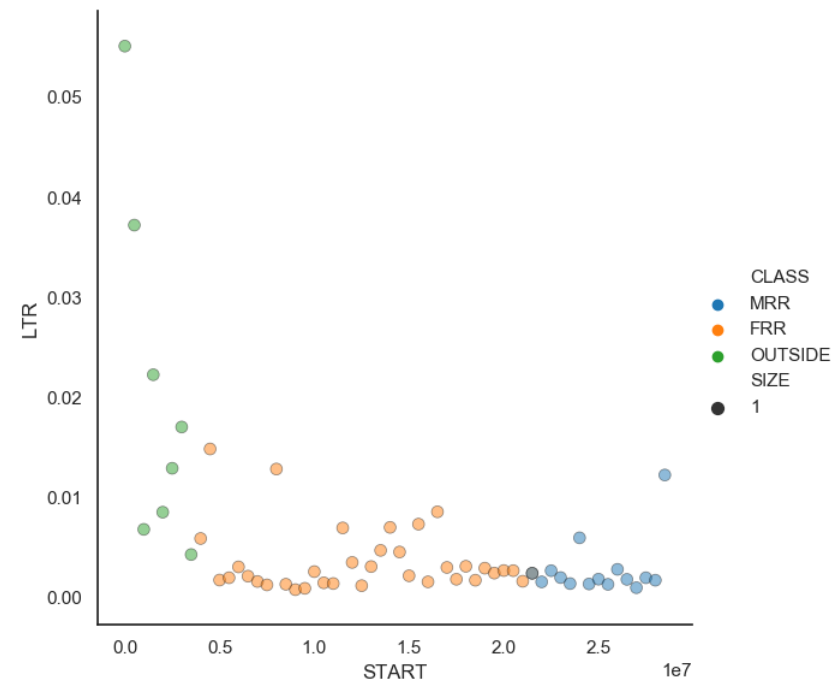

chr 14

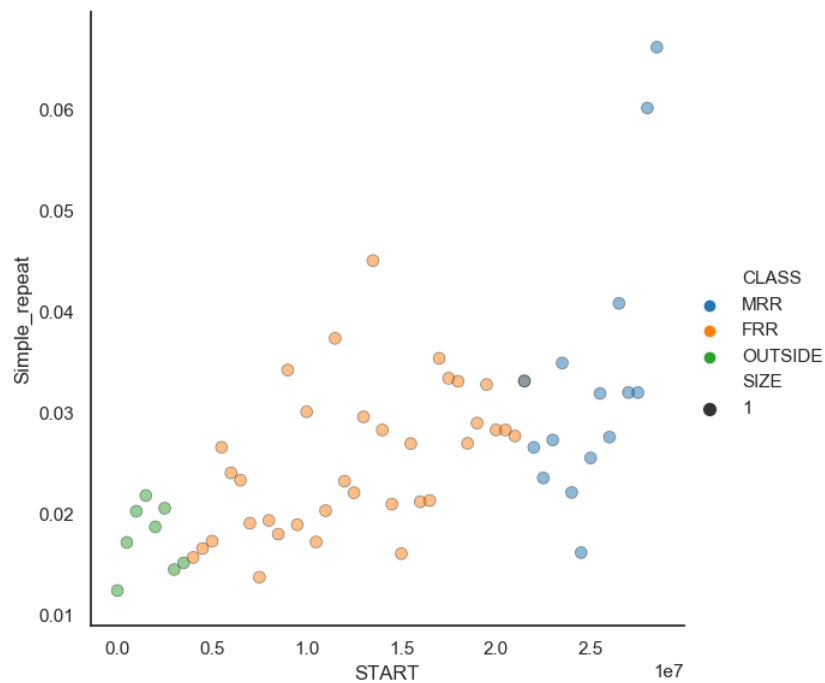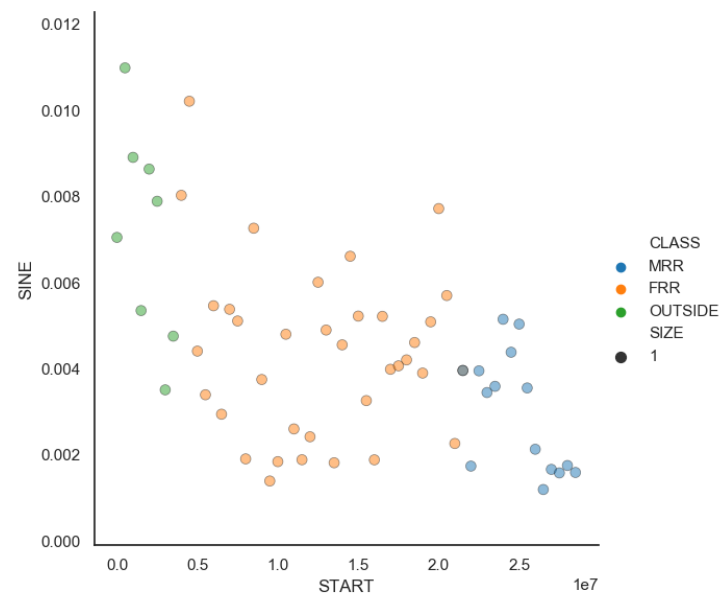

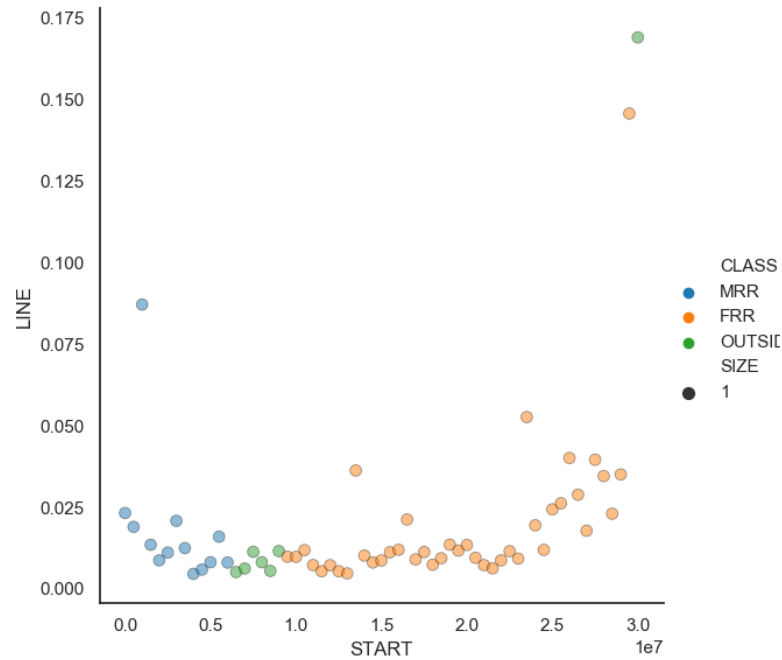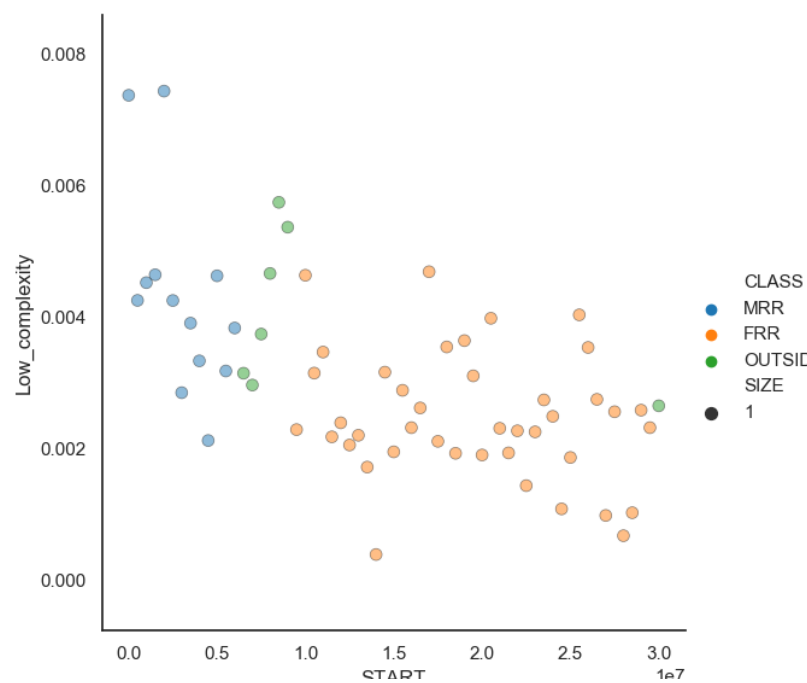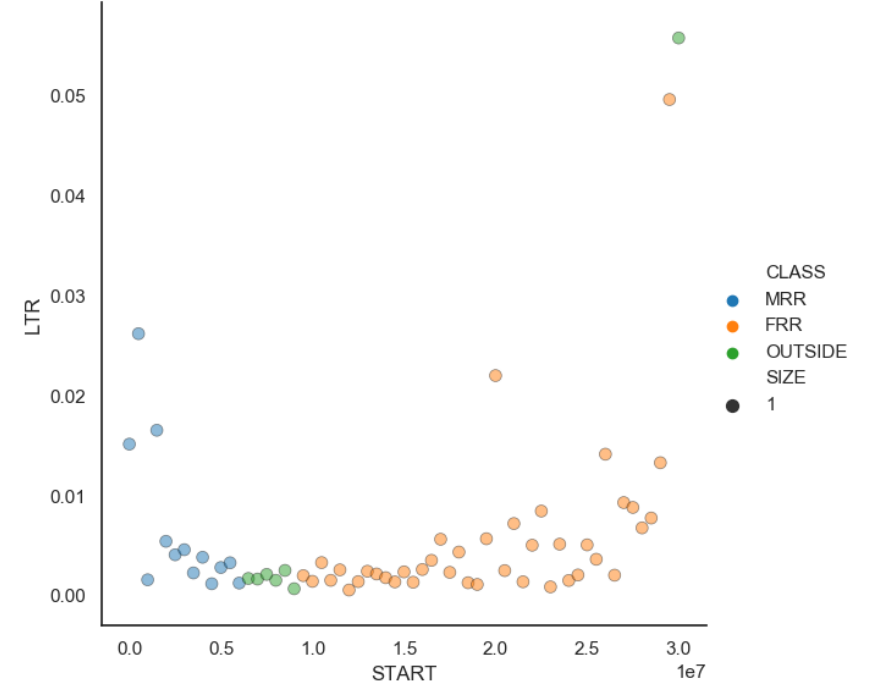

chr 15

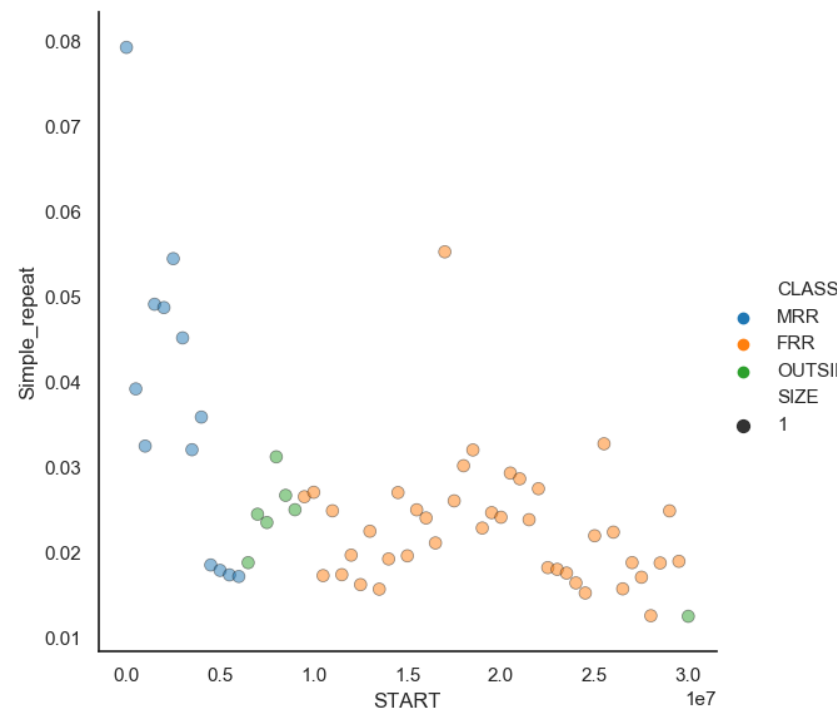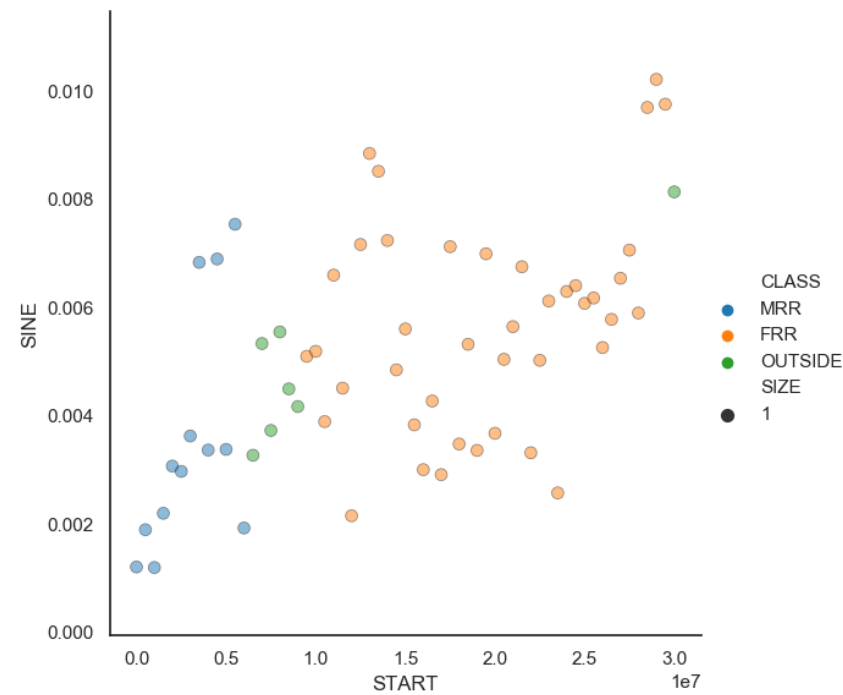

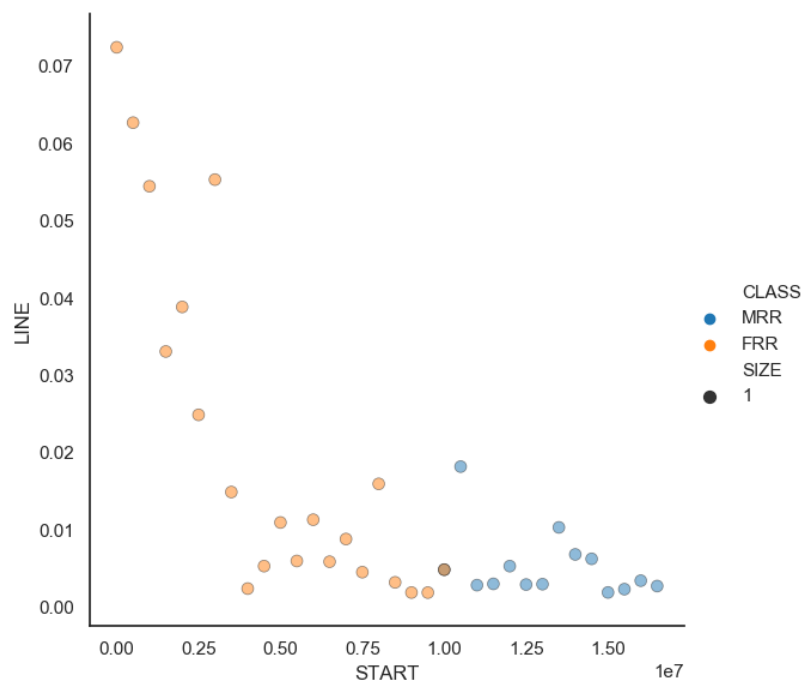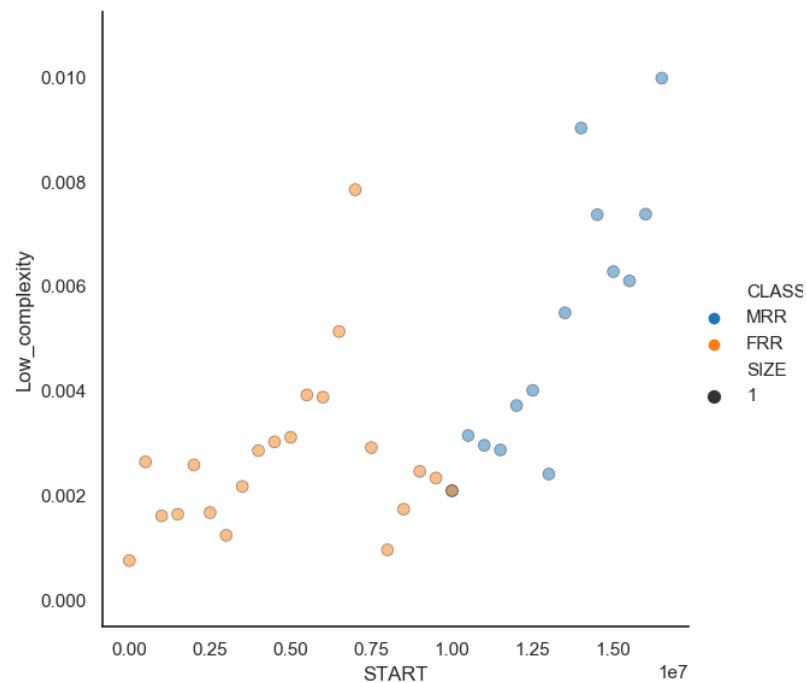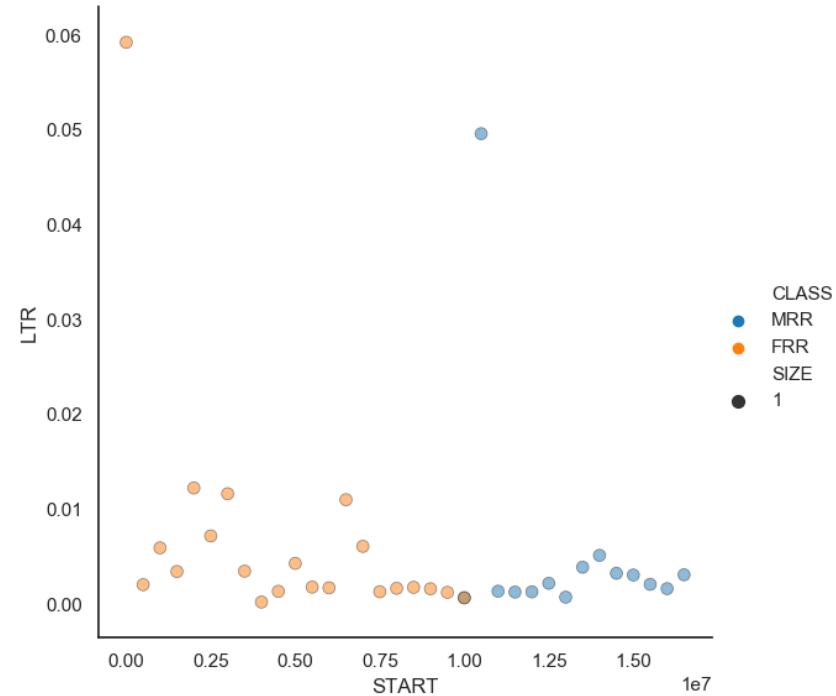

chr 16

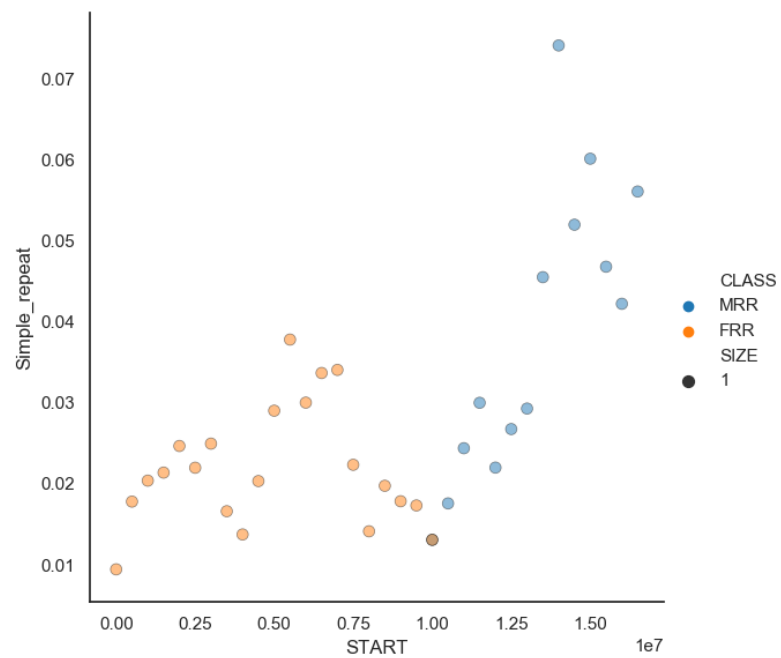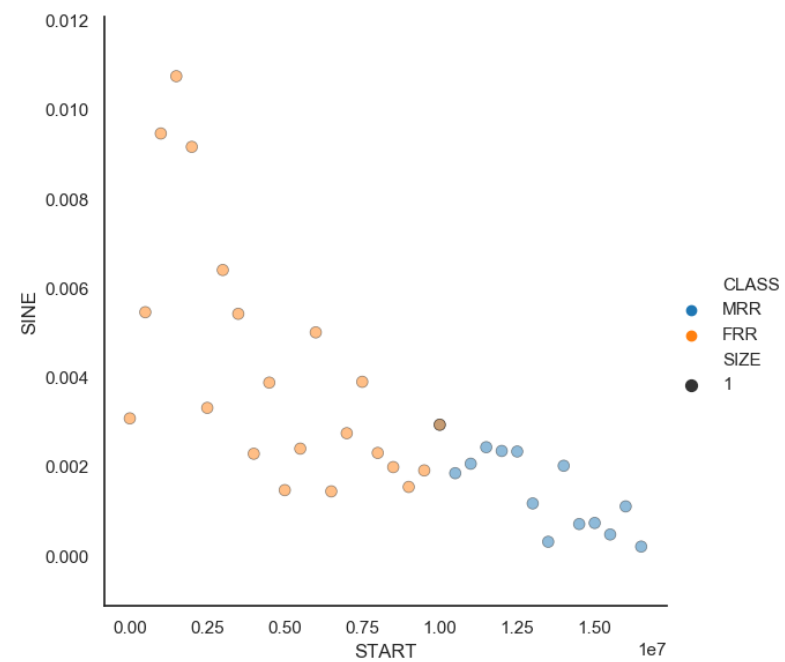

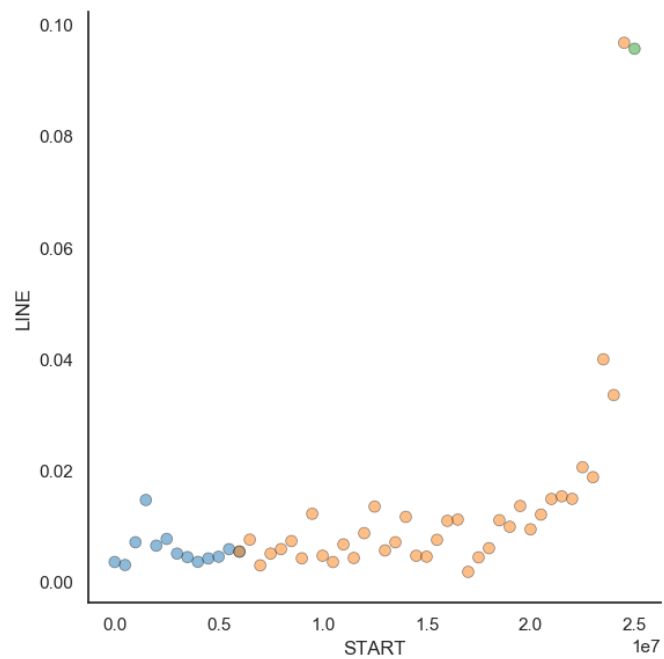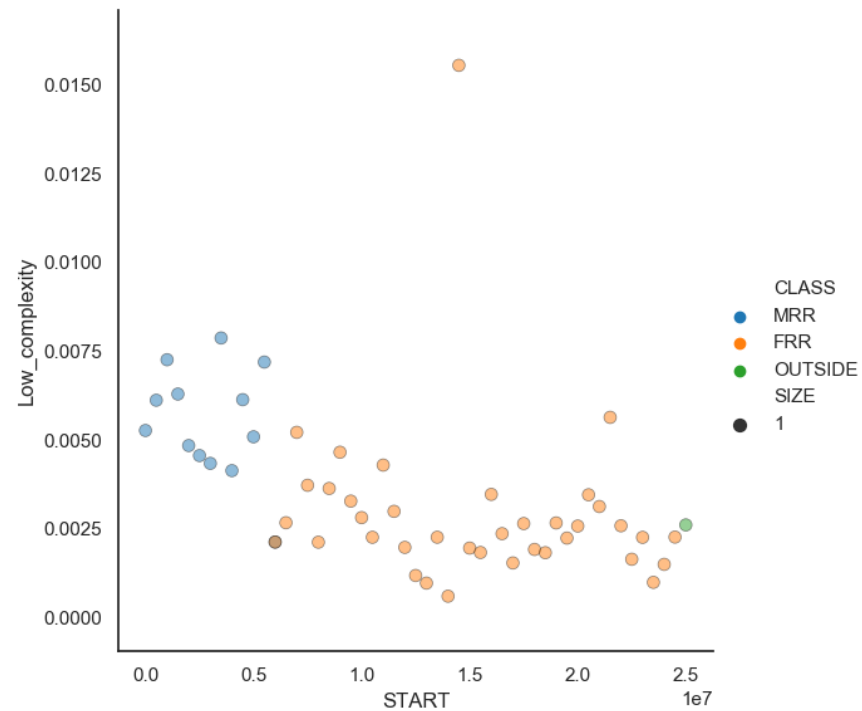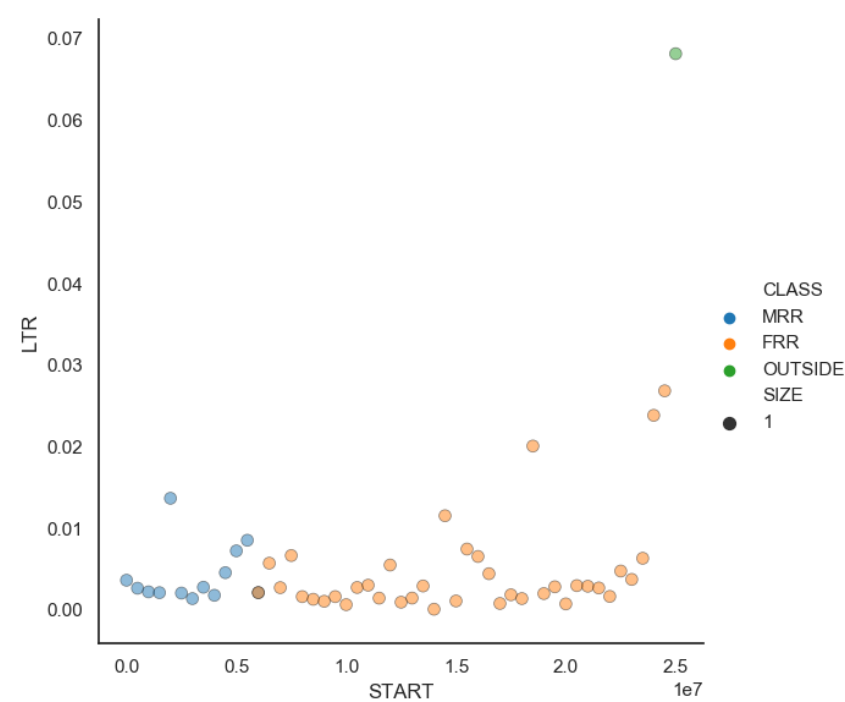

chr 17

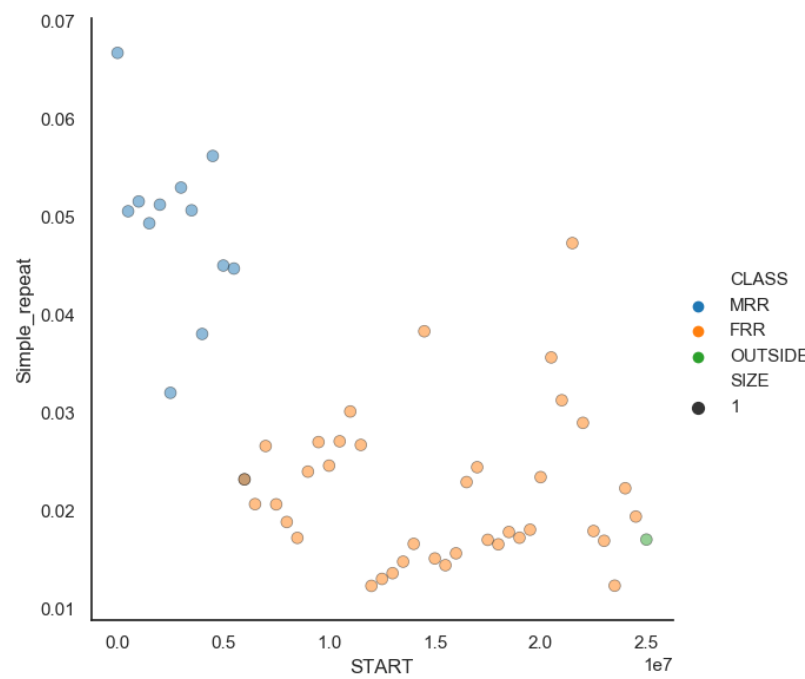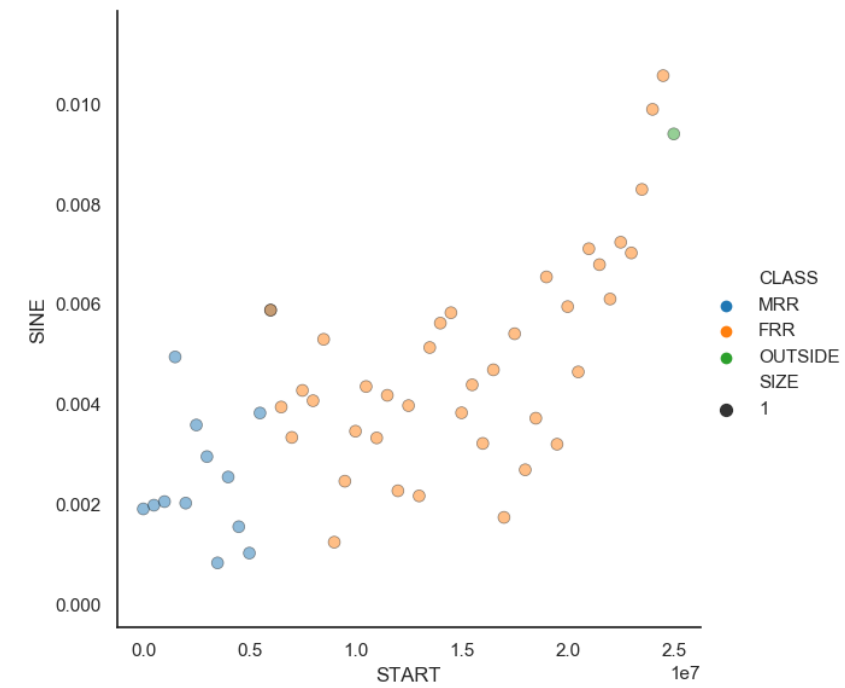

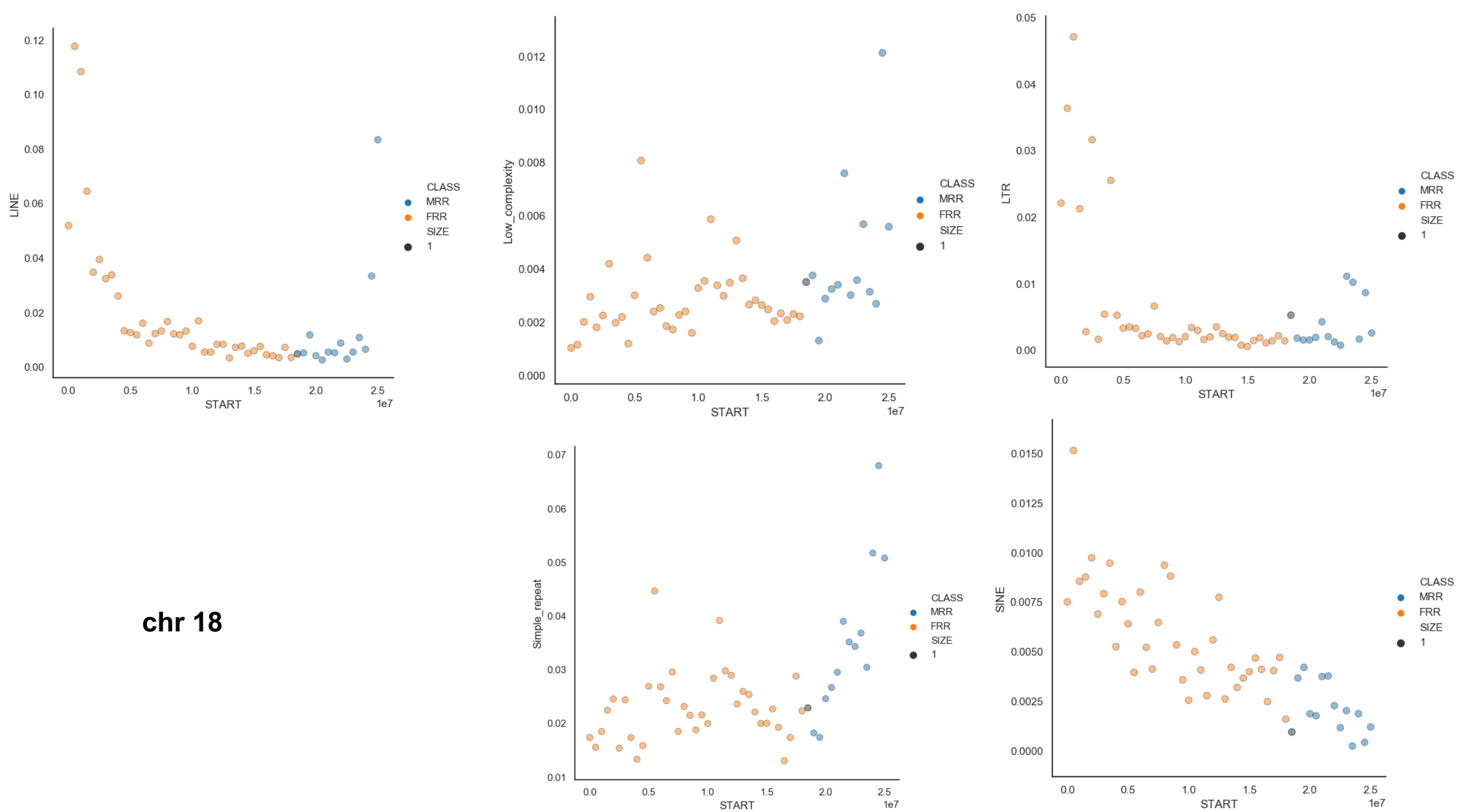

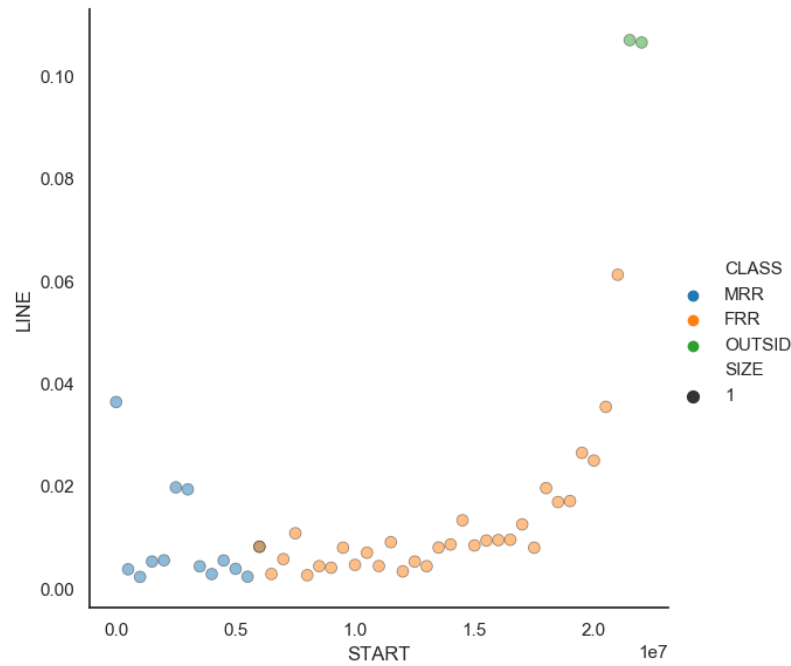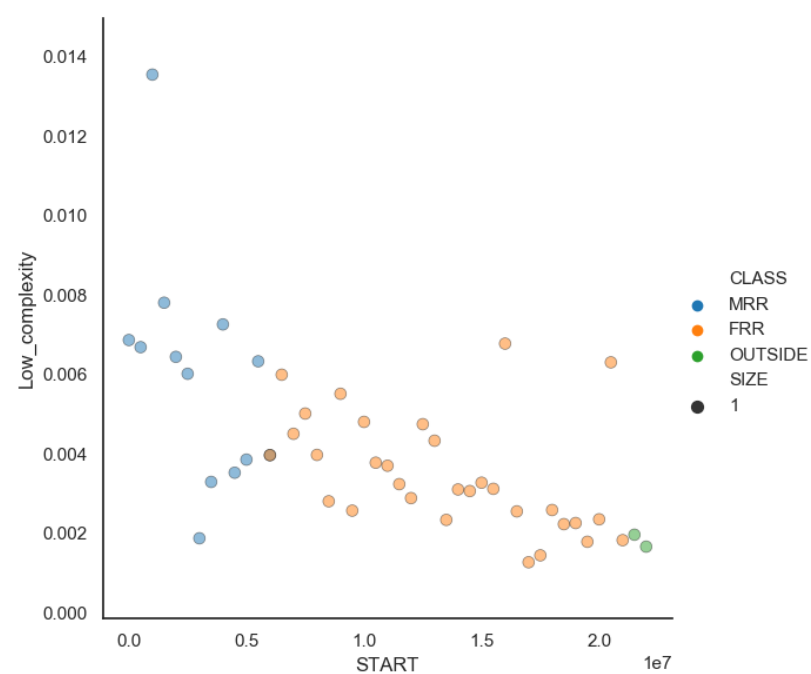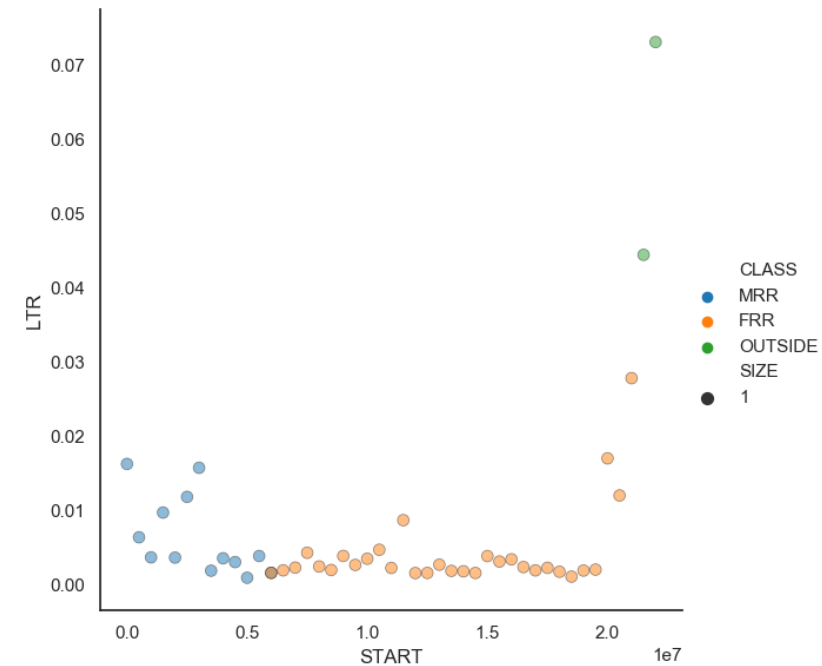

chr 19

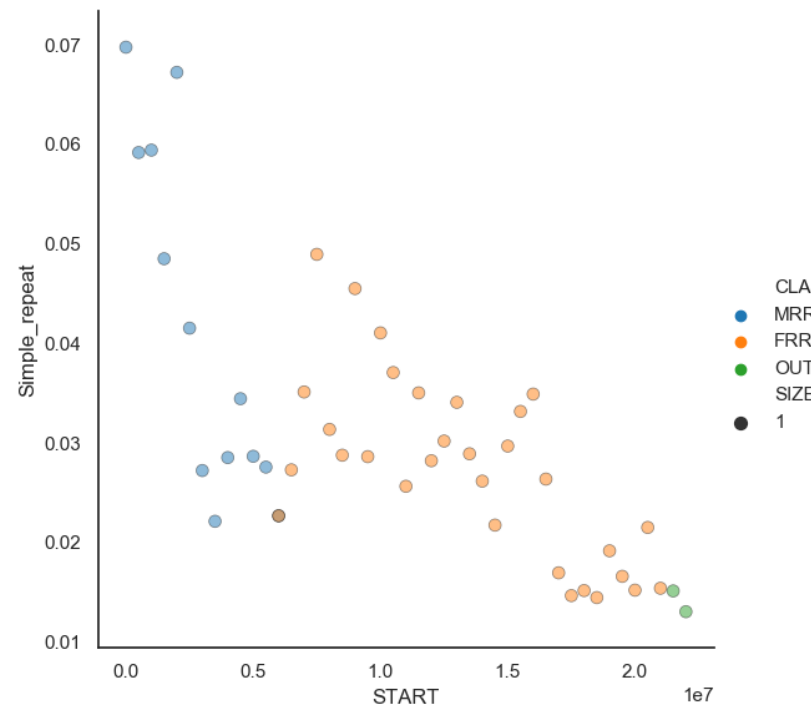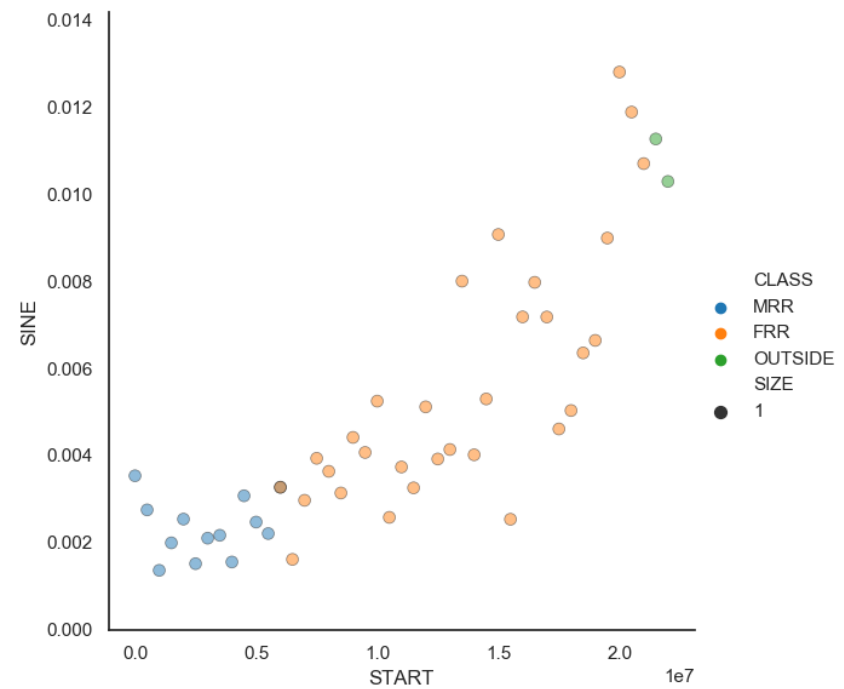

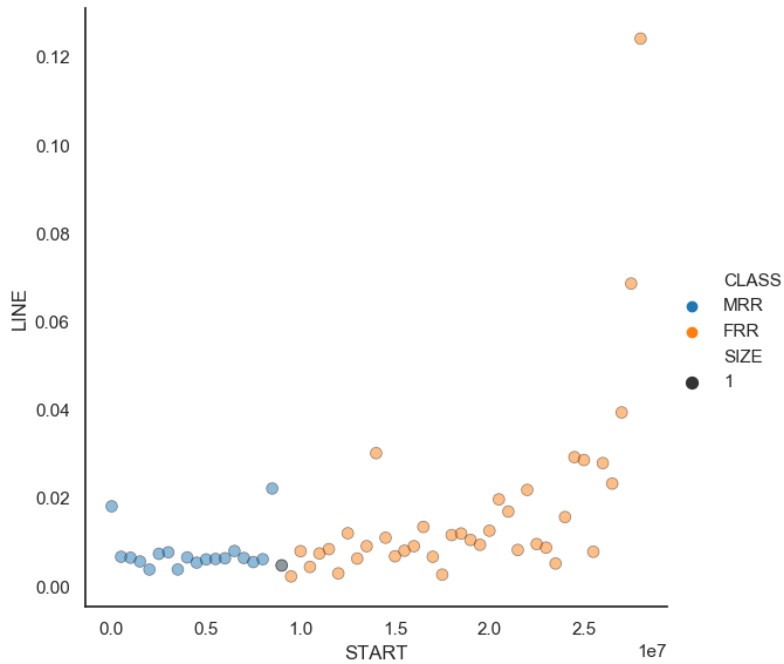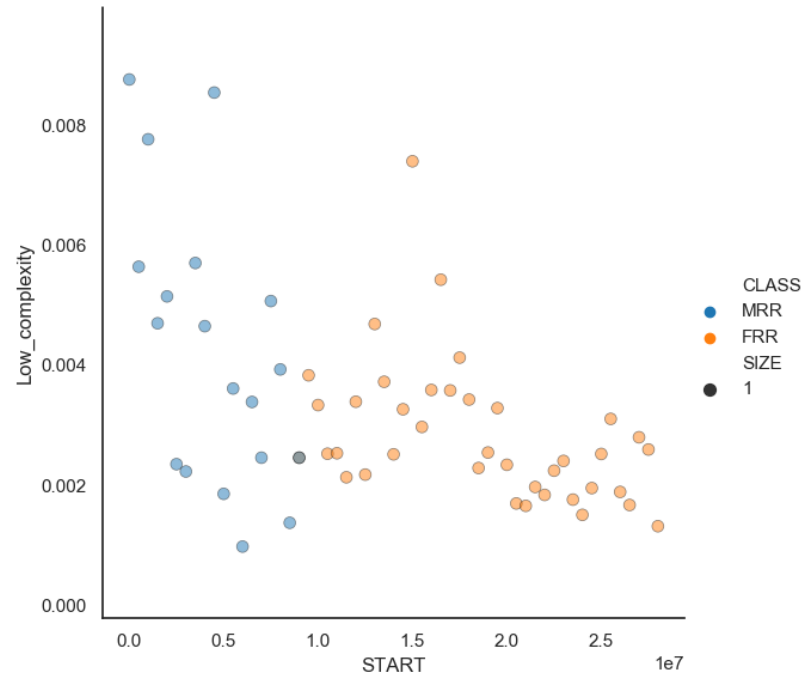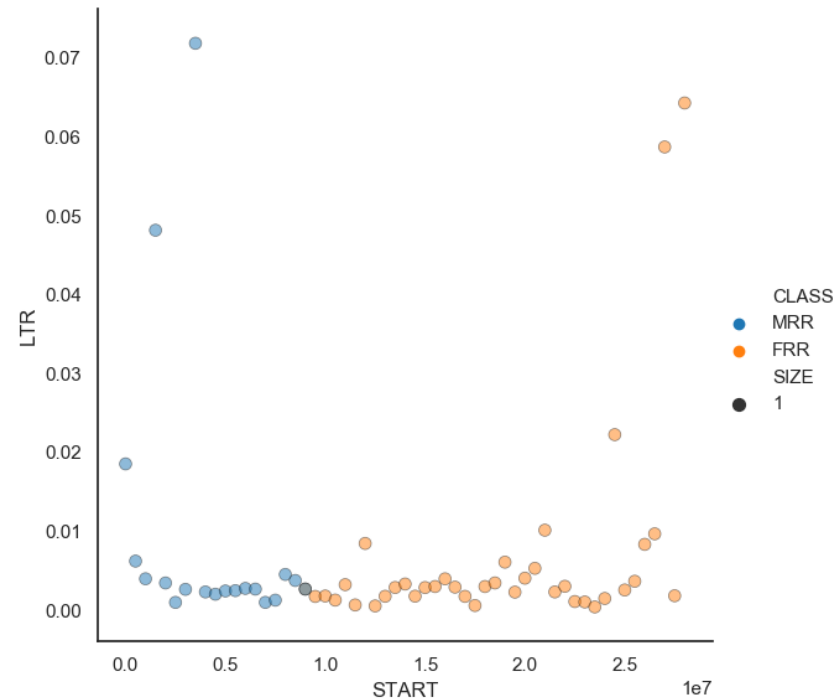

chr 20

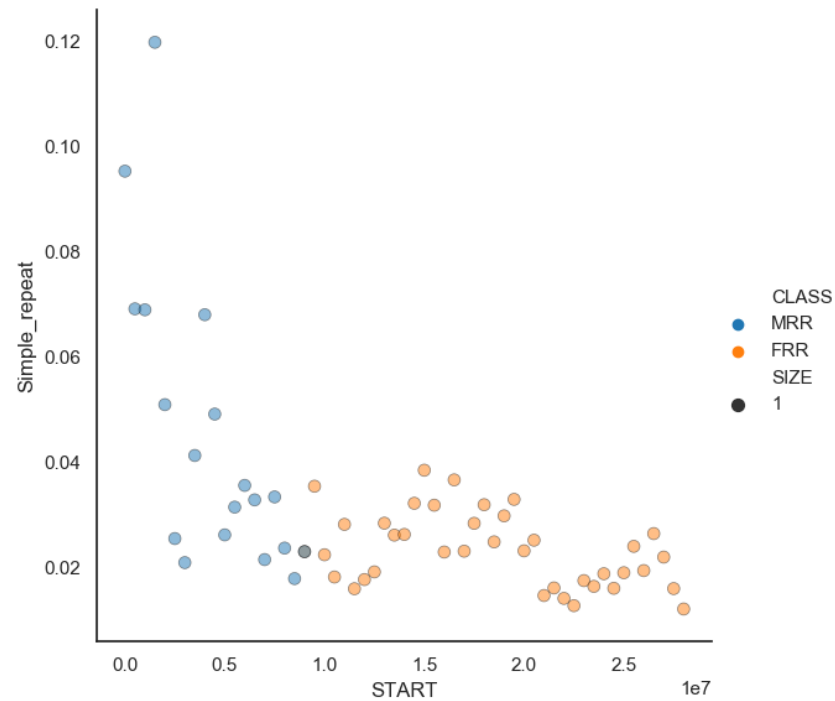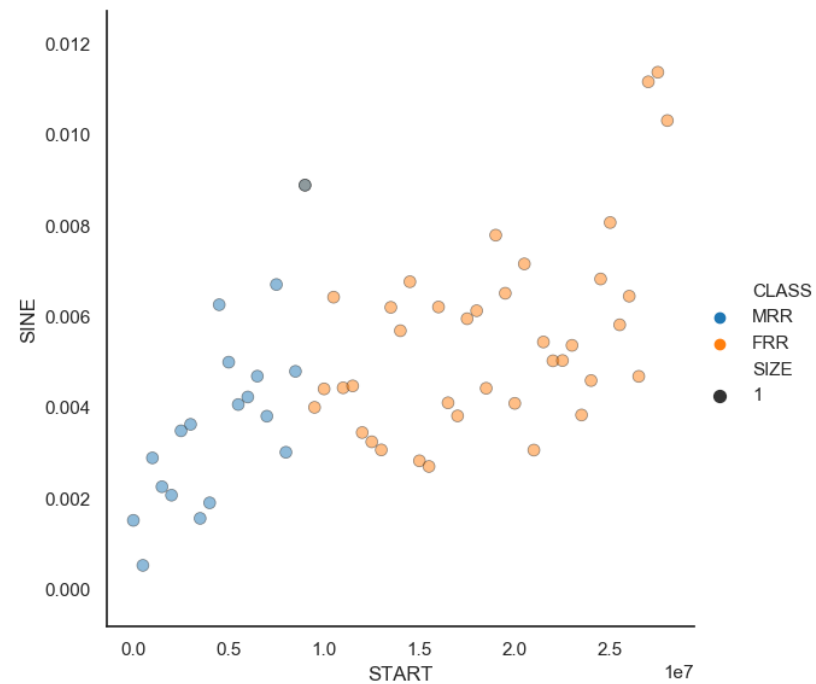

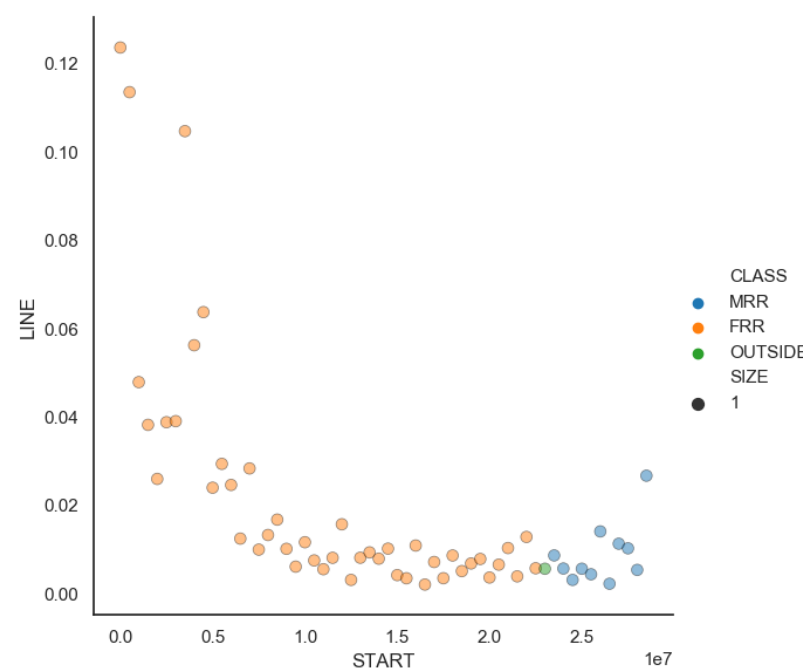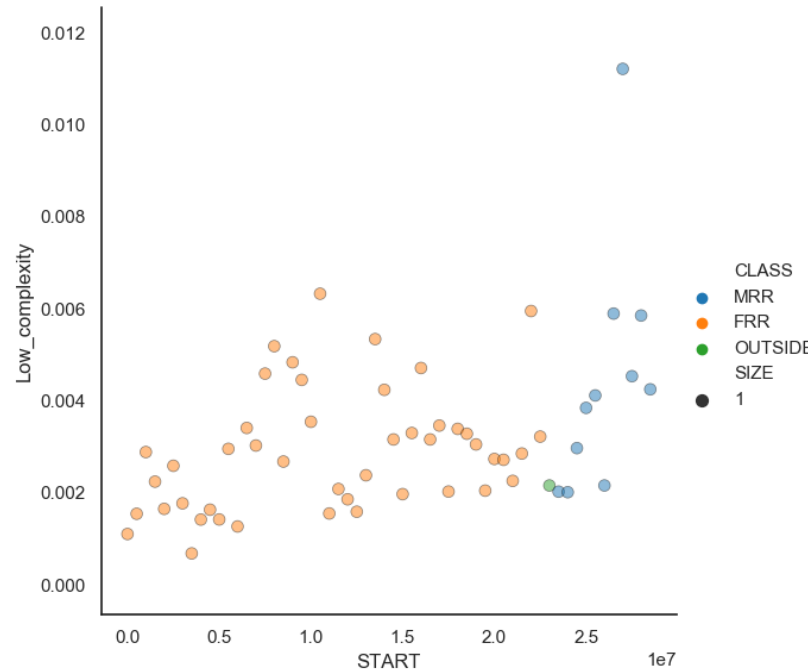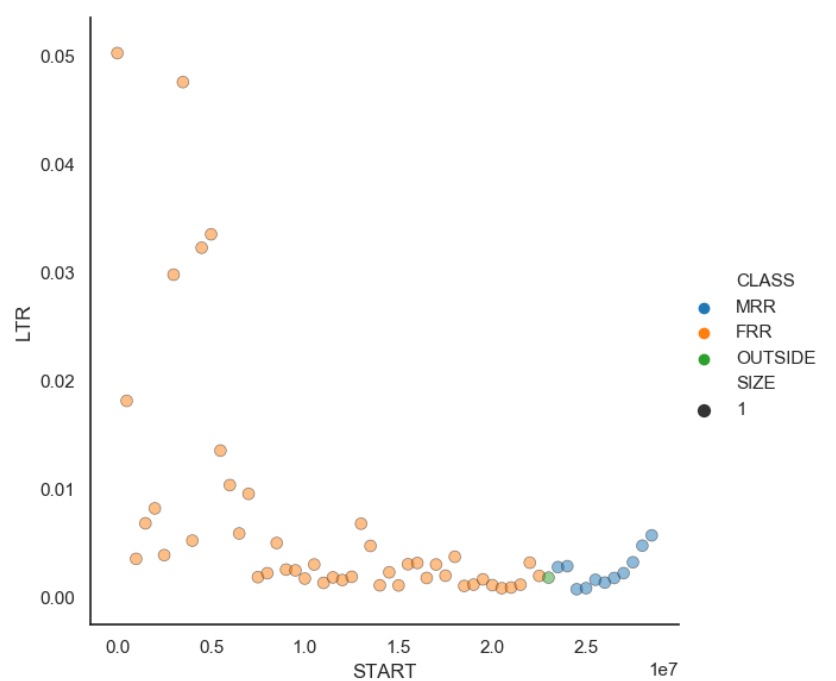

chr 21

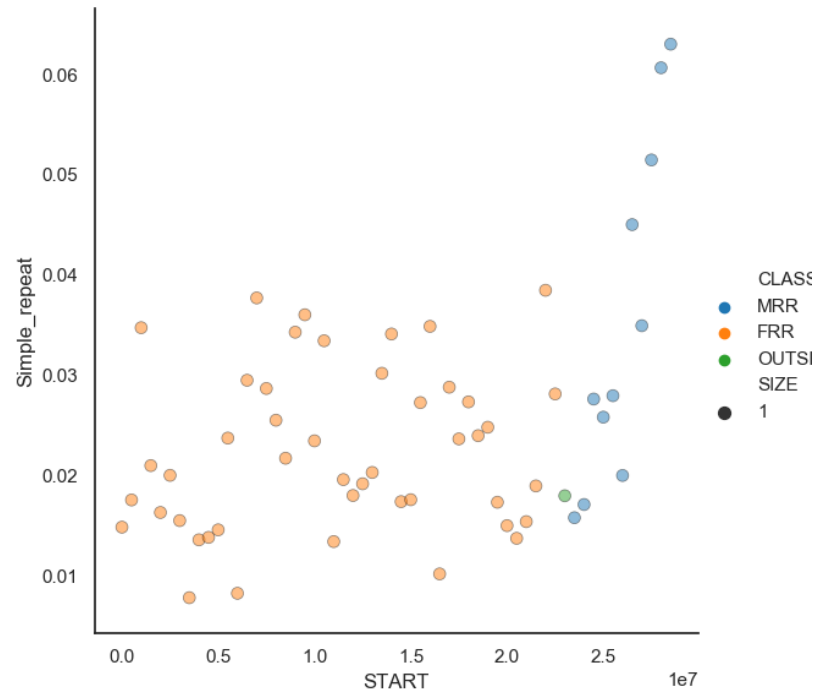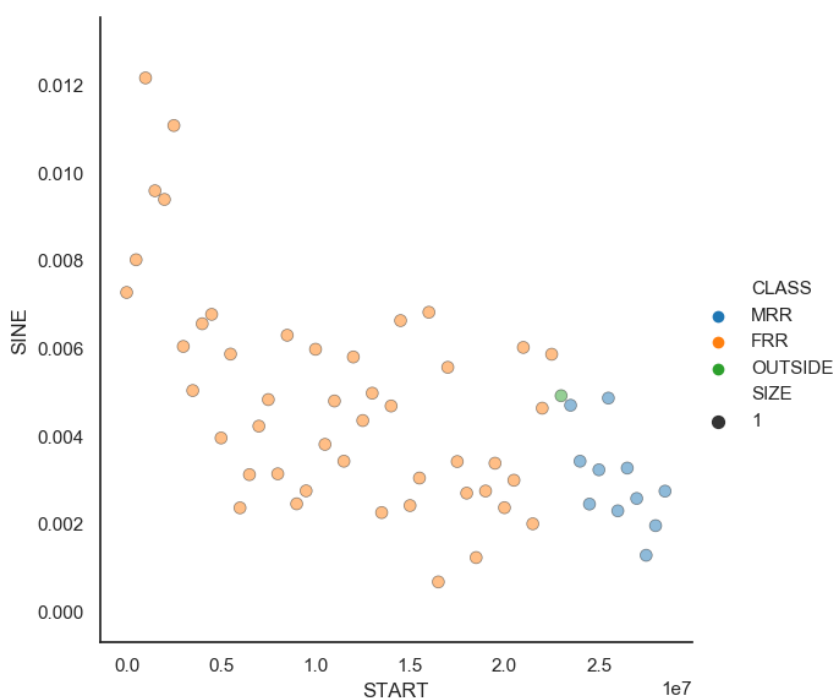

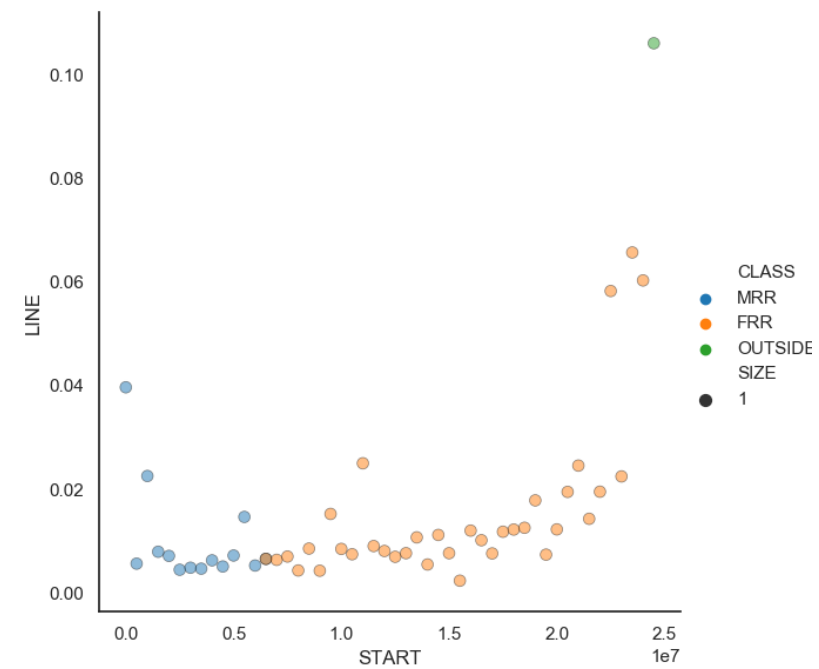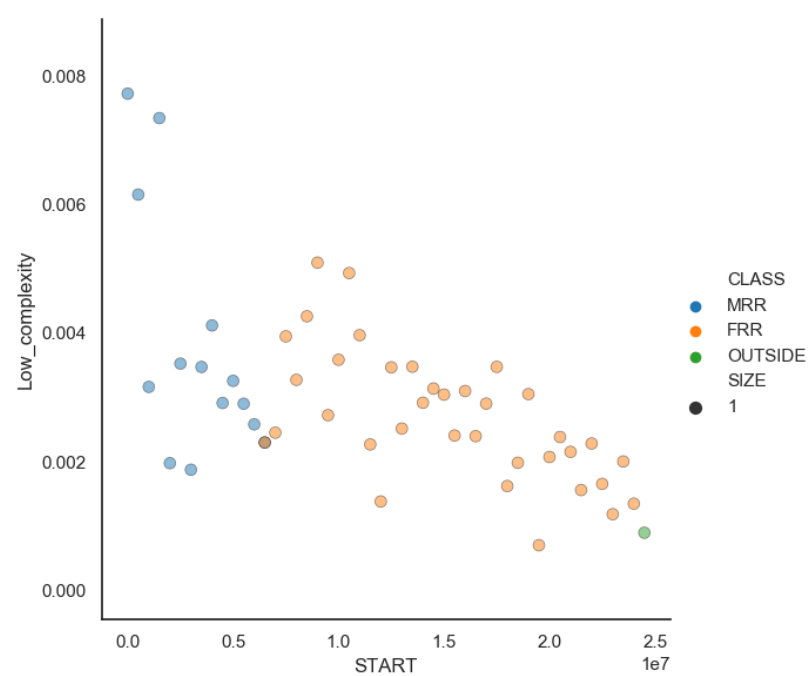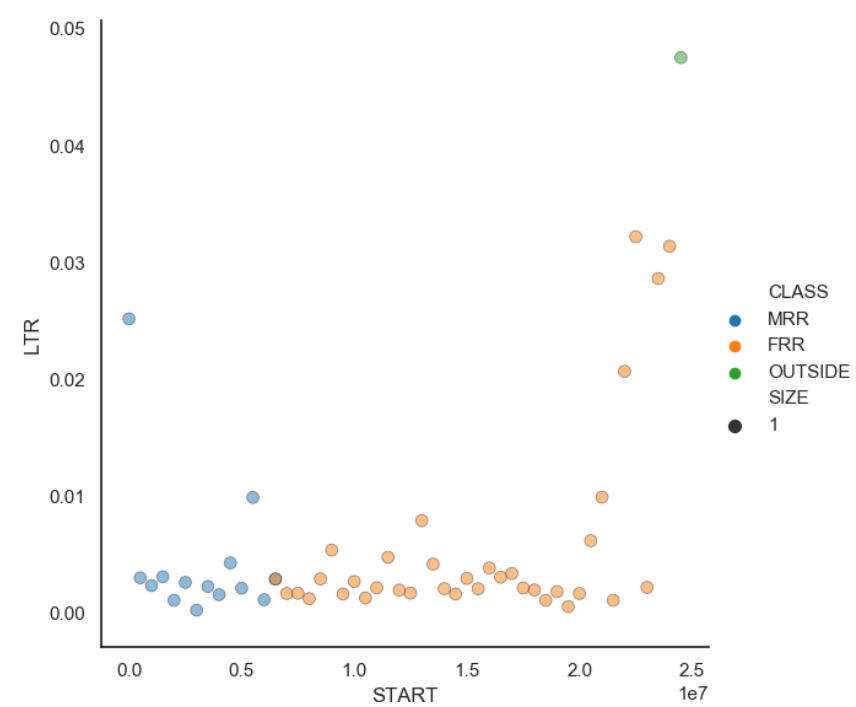

chr 22

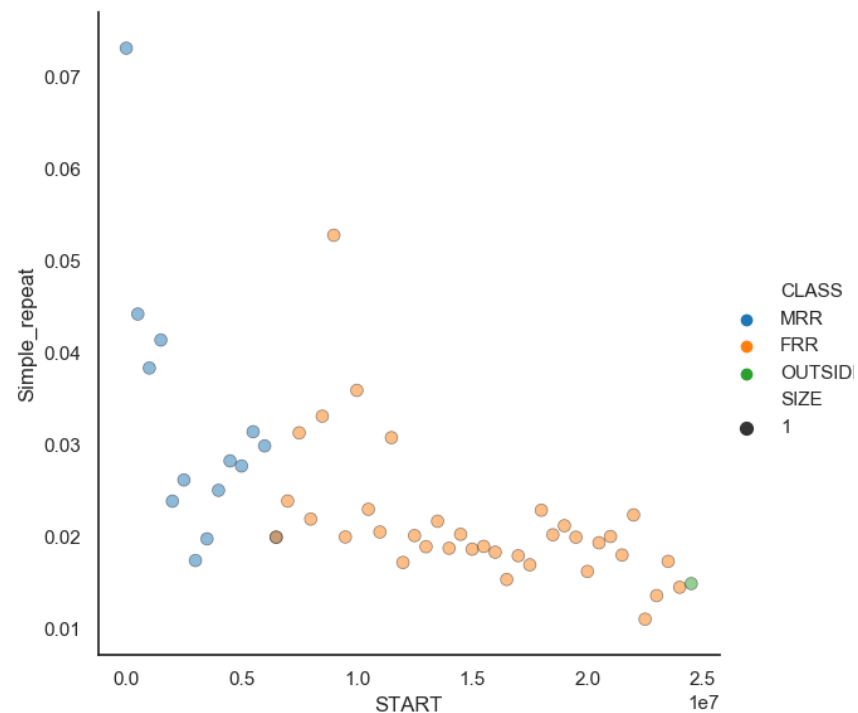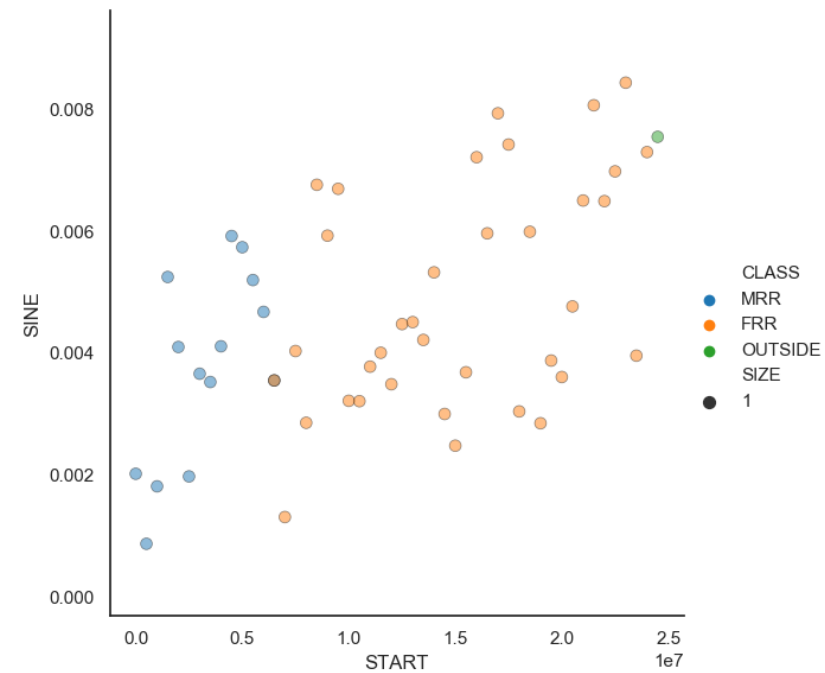

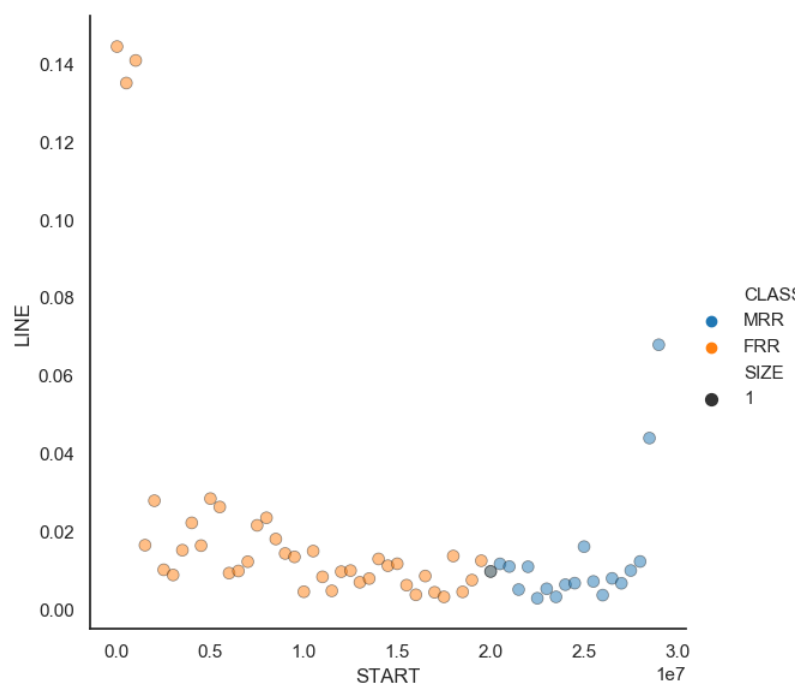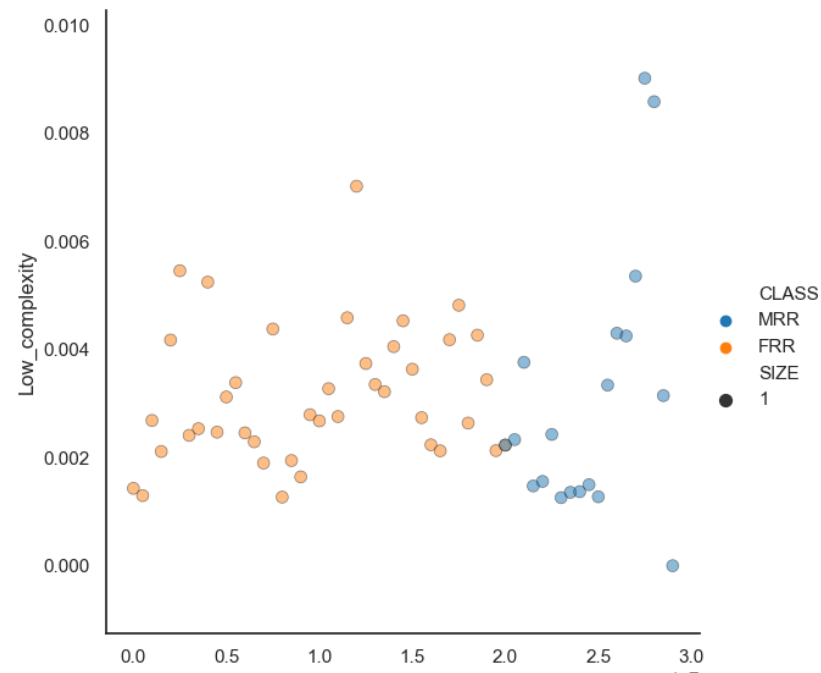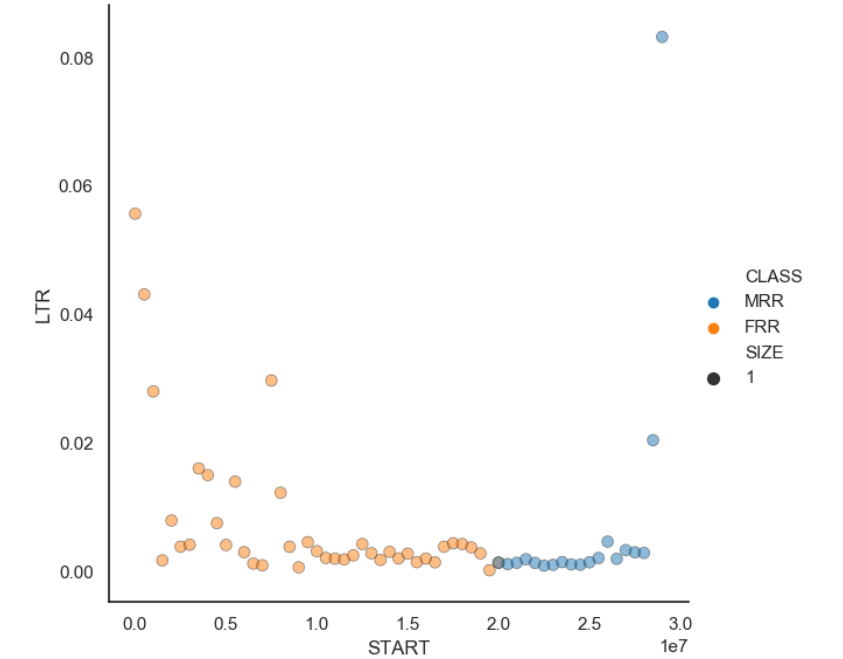

chr 23

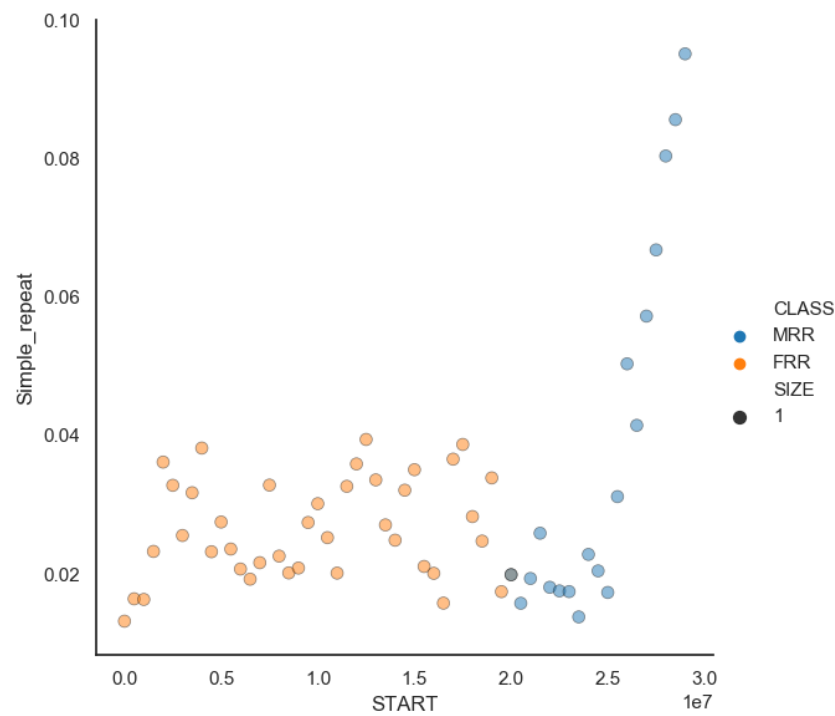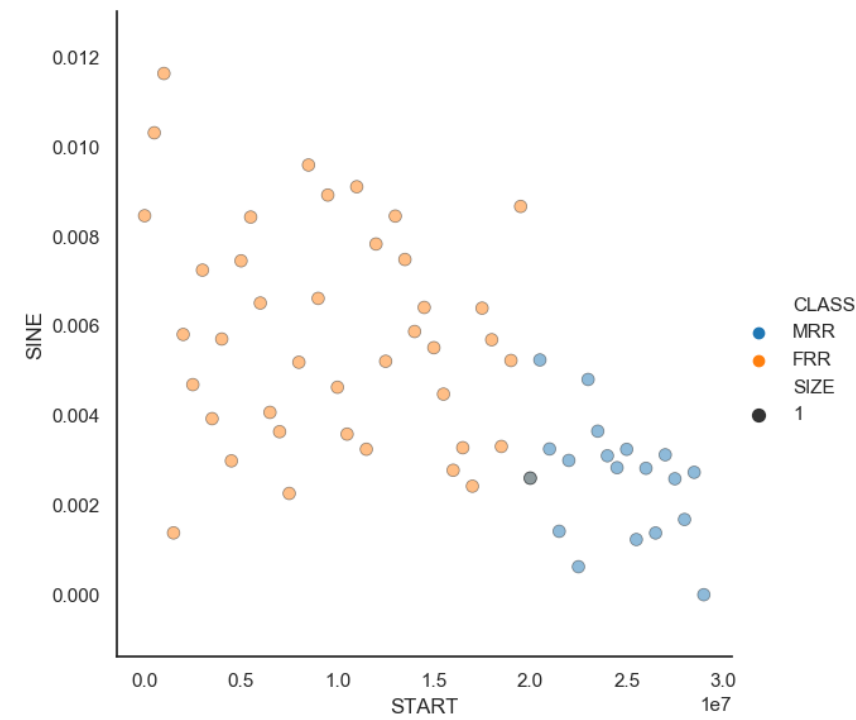

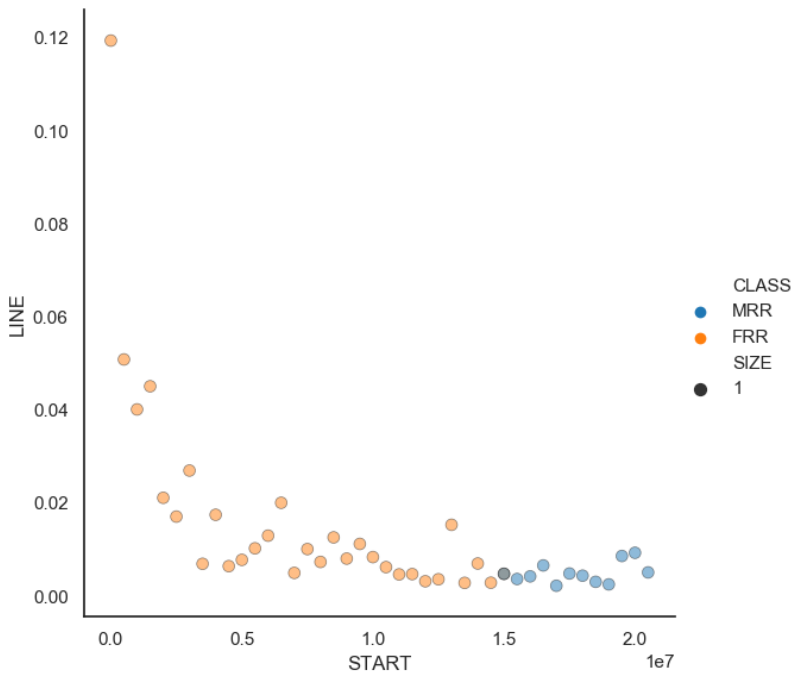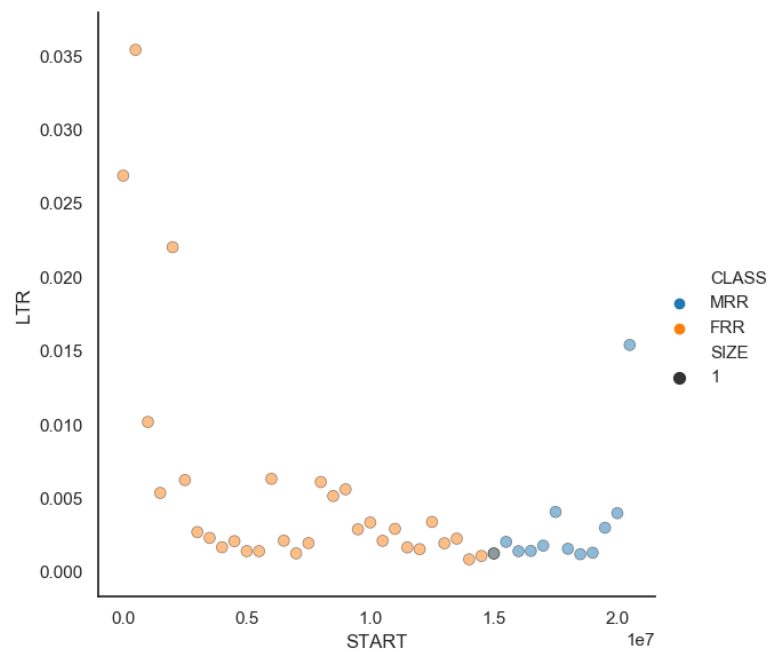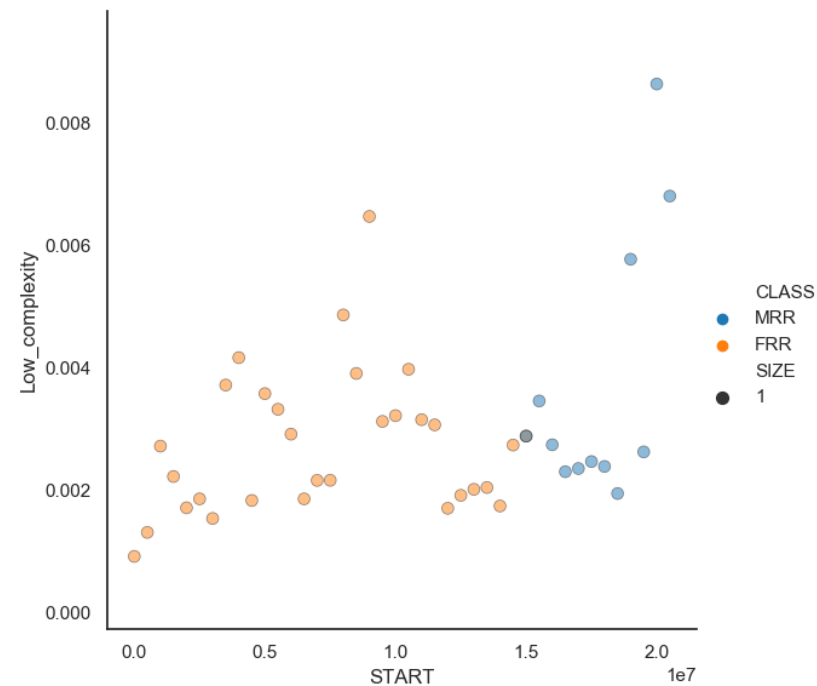

chr 24

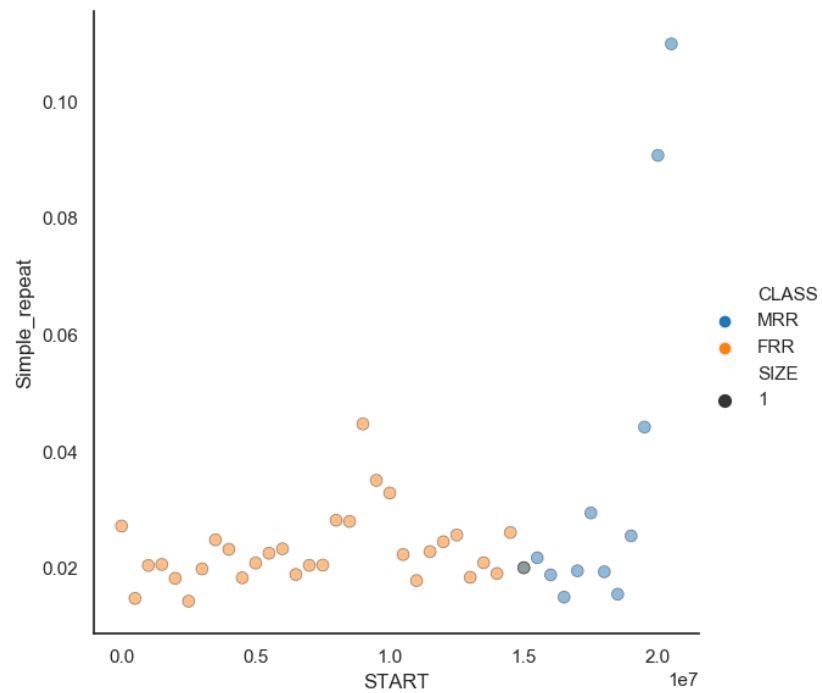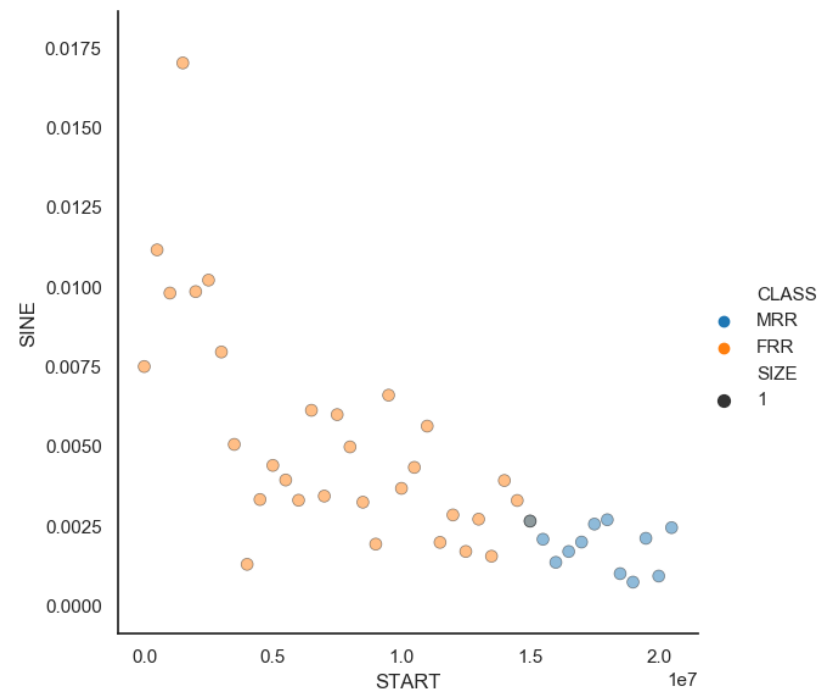

Supplement: S10 Fig — X-axes show coordinates along the chromosome (107 bp). Y-axes show the proportion of nucleotides in 500 kb windows overlapping an element belonging to each specific repeat superclass. Repeat superclass is indicated to the left of each subplot. Circle colors indicate whether a 500 kb window is classified as a MRR or FRR. (PDF) [file pgen.1010011.s010.pdf]

Supplementary Fig. 13

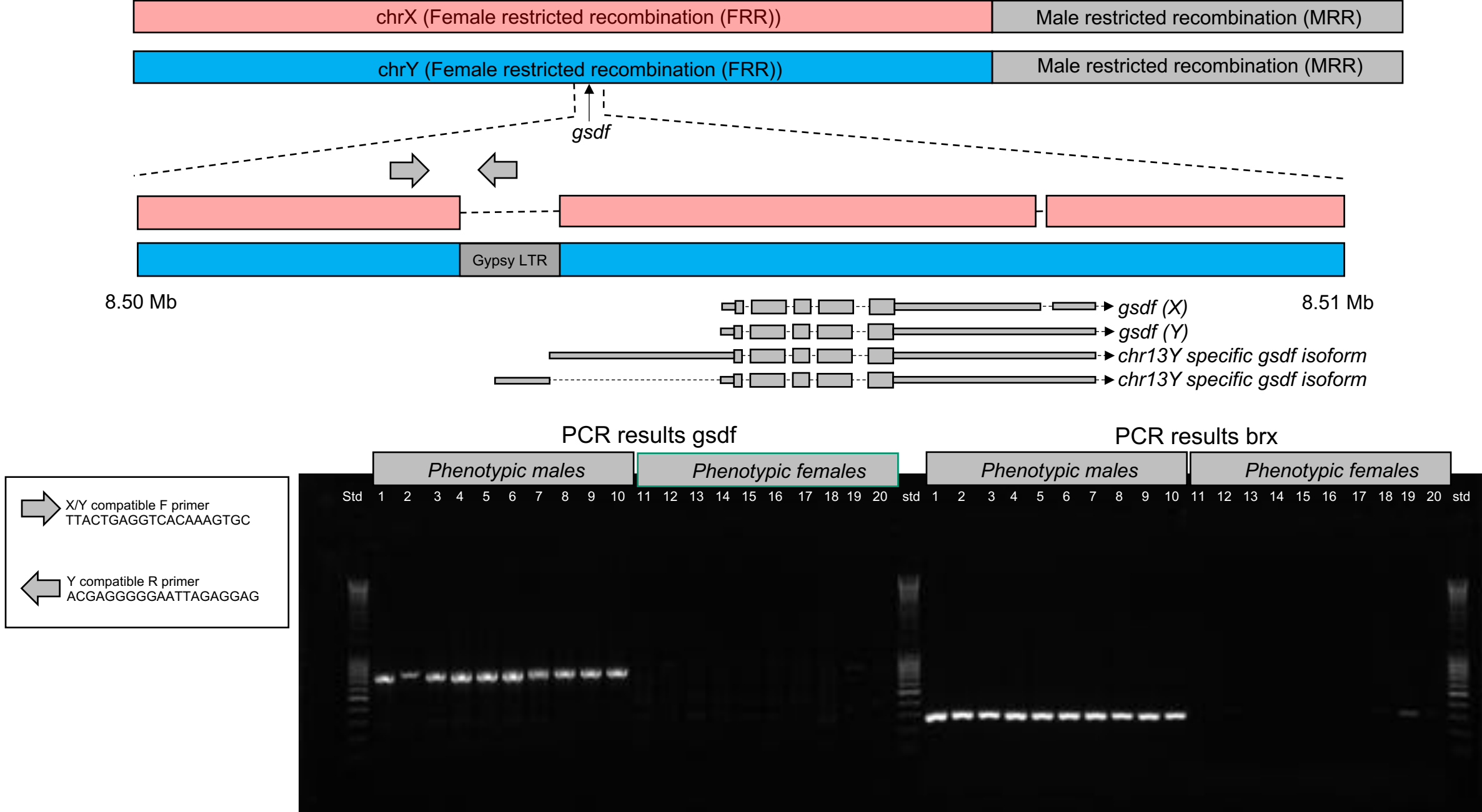

Supplement: S13 Fig — The forward gsdf primer is 481 nt upstream of the 1.2 kb chrY Gypsy-LTR insertion. The reverse primer is 251 nt into the LTR. The brx primers are described in Methods. The resulting PCR products are shown in an agarose gel. The same 20 individuals are shown for both primer pairs. Individuals 1–10 are phenotypic males and 11–20 are phenotypic females. PCR results show that brx classification and presence of Gypsy-LTR insertion are in agreement and distinguish all 10 males from females. (PDF) [file pgen.1010011.s013.pdf]
