# Supplementary material for: Heterochiasmy and the establishment of gsdf as a novel sex determining gene in Atlantic halibut
Source: PLoS Genet. 2022 Feb 8;18(2):e1010011. doi: 10.1371/journal.pgen.1010011 (PMC8824383; doi:10.1371/journal.pgen.1010011)

Supplementary Fig. 11

HiC contact maps (all chromosomes and chr13 separately) of the male (XY) Atlantic halibut used to generate the genome assembly. No apparent large-scale inversions or translocations were observed (would appear as off-diagonal contacts)

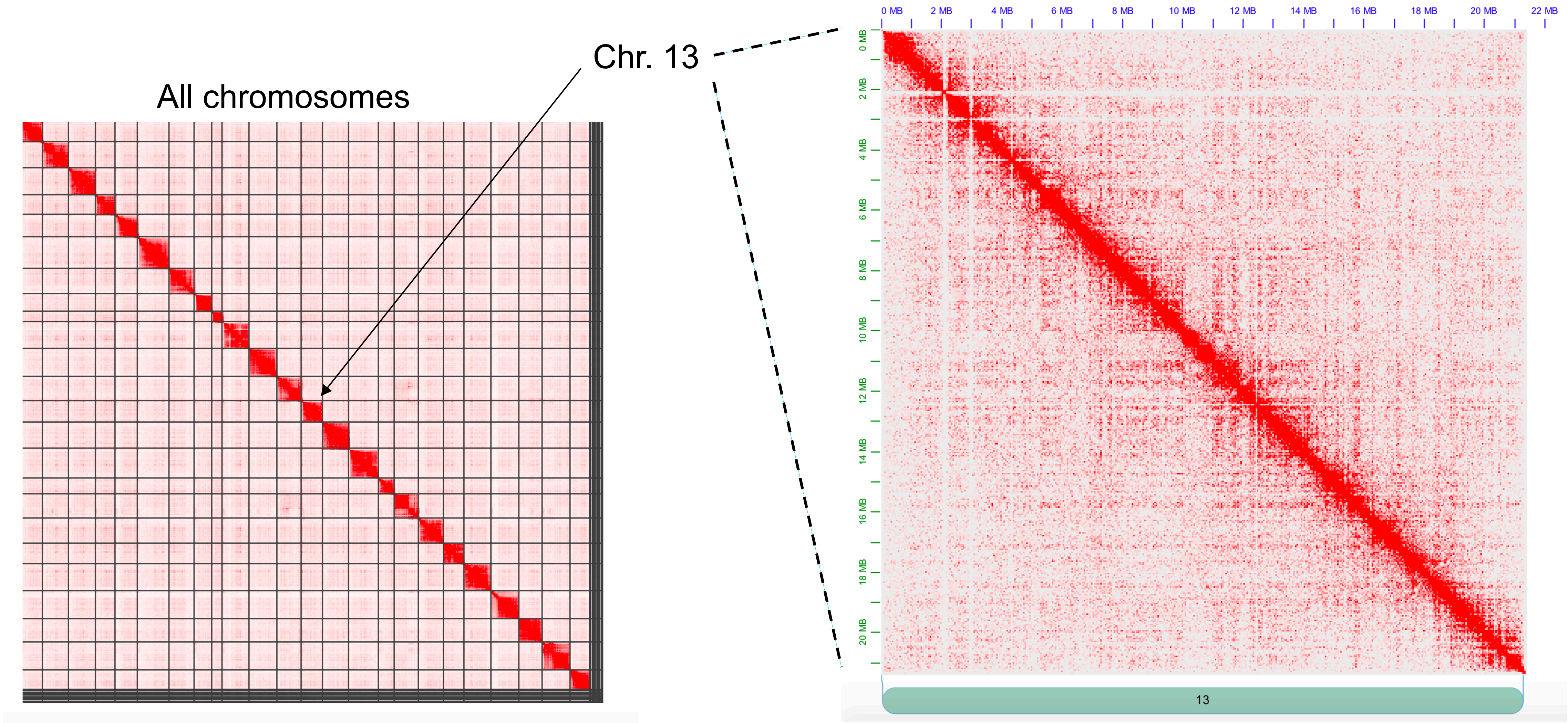

Supplement: S11 Fig — No apparent large-scale inversions or translocations were observed (would appear as off-diagonal contacts) (PDF) [file pgen.1010011.s011.pdf]
