# Supplementary material for: Heterochiasmy and the establishment of gsdf as a novel sex determining gene in Atlantic halibut
Source: PLoS Genet. 2022 Feb 8;18(2):e1010011. doi: 10.1371/journal.pgen.1010011 (PMC8824383; doi:10.1371/journal.pgen.1010011)

Supplementary Fig. 12:
Chr. Y carries a transposon-derived LTR upstream of gsdf. Male- and female haplotypes differ for a 1.2 kb transposable element derived segment 2 kb upstream of *gsdf* transcription start site of. Males carry the insertion allele which contains a lowly methylated CpG-island inferred to act as a derived promoter of *gsdf* uniquely in males. Shown are alignments of male- and female pool-seq data, nanopore reads and supernova pseudo-haplotype assemblies from the single male used to create the IMR_Hiphip.v1 assembly. A heterozygote 1.2 kb insertion is evident in nanopore read alignments as well as in the linked-read haplotype alignments and chr13Y carries the insertion allele. The insertion is less evident in the male short-read data due to poor mappability in the in/del region. Male pool short reads carrying chr13Y haplotype variant tags have fever red colored mates supporting the deletion allele (short read alignments are sorted by the SNP allele in the center red/blue box, blue boxes= chrY-allele, red boxes=chrX-allele). *gsdf* gene model in blue shows the extent of *gsdf* expression detected in Atlantic halibut adult testis and adult ovary and testis in Pacific halibut. The two *gsdf* gene models in red indicate isoforms detected only in males from three separate developmental stages.

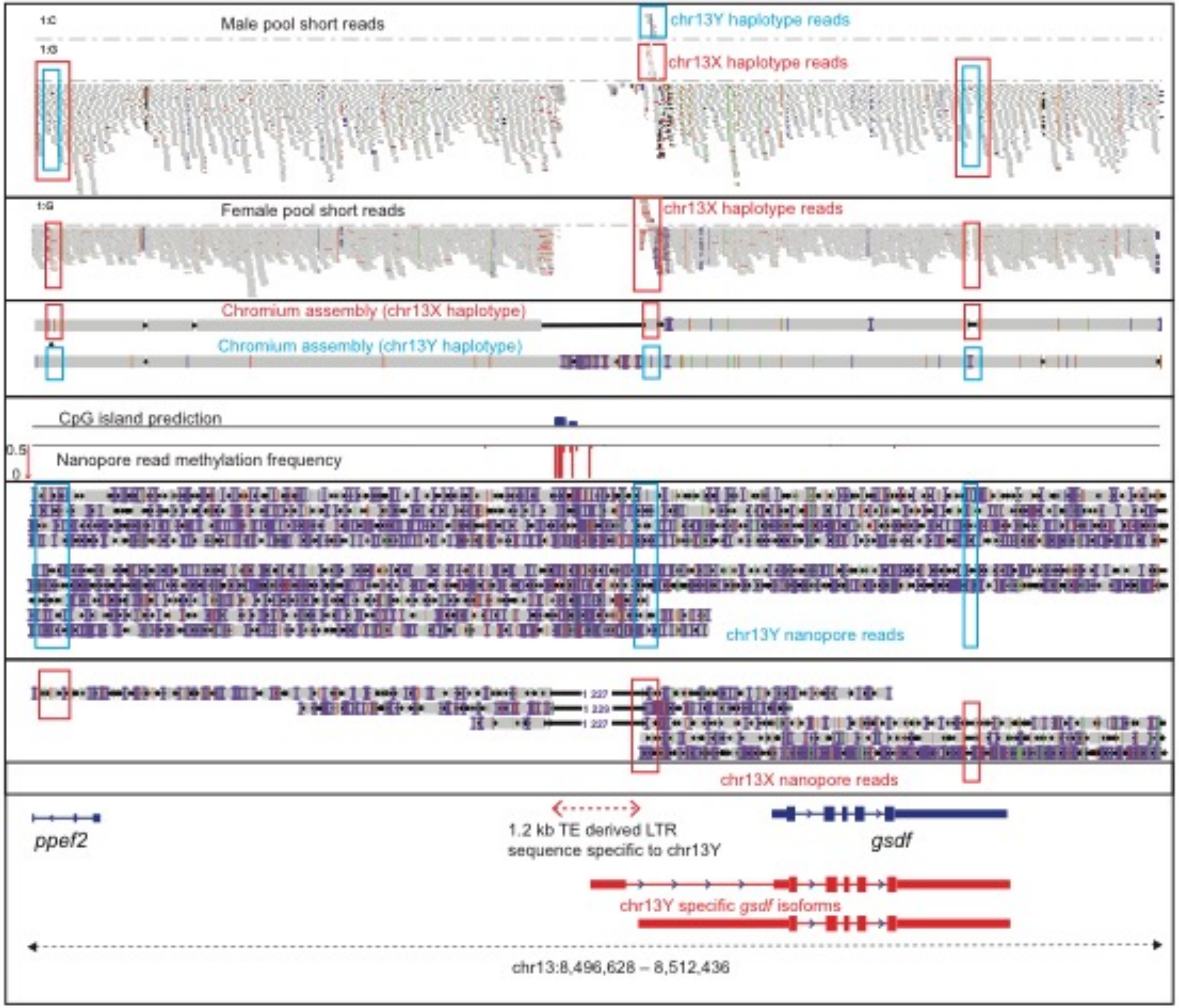

Supplement: S12 Fig — Male- and female haplotypes differ for a 1.2 kb transposable element derived segment 2 kb upstream of gsdf transcription start site of. Males carry the insertion allele which contains a lowly methylated CpG-island inferred to act as a derived promoter of gsdf uniquely in males. Shown are alignments of male- and female pool-seq data, nanopore reads and supernova pseudo-haplotype assemblies from the single male used to create the IMR_Hiphip.v1 assembly. A heterozygote 1.2 kb insertion is evident in nanopore read alignments as well as in the linked-read haplotype alignments and chr13Y carries the insertion allele. The insertion is less evident in the male short-read data due to poor mappability in the in/del region. Male pool short reads carrying chr13Y haplotype variant tags have fever red colored mates supporting the deletion allele (short read alignments are sorted by the SNP allele in the center red/blue box, blue boxes = chrY-allele, red boxes = chrX-allele). gsdf gene model in blue shows the extent of gsdf expression detected in Atlantic halibut adult testis and adult ovary and testis in Pacific halibut. The two gsdf gene models in red indicate isoforms detected only in males from three separate developmental stages. (PDF) [file pgen.1010011.s012.pdf]
