# Supplementary material for: Heterochiasmy and the establishment of gsdf as a novel sex determining gene in Atlantic halibut
Source: PLoS Genet. 2022 Feb 8;18(2):e1010011. doi: 10.1371/journal.pgen.1010011 (PMC8824383; doi:10.1371/journal.pgen.1010011)
Supplement: S14 Fig — A The Chromium pseudo-haplotype assemblies of the reference genome male individual represents chrY (pseudo-haplotype 1) and chrX (pseudo-haplotype 2) for the interval chr13:0–9 Mb. The Chromium scaffold ID is number 45 for both PH1 and PH2. Our assembly of Atlantic halibut chr13 is inverted in comparison with the Chromium pseudo-haplotypes as well as the syntenic Pacific halibut chromosome (CM023513.1). Syntenic regions corresponding to the location of the Atlantic halibut chrY-core (chr13:0–9 Mb) are indicated in the Figure. B Counts of SNP alleles uniquely observed in each of the Atlantic halibut pseudo-haplotype assemblies in the core X/Y region and in the corresponding region of Pacific halibut chromosome CM023513.1. It is clear that the overall divergence, as well as non-synonymous and synonymous divergence is higher between species than between Atlantic halibut X and. Y. C The table shows counts of missense and synonymous variants observed in pseudo-haplotypes 1 and 2 (PH1 and PH2) in reation to Pacific halibut gene models. Counts per Mb are shown in Pacific halibut coordinates along the syntenic chromosome CM023513.1. Only variants differing between PH1 and PH2 were included. (PDF) [file pgen.1010011.s014.pdf]

Supplementary Fig. 14: Atlantic halibut chrY and chrX carry similar numbers of missense variants in relation to Pacific halibut.
a. The Chromium pseudo-haplotype assemblies of the reference genome male individual represents chrY (pseudo-haplotype 1) and chrX (pseudo-haplotype 2) for the interval chr13:0–9 Mb. The Chromium scaffold ID is number 45 for both PH1 and PH2. Our assembly of Atlantic halibut chr13 is inverted in comparison with the Chromium pseudo-haplotypes as well as the syntenic Pacific halibut chromosome (CM023513.1). Syntenic regions corresponding to the location of the Atlantic halibut chrY-core (chr13:0-9 Mb) are indicated in the Figure. **b.** Counts of SNP alleles uniquely observed in each of the Atlantic halibut pseudo-haplotype assemblies in the core X/Y region and in the corresponding region of Pacific halibut chromosome CM023513.1. It is clear that the overall divergence, as well as non-synonymous and synonymous divergence is higher between species than between Atlantic halibut X and. Y. **c.** The table shows counts of missense and synonymous variants observed in pseudo-haplotypes 1 and 2 (PH1 and PH2) in reation to Pacific halibut gene models. Counts per Mb are shown in Pacific halibut coordinates along the syntenic chromosome CM023513.1. Only variants differing between PH1 and PH2 were included.

a

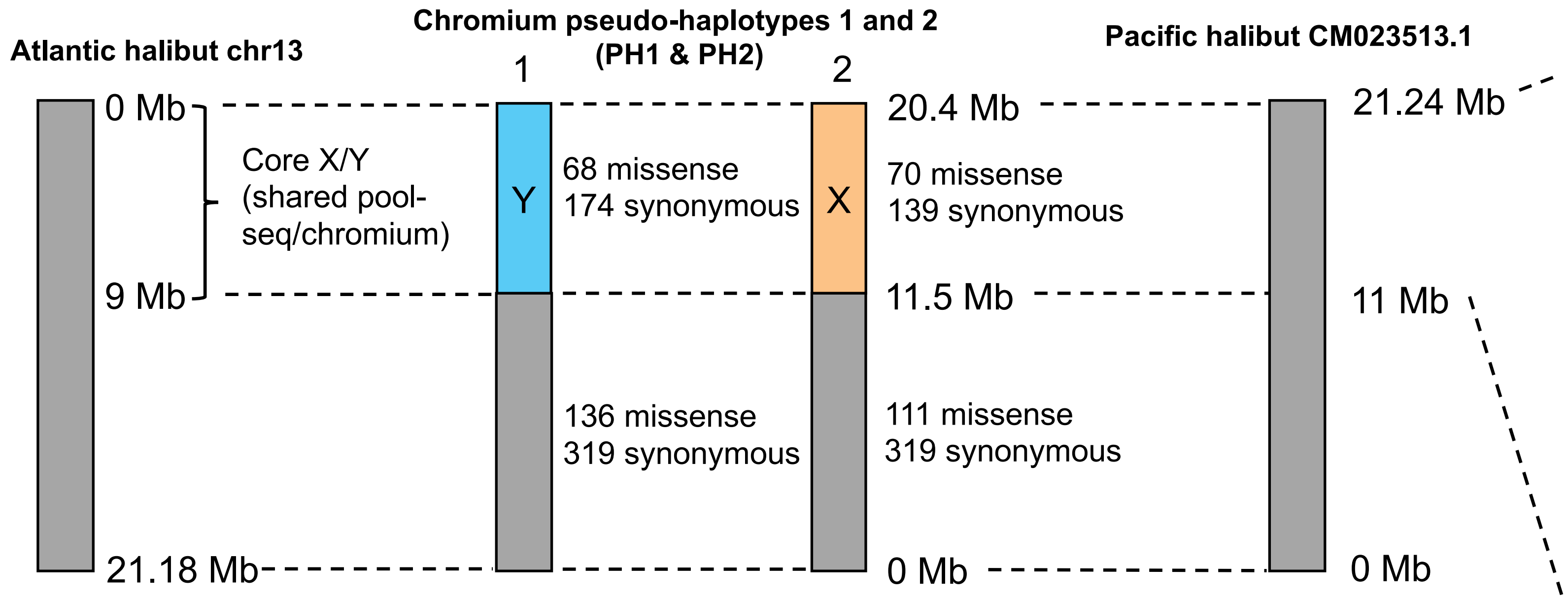

b

Allele uniquely observed in	Shared SNPs	Shared synonymous SNPs	Shared missense SNPs
CM023513.1 (Pacific halibut)	40579	810	419
PH2 (chrX)	7666	139	70
PH1 (chrY)	7611	174	68

c

	0-1Mb	1-2Mb	2-3Mb	3-4Mb	4-5Mb	5-6Mb	6-7Mb	7-8Mb	8-9Mb	9-10Mb	10-11Mb	11-12Mb	12-13Mb	13-14Mb	14-15Mb	15-16Mb	16-17Mb	17-18Mb	18-19Mb	19-20Mb	20-21Mb
PH2 missense	20	23	24	10	2	2	6	2	7	9	6	0	8	15	4	4	13	7	7	8	4
PH1 missense	13	34	34	9	6	4	5	3	12	12	4	0	14	15	4	5	14	6	5	4	1
PH2 synonymous	23	66	83	33	16	23	12	8	21	19	15	2	30	31	9	9	6	21	12	12	7
PH1 synonymous	22	59	82	31	23	23	21	11	21	19	7	1	30	47	14	8	21	15	18	9	11
