# Supplementary material for: Heterochiasmy and the establishment of gsdf as a novel sex determining gene in Atlantic halibut
Source: PLoS Genet. 2022 Feb 8;18(2):e1010011. doi: 10.1371/journal.pgen.1010011 (PMC8824383; doi:10.1371/journal.pgen.1010011)

Supplementary Fig. 15:
Allelic imbalance on chr13.
Among X/Y differentiating pool-seq SNPs where different alleles were observed also in male and female RNA-seq (Supplementary Fig. 4a), 130 SNPs were retained due to both the X and Y alleles being observed in at least one read in at least 6/7 male samples. Out of these 130 SNPs, six displayed allelic imbalance in males (defined as all seven male samples expressing one allele in higher levels). For three of these six (in the genes FLOWR, WDR5 and an unknown transcript) the imbalance is towards the ChrX-allele, while for three (CKMT2, *gsdf* and an unknown transcript) the imbalance is towards the ChrY-allele. Nucleotide positions of SNPs are shown to the right of the heat-map. Figure generated in https://software.broadinstitute.org/morpheus/

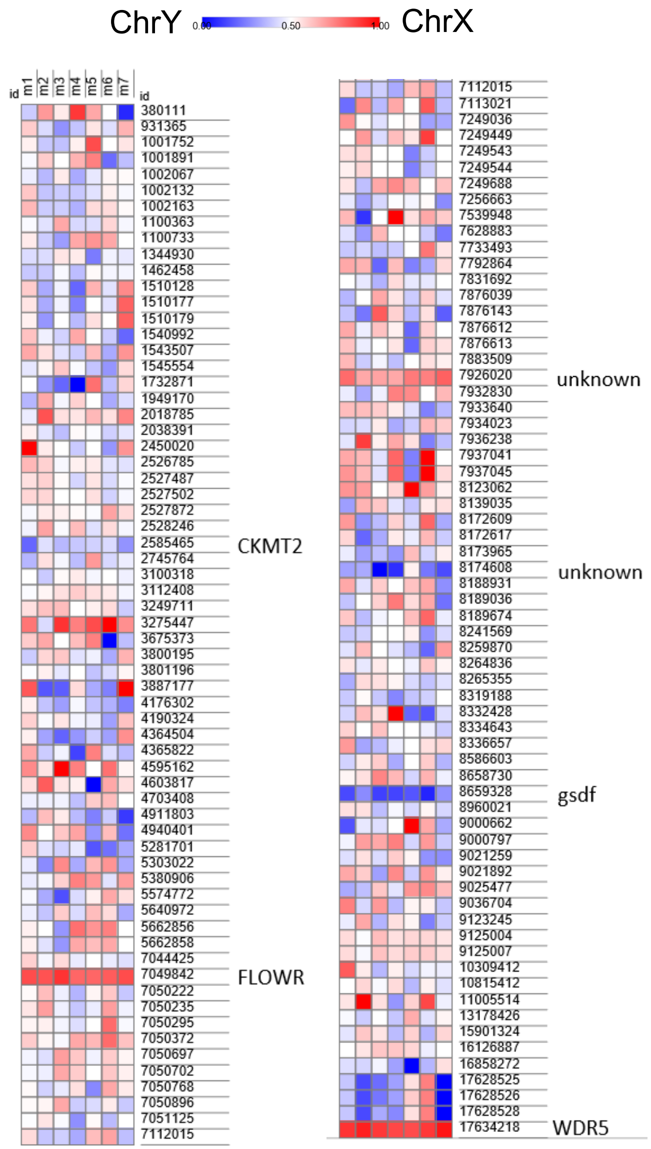

Supplement: S15 Fig — Among X/Y differentiating pool-seq SNPs where different alleles were observed also in male and female RNA-seq (S4A Fig), 130 SNPs were retained due to both the X and Y alleles being observed in at least one read in at least 6/7 male samples. Out of these 130 SNPs, six displayed allelic imbalance in males (defined as all seven male samples expressing one allele in higher levels). For three of these six (in the genes FLOWR, WDR5 and an unknown transcript) the imbalance is towards the ChrX-allele, while for three (CKMT2, gsdf and an unknown transcript) the imbalance is towards the ChrY-allele. Nucleotide positions of SNPs are shown to the right of the heat-map. Figure generated in https://software.broadinstitute.org/morpheus/. (PDF) [file pgen.1010011.s015.pdf]
