# Supplementary material for: Heterochiasmy and the establishment of gsdf as a novel sex determining gene in Atlantic halibut
Source: PLoS Genet. 2022 Feb 8;18(2):e1010011. doi: 10.1371/journal.pgen.1010011 (PMC8824383; doi:10.1371/journal.pgen.1010011)
Supplement: S16 Fig — This model assumes linkage between sexually antagonistic (SA) loci and the sex determining gene being important. On top, three fictive autosome pairs (A1-A3) are shown. One of these acquires genetic material governing development of carriers into phenotypic males (left arrow) or phenotypic females (right arrow), thereby creating a novel sex chromosome. Thick black lines in between chromatids indicate the chromosomal interval undergoing meiotic recombination in each sex. The new sex determining gene (*) must occur in a region incapable of meiotic recombination in the heterogametic sex, or else, it does not become isolated from recombining with its former autosomal pair during meiosis. Arrows within boxes next to the sex determining gene location indicate the interval where a new sex determining gene could occur in the two respective systems, (XY or ZW). (PDF) [file pgen.1010011.s016.pdf]

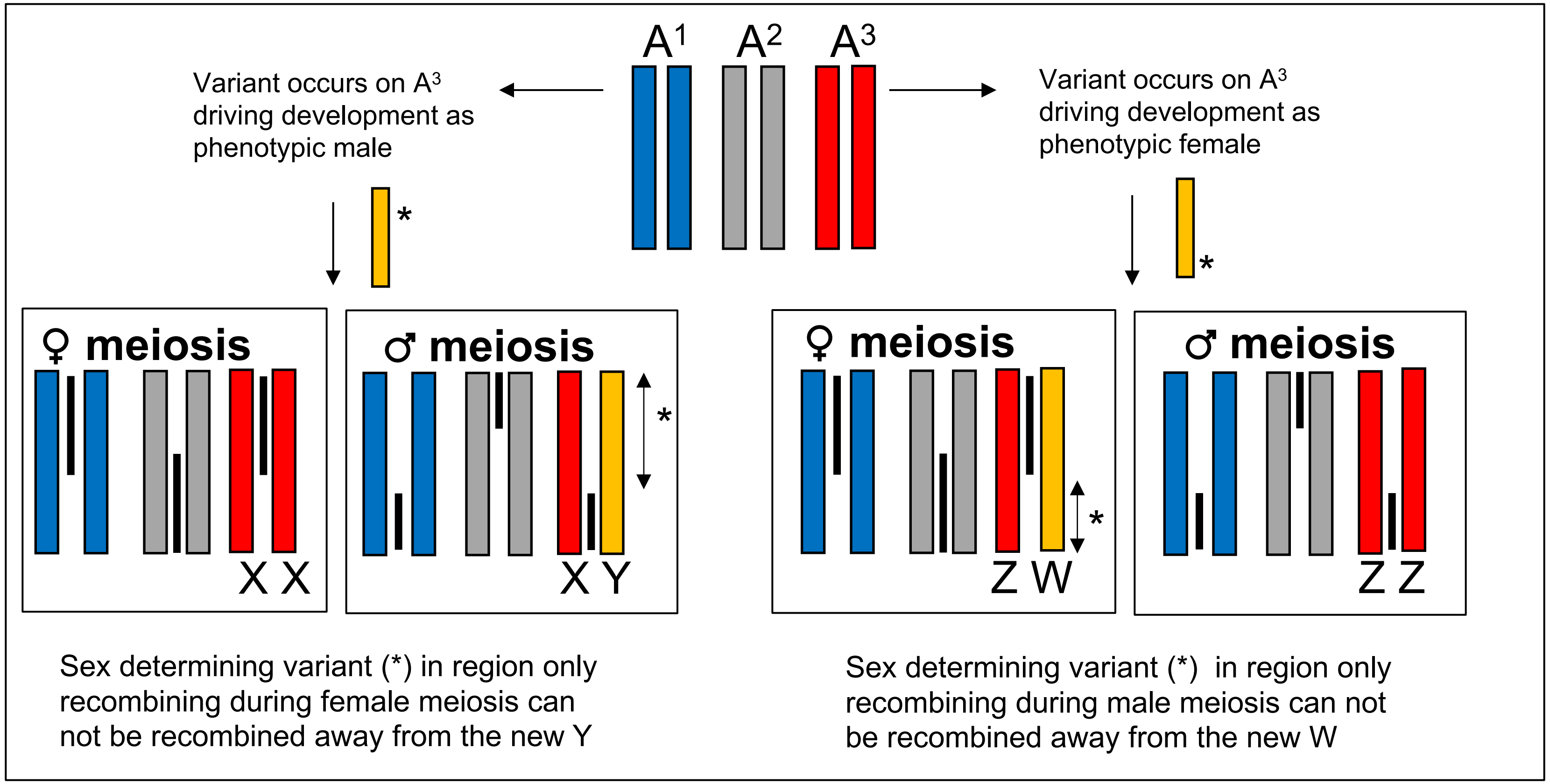

Supplementary Fig. 16: Theoretical evolution of sex chromosomes in a heterochiasmy setting. This model assumes linkage between sexually antagonistic (SA) loci and the sex determining gene being important. On top, three fictive autosome pairs (A^1-A^3) are shown. One of these acquires genetic material governing development of carriers into phenotypic males (left arrow) or phenotypic females (right arrow), thereby creating a novel sex chromosome. Thick black lines in between chromatids indicate the chromosomal interval undergoing meiotic recombination in each sex. The new sex determining gene (*) must occur in a region incapable of meiotic recombination in the heterogametic sex, or else, it does not become isolated from recombining with its former autosomal pair during meiosis. Arrows within boxes next to the sex determining gene location indicate the interval where a new sex determining gene could occur in the two respective systems, (XY or ZW).
