# Supplementary material for: Heterochiasmy and the establishment of gsdf as a novel sex determining gene in Atlantic halibut
Source: PLoS Genet. 2022 Feb 8;18(2):e1010011. doi: 10.1371/journal.pgen.1010011 (PMC8824383; doi:10.1371/journal.pgen.1010011)

Supplementary Fig. 17: Reference gene for qPCR. To find a suitable (stable) internal reference gene for the normalization of the qPCR data, we selected a few candidates from the RNAseq dataset with a stable number of reads throughout the developmental stages available. **a** Expression of the selected genes *ints9*, *JKAMP* and *gtf3c6* in Atlantic halibut embryos and larvae, shown as number of reads. Data are shown as mean with SEM. N=4. **b** The selected genes *ints9*, *JKAMP* and *gtf3c6* were selected for testing with qPCR on the actual samples to be analyzed for *gsdf* expression. Expression of in Atlantic halibut embryos and larvae, shown as cycle threshold (CT)-values. Data are shown as mean with SEM. N=4-9. *gtf3c6* had the most stable expression throughout the developmental stages, and was therefore chosen as the internal reference gene in this study.

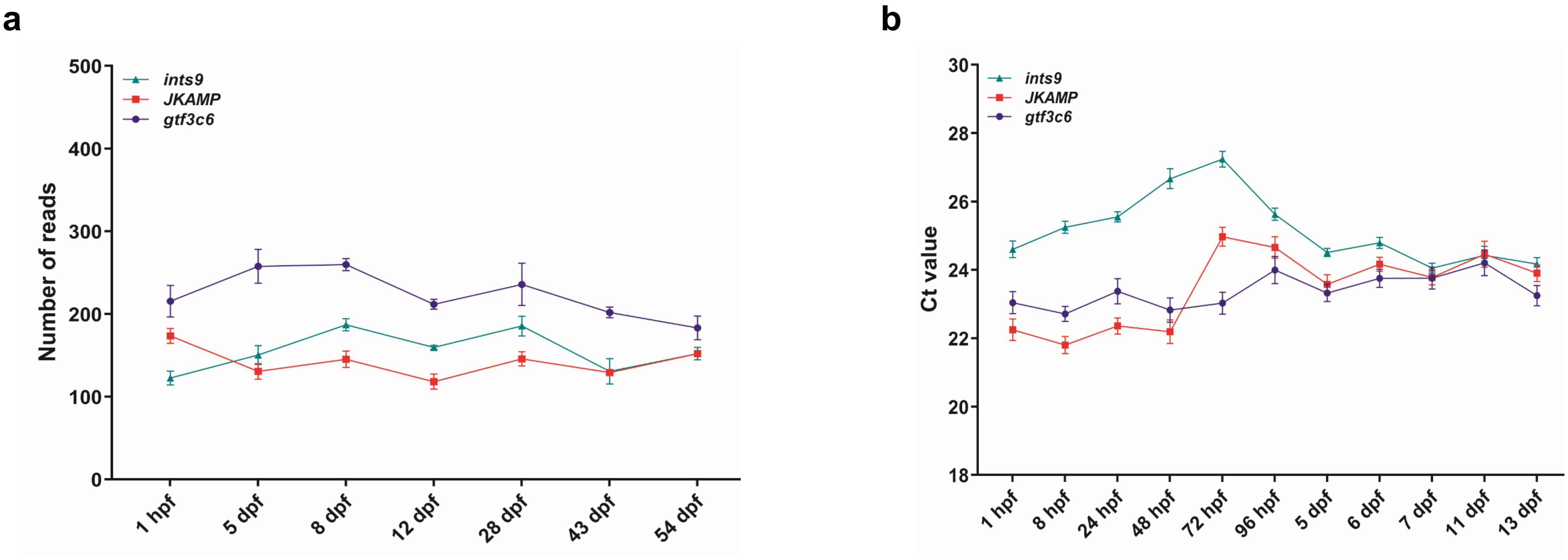

Supplement: S17 Fig — To find a suitable (stable) internal reference gene for the normalization of the qPCR data, we selected a few candidates from the RNA-seq dataset with a stable number of reads throughout the developmental stages available. A Expression of the selected genes ints9, JKAMP and gtf3c6 in Atlantic halibut embryos and larvae, shown as number of reads. Data are shown as mean with SEM. N = 4. B The selected genes ints9, JKAMP and gtf3c6 were selected for testing with qPCR on the actual samples to be analyzed for gsdf expression. Expression of in Atlantic halibut embryos and larvae, shown as cycle threshold (CT)-values. Data are shown as mean with SEM. N = 4–9. gtf3c6 had the most stable expression throughout the developmental stages, and was therefore chosen as the internal reference gene in this study. (PDF) [file pgen.1010011.s017.pdf]
